# Supplementary material for: Reaction performance prediction with an extrapolative and interpretable graph model based on chemical knowledge
Source: Nat Commun. 2023 Jun 15;14:3569. doi: 10.1038/s41467-023-39283-x (PMC10272164; doi:10.1038/s41467-023-39283-x)
Supplement: Supplementary file 1 — Supplementary Information [file 41467_2023_39283_MOESM1_ESM.pdf]

Supplementary Information

Reaction performance prediction with an extrapolative and interpretable graph model based on chemical knowledge

Shu-Wen Li[1], Li-Cheng Xu[1], Cheng Zhang[2], Shuo-Qing Zhang[1*], and Xin Hong[1,3,4*]

[1]Center of Chemistry for Frontier Technologies, Department of Chemistry, State Key Laboratory of Clean Energy Utilization, Zhejiang University, Hangzhou 310027, China
[2]Department of Chemistry, University of Science and Technology of China
[3]Beijing National Laboratory for Molecular Sciences, Zhongguancun North First Street NO. 2, Beijing 100190, PR China
[4]Key Laboratory of Precise Synthesis of Functional Molecules of Zhejiang Province, School of Science, Westlake University, 18 Shilongshan Road, Hangzhou 310024, Zhejiang Province, China
Email: hxchem@zju.edu.cn, angellasty@zju.edu.cn

Table of Contents

1. Technical details of machine learning 1
1.1 Details of tested descriptors and machine learning algorithms 1
1.1.1 Details of tested descriptors 1
1.1.2 Details of tested machine learning algorithms 2
1.2 Details of molecular graph training 3
1.2.1 Details of baseline molecular graph 3
1.2.2 Details of GCN model 4
1.3 Details of hyperparameter optimization for the tested machine learning models 5
1.4 Details of machine learning predictions on external experimental test set 6
2. Benchmark of the theoretical levels for geometry optimization and electron density calculation 8
2.1 Evaluation of the methods for geometry optimization 8
2.2 Evaluation of the methods for electron density calculation 10
3. Results of machine learning predictions 39
3.1 Results of the yield regression performances of Baseline MG-GCN 39
3.2 Results of the yield regression performances of SEMG-GCN 40
3.3 Results of the yield regression performances of Baseline MG-MIGNN 41
3.4 Results of the yield regression performances of SEMG-MIGNN 42
3.5 Results of the enantioselectivity regression performances of Baseline MG-GCN 43
3.6 Results of the enantioselectivity regression performances of SEMG-GCN 44
3.7 Results of the enantioselectivity regression performances of Baseline MG-MIGNN 45
3.8 Results of the enantioselectivity regression performances of SEMG-MIGNN 46
3.9 Results of other tested descriptors 47
3.10 Evaluation of structural sensitivity of the SEMG-MIGNN model 49
3.11 Learning curves of SEMG-MIGNN 51
3.12 Details of the machine learning modelling of the external experimental tests 52
3.13 Results of enantioselectivity regression performances on asymmetric hydrogenation of olefins ... 54
4. Comparison between SEMG-MIGNN and other SOTA models 55
4.1 Details of the tested SOTA models 55
4.2 Yield prediction in C–N cross coupling reaction 56
4.3 Enantioselectivity prediction in asymmetric *N,S*-acetal formation 60
5. Results of experiment 64
5.1 Experimental results of 11 new acids 64
5.2 HPLC Spectra 65
6. Data and code availability 71
Supplementary references 72

1. Technical details of machine learning

1.1 Details of tested descriptors and machine learning algorithms

1.1.1 Details of tested descriptors

In order to understand the baseline performances of machine learning modelling in a range of prediction tasks, we tested the predictive performances using widely applied molecular descriptors. The tested molecular descriptors include One-Hot, RDKit descriptors, Morgan Fingerprint, and Atom-centered Symmetry Functions. The tested molecular descriptors, as well as the corresponding generation parameters and package, are shown in Supplementary Table 1. All the related scripts for molecular descriptor generation are available in our GitHub project (https://github.com/Shuwen-Li/SEMG-MIGNN).

Supplementary Table 1. Type and generation package of tested molecular descriptors.

Molecular Descriptor	Parameters	Generation Package
One-Hot(OH, 1D)	default	Scikit-learn[4]
ML descriptor in RDKit[1](RDKit, 2D)	default	RDKit[1]
Morgan Fingerprint[2] (MF, 2D)	radius = 2, nBits = 2048, useChirality = True	RDKit[1]
Atom-centered Symmetry Functions[5] (ACSFs, 3D)	rcut = 6.0, g2_params = [[1, 1], [1, 2], [1, 3]],<br>g4_params = [[1, 1, 1], [1, 2, 1], [1, 1, -1], [1, 2, -1]]	DScribe[3]

1.1.2 Details of tested machine learning algorithms

A series of widely used machine learning algorithms were tested for the baseline model trainings, including AdaBoost[6], Bagging Regression[7], Decision Trees[8], Extra-Trees[9], Gradient Boosting[10], k-Nearest Neighbors Regression[11], Kernel Ridge Regression[12], Linear Support Vector Regression[13], Random Forest Regression[14], Ridge[15], Support Vector Regression[13], XGBoost[16], and Neural Network[17]. The model trainings were performed using scikit-learn[4] and xgboost python packages[18]. The parameters of each tested algorithm are included in Supplementary Table 2. All the related scripts for model training are available in our GitHub project (https://github.com/Shuwen-Li/SEMG-MIGNN). The details of hyperparameter setting ensembles and access of these algorithms are shown in Supplementary Table 2. For the parameters not shown in Supplementary Table 2, the default settings were used.

Supplementary Table 2. Hyperparameters of the tested machine learning algorithms for model training.

Model	Modules and parameters
AdaBoost[6] (Ada)	sklearn.ensemble.AdaBoostRegressor(base_estimator=sklearn.ensemble.ExtraTreesRegressor(n_jobs=60), n_estimators=10, learning_rate=1.0, loss='linear', random_state=None)
Bagging[7] (BG)	sklearn.ensemble.BaggingRegressor(base_estimator=None, n_estimators=100, max_samples=100, max_features=1.0, bootstrap=True, bootstrap_features=False, oob_score=False, warm_start=False, n_jobs=60, random_state=None, verbose=0)
Decision Tree[8] (DT)	sklearn.tree.DecisionTreeRegressor(criterion='squared_error', splitter='best', max_depth=None, min_samples_split=2, min_samples_leaf=1, min_weight_fraction_leaf=0.0, max_features=None, random_state=None, max_leaf_nodes=None, min_impurity_decrease=0.0, ccp_alpha=0.0)
Extra-Trees[9] (ET)	sklearn.ensemble.ExtraTreesRegressor(n_estimators=60, criterion='squared_error', max_depth=None, min_samples_split=2, min_samples_leaf=1, min_weight_fraction_leaf=0.0, max_features='auto', max_leaf_nodes=None, min_impurity_decrease=0.0, bootstrap=False, oob_score=False, n_jobs=60, random_state=None, verbose=0, warm_start=False, ccp_alpha=0.0, max_samples=None)
Gradient Boosting[10] (GB)	sklearn.ensemble.GradientBoostingRegressor(loss='ls', learning_rate=0.1, n_estimators=100, subsample=1.0, criterion='friedman_mse', min_samples_split=2, min_samples_leaf=1, min_weight_fraction_leaf=0.0, max_depth=4, min_impurity_decrease=0.0, init=None, random_state=None, max_features=None, alpha=0.9, verbose=0, max_leaf_nodes=None, warm_start=False, validation_fraction=0.1, n_iter_no_change=None, tol=0.0001, ccp_alpha=0.0)
k-Nearest Neighbors Regression[11] (KNR)	sklearn.neighbors.NearestNeighbors(n_neighbors=10,weights='uniform',algorithm='auto',leaf_size=30,p=2,metric='minkowski',metric_params=None,n_jobs=None)
KernelRidge[12] (KRR)	sklearn.kernel_ridge.KernelRidge(alpha=1, kernel='linear', gamma=None, degree=3, coef0=1, kernel_params=None)
Linear Support Vector Regression[13] (LSVR)	sklearn.svm.LinearSVR(epsilon=0.0,tol=0.0001,C=1.0,loss='epsilon_insensitive',fit_intercept=True,intercept_scaling=1.0,dual=True,verbose=0,random_state=None,max_iter=1000)
RandomForest[14] (RF)	sklearn.ensemble.RandomForestRegressor(n_estimators=100,criterion='mae',max_depth=None,min_samples_split=2,min_samples_leaf=1,min_weight_fraction_leaf=0.0,max_features='auto',max_leaf_nodes=None,min_impurity_decrease=0.0,bootstrap=True,oob_score=False,n_jobs=60,random_state=None,verbose=0,warm_start=False,ccp_alpha=0.0,max_samples=None)
Ridge[15]	sklearn.linear_model.Ridge(alpha=.5,fit_intercept=True,copy_X=True,max_iter=None,tol=0.001,)
Support Vector Regression[13] (SVR)	sklearn.svm.SVR(kernel='rbf', degree=3, gamma='scale', coef0=0.0, tol=0.001, C=1.0, epsilon=0.1, shrinking=True, cache_size=200, verbose=False, max_iter=- 1)
XGBoost[16] (XGB)	xgboost.XGBRegressor(base_score=0.5, booster='gbtree', colsample_bylevel=1, colsample_bynode=1, colsample_bytree=1, gamma=0, gpu_id=-1, importance_type='gain', interaction_constraints='', learning_rate=0.3, max_delta_step=0, max_depth=10, min_child_weight=1, missing=np.nan, monotone_constraints='()', n_estimators=60, num_parallel_tree=1, random_state=0, reg_alpha=0, reg_lambda=1, scale_pos_weight=1, subsample=1, tree_method='exact', validate_parameters=1, verbosity=None)
NeuralNetwork[17] (NN)	sklearn.neural_network.MLPRegressor(hidden_layer_sizes=(100,100), activation='relu', solver='adam', alpha=0.0001, batch_size='auto', learning_rate='constant', learning_rate_init=0.001, power_t=0.5, max_iter=200, shuffle=True, random_state=None, tol=0.0001, verbose=False, warm_start=False, momentum=0.9, nesterovs_momentum=True, early_stopping=False, validation_fraction=0.1, beta_1=0.9, beta_2=0.999, epsilon=1e-08, n_iter_no_change=10, max_fun=15000)

1.2 Details of molecular graph training

1.2.1 Details of baseline molecular graph

The baseline molecular graphs were generated by dgl[19]. The nodes of molecular graph included seven types of information: atom type, atomic number, acceptor, donor, aromatic, hybridization and the number of hydrogens. The edge feature is bond distance. Details of node and edge information are shown in Supplementary Table 3.

Supplementary Table 3. Description of baseline molecular graph.

Feature	Description	Dimension
Atom type	H, C, N, O, F… (One-hot)	7
Atomic number	Number of protons (Integer)	1
Acceptor	Accepts electrons (Binary)	1
Donor	Donates electrons (Binary)	1
Aromatic	In an aromatic system (Binary)	1
Hybridization	sp, sp^2, sp^3 (One-Hot)	3
Number of Hydrogens	(Integer)	1
Bond Distance	(Float)	1

1.2.2 Details of GCN model

For each molecule, the molecular graph was generated. Subsequently, the baseline molecular graphs were processed by the GCN layer using dgl. The processed molecular graphs were concatenated together and passed through the sum/mean/dense layers to predict the reaction performance. Detailed workflow of the GCN model is shown in Supplementary Figure 1.

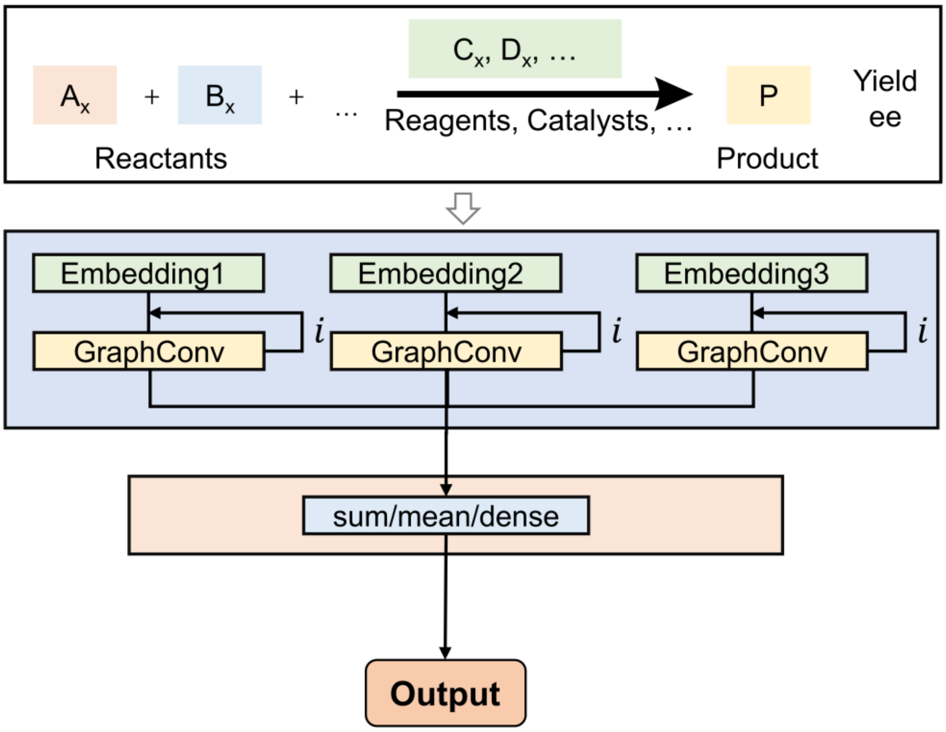

Supplementary Figure 1. Workflow of the GCN model (Graph Convolutional Network). For each molecule, the corresponding molecular graph was generated. Subsequently, the molecular graphs were processed by the GCN layer (Graph Convolutional Network). The processed molecular graphs were then concatenated together and passed through the sum/mean/dense layers to predict the reaction performance.

1.3 Details of hyperparameter optimization for the tested machine learning models

For the tested encoding methods and machine learning algorithms, we carefully performed the hyperparameter optimization by grid search method, in order to identify the optimal hyperparameter settings. Supplementary Table 4 provides the details of the hyperparameter optimization, and the optimization results are summarized in Supplementary Table 13 and Supplementary Table 14. The optimized hyperparameters were used in the subsequent comparisons of model performances.

Supplementary Table 4. Evaluated hyperparameters of the machine learning algorithms for model training. MIGNN means Molecular Interaction Graph Neural Network.

Model	Candidate parameters	Access
AdaBoost (Ada)	n_estimators: [50, 100, 150, 200];	scikit-learn
Bagging (BG)	n_estimators: [10, 20, 30, 40]	scikit-learn
Decision Tree (DT)	max_depth: [None, 10, 20, 30]	scikit-learn
Extra-Trees (ET)	n_estimators: [100, 200, 300, 400]; max_depth: [None, 10, 20, 30]	scikit-learn
Gradient Boosting (GB)	n_estimators: [50, 100, 150, 200]; max_depth: [3, 4, 5]	scikit-learn
k-Nearest Neighbors Regression (KNR)	n_neighbors: [5, 10, 15, 20]	scikit-learn
KernelRidge (KRR)	gamma: [None, 0.01, 0.001, 0.0001]	scikit-learn
Linear Support Vector Regression (LSVR)	epsilon: [0.0, 0.1, 0.2, 0.3]	scikit-learn
RandomForest (RF)	n_estimators: [100, 200, 300, 400]; max_depth: [None, 10, 20, 30];	scikit-learn
Ridge	alpha: [0.5, 1.0, 1.5]	scikit-learn
Support Vector Regression (SVR)	kernel: ['rbf', 'linear', 'poly']; gamma: ['scale', 'auto']	scikit-learn
XGBoost (XGB)	max_depth: [None, 10, 20, 30]	xgboost
NeuralNetwork (NN)	Hidden_layer_sizes:[(100,), (200,), (100,100,)]	scikit-learn
GCN	Convolution layer=[1, 2, 3], Multi graph=[mean, sum, max], output=[mean, sum, max]	dgl
MIGNN	linear_depth=[0,1,2,3,4,5,6,7,8,9,10], hidden_size=[8,16,32,64,128,256], atom_attention=[0,1,2], inter_attention=[0,1,2], end_attention=[0,1,2], fc_size=[32,64,128,256], final_act=['sigmoid',’none’]	tensorflow

1.4 Details of machine learning predictions on external experimental test set

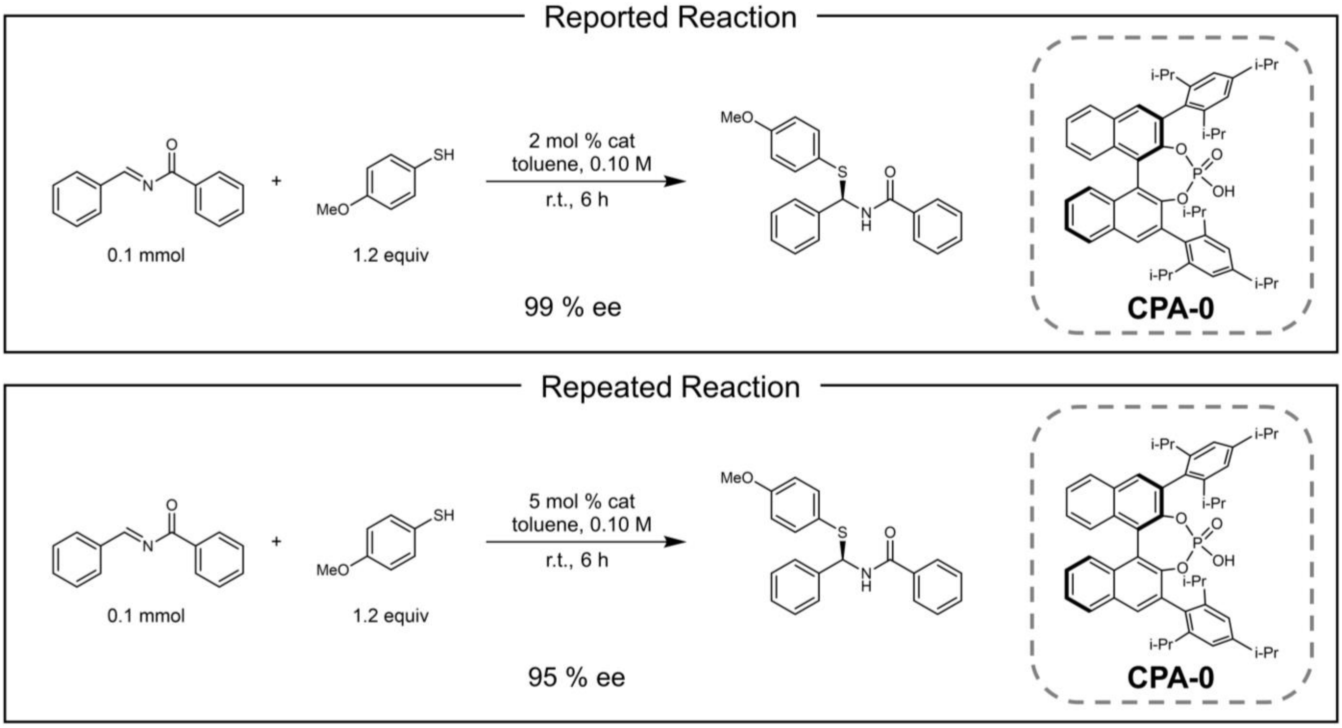

Supplementary Figure 2. Repeated result of chiral phosphoric acid-catalyzed thiol addition to *N*-acyl imine. The reported enantioselectivity under CPA-0 is 99% from Denmark's work[20]. Our repeated experiment gave an enantioselectivity of 95% ee under the same CPA catalyst, which may due to trace amount of inseparable Lewis acid.

To test the extrapolative abilities of the machine learning models, we performed a series of external experimental tests. These tests used the chiral phosphoric acid-catalyzed thiol addition to *N*-acylimine under 11 new chiral phosphoric acid catalysts (experimental details are provided in section *5. Results of experiment*). To ensure the reliability of our experimental data, we first repeated one transformation using an identical chiral phosphoric acid **CPA-0** from Denmark's report[20]. This transformation was reported to have a 99% ee, which corresponds to a 3.13 kcal mol^{-1} free energy difference. Despite extensive efforts, our repeated experiment consistently gave an enantioselectivity of 95% ee, 2.17 kcal mol^{-1} (Supplementary Figure 2).

We believe this mitigation of enantioselectivity raised from the influence of the racemic background reaction, probably catalyzed by the trace amount of inseparable Lewis acid. During the experimental explorations, we noticed that the imine addition is very fast, and we tried our best to eliminate the influence of background reaction. We have tried various means of purification and more stringent reaction setups, such as new glassware for each transformation, but we still cannot completely avoid the reduction of enantioselectivity. In order to make reasonable predictions using the Denmark's statistics-trained machine learning model, we applied a scaling factor of Denmark's statistics as a reasonable compromise. The mechanistic reasoning of scaling factor is elaborated as follows:

Without the background reaction, $\Delta\Delta G^{\ddagger} = -RT\ln\frac{1+ee}{1-ee} = -RT[\ln(1+ee) - \ln(1-ee)]$ (1)

With the background reaction, $\Delta\Delta G^{\ddagger}_{w/bg} = -RT[\ln(1+ee_{w/bg}) - \ln(1-ee_{w/bg})]$ (2)

Applying second-order Taylor expansion:

$$\frac{\Delta\Delta G^{\ddagger}}{\Delta\Delta G^{\ddagger}_{w/bg}} = \frac{ee - \frac{1}{2}ee^{2} - \left(-ee - \frac{1}{2}ee^{2}\right)}{ee_{w/bg} - \frac{1}{2}{ee_{w/bg}}^{2} - \left(-ee_{w/bg} - \frac{1}{2}{ee_{w/bg}}^{2}\right)} = \frac{ee}{ee_{w/bg}}$$

$$= \frac{k_{pref\_tot} + k_{bg\_tot}}{k_{pref\_tot}} = 1 + \frac{k_{bg\_tot}}{k_{pref\_tot}} \quad (3)$$

Because the substrates were synthesized by the same reaction and subsequently purified, the content of the Lewis acid should be comparable, and the corresponding background reaction rate is approximately constant (k_{bg_tot}). In addition, since all the chiral catalysts are CPA, the total rates of the catalytic reactions (k_{pref_tot}) should also be approximately comparable (k_{pref_tot} is the total reaction rate under CPA catalysis, which does not require the enantioselectivity to be the same). Therefore, even there is a discrepancy in the actual enantioselectivity, we believe the $\frac{\Delta\Delta G^{\ddagger}}{\Delta\Delta G^{\ddagger}_{w/bg}}$ can be considered as a constant based on the above approximation. Based on the repeated experiments, we defined this value as 0.693 ($\Delta\Delta G_{\text{transferred}} = \Delta\Delta G_{\text{original}} \times 0.693$). All the enantioselectivity values (in kcal mol^{-1}) in Denmark's dataset were timed by 0.693, so that our repeating transformation has the identical target label as the transferred Denmark's dataset (2.17 = 3.13 × 0.693). With the transferred Denmark's dataset, we trained the machine learning models and make predictions for the 11 new CPA catalysts.

2. Benchmark of the theoretical levels for geometry optimization and electron density calculation

2.1 Evaluation of the methods for geometry optimization

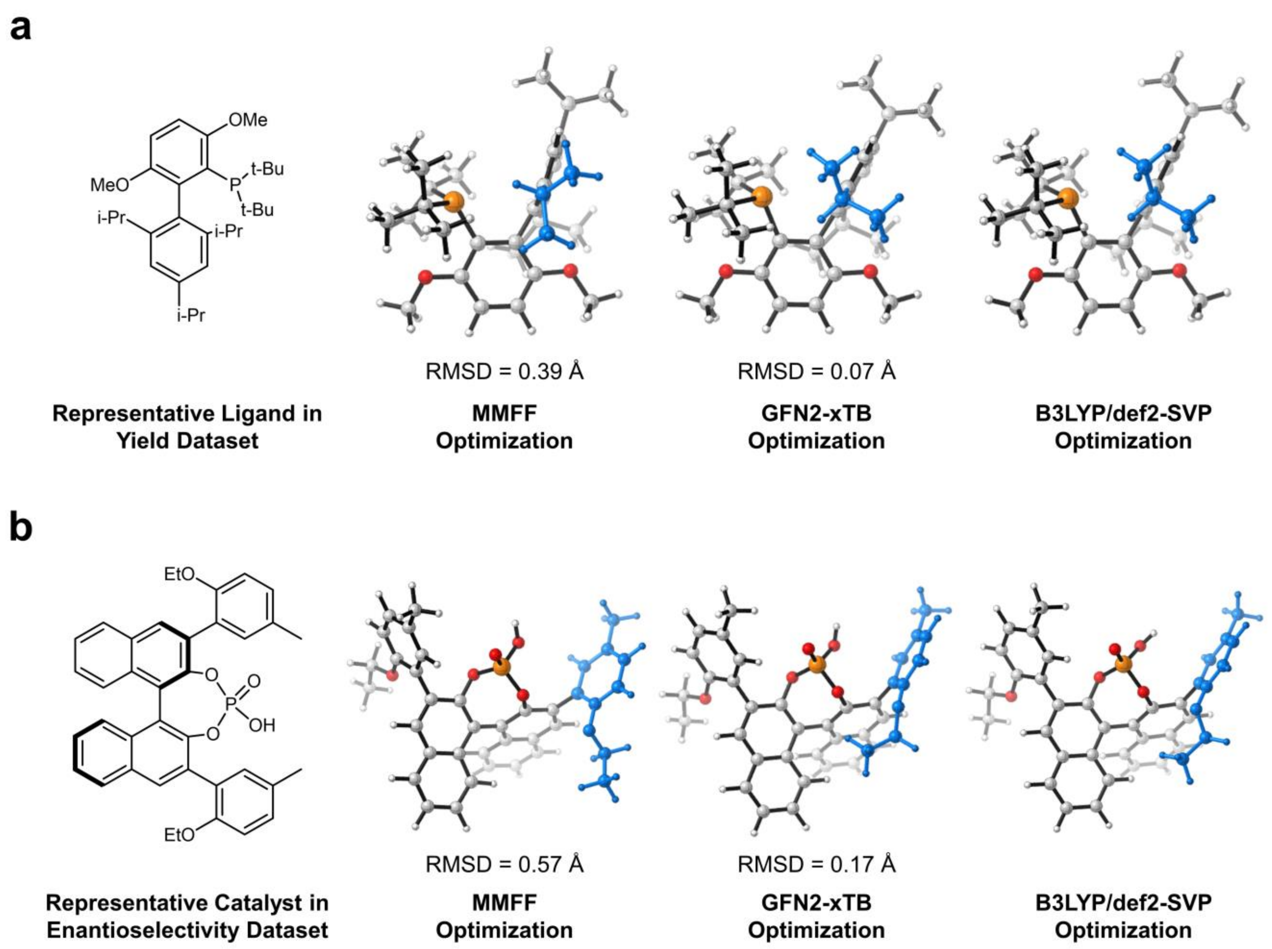

Supplementary Figure 3. Comparisons of the optimized geometries of representative molecules in yield and enantioselectivity datasets at various levels of theory. The blue atoms are the portions of the molecule with significant structural differences. a Using the B3LYP/def2-SVP optimized structure as the standard, the RMSDs (root-mean-square deviations) of the structures optimized by MMFF and GFN2-xTB levels of theory for a representative ligand in yield dataset. **b** Using the B3LYP/def2-SVP optimized structure as the standard, the RMSDs (root-mean-square deviations) of the structures optimized by MMFF and GFN2-xTB levels of theory for a representative catalyst in enantioselectivity dataset.

In order to find the suitable level for geometry optimization to obtain the steric encodings accurately and efficiently, we compared the geometries optimized by the MMFF, GFN2-xTB, and DFT (B3LYP/def2-SVP) methods, and representative results are shown in Supplementary Figure 3. Using the B3LYP/def2-SVP structures as reference, the root-mean-square deviation (RMSD) of the MMFF and GFN2-xTB were computed. It was shown that MMFF is not suitable for the geometry optimization of complex molecules, giving incorrect orientations for certain key substituents (such as the highlighted ones in Supplementary Figure 3) and yielding an unsatisfying level of RMSD. In comparison, GFN2-xTB significantly improved the accuracy of geometry optimization, achieving a level close to that of DFT optimization while still meeting our requirements for high-

throughput virtual screening. Therefore, we eventually chose GFN2-xTB level of theory for geometry optimization.

To ensure the reliability of the GFN2-xTB geometries in terms of modelling accuracy, we further compared the regression performances of SEMG-MIGNN models (Sterics- and Electronics-embedded Molecular Graph and Molecular Interaction Graph Neural Network) trained by GFN2-xTB (Supplementary Figure 4a) and B3LYP/def2-SVP (Supplementary Figure 4b) geometries. In both yield and enantioselectivity prediction tasks, the two models have comparable predictive abilities. These comparisons further supported that the selected GFN2-xTB level of theory can provide the required accuracy for geometry optimization and enable the desired machine learning modelling.

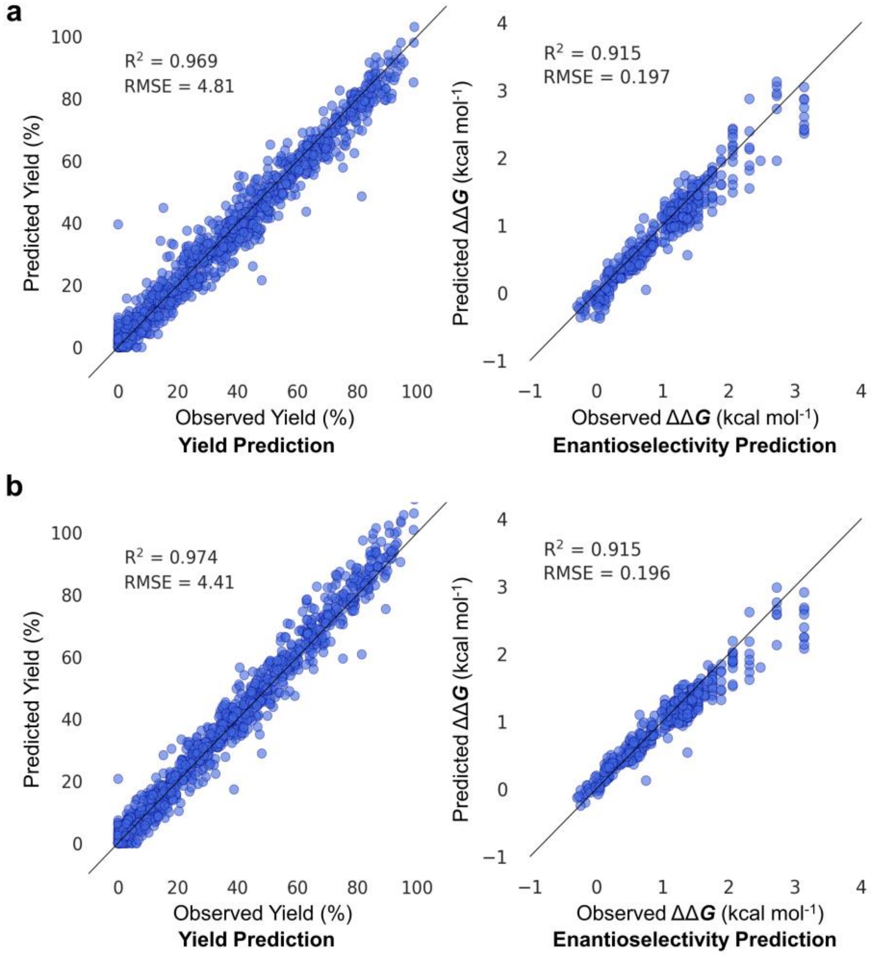

Supplementary Figure 4. Test set performances of the SEMG-MIGNN models (Sterics- and Electronics-embedded Molecular Graph and Molecular Interaction Graph Neural Network) trained by the geometries optimized at the GFN2-xTB level (a) and the B3LYP/def2-SVP level (b). The yield dataset is randomly split to 70% (training) and 30% (test). The enantioselectivity task is randomly split to 600 (training) and 475 (test) transformations.

2.2 Evaluation of the methods for electron density calculation

In order to find the suitable level for the calculation of electron density, we have evaluated the electron densities calculated by various methods. We ultimately selected the theoretical level of B3LYP/def2-SVP to obtain the electron densities and process the model trainings.

Based on the GFN2-xTB-optimized geometries, the accuracies of the computed electron densities were evaluated for thirty-five levels of theory including the variations of five functionals (LDA-VWN, B3LYP, M06-2X, ωB97X-D, PBE0) and seven basis sets (STO-3G, STO-6G, 3-21G, def2-SVP, 6-31G(d), 6-311+G**, def2-TZVPP). The evaluation process is elaborated in Supplementary Figure 5a. For a given molecule in the studied dataset, the electron densities of the same geometry were compared between two levels of theory: the reference level (B3LYP/def2-TZVPP) and the comparing level (the other thirty-four levels). The neighboring electron density of each atom was assessed to obtain a 7x7x7 tensor with the vdW diameter size. This creates a Nx7x7x7 tensor for the entire molecule, which was flattened into an one-dimensional vector. Subsequently, the Euclidean distances between the two vectors were calculated to provide the quantified evaluation of the change of electron densities.

The total of 97 molecules involved in the reactivity and enantioselectivity datasets were examined, and the average Euclidean distances of each level of theory are shown in Supplementary Figure 5b. This analysis identified four main levels of accuracies for the studied computational methods. It is worth noting that as the size of basis set increases, the calculation efficiency decreases significantly. Considering the trade-off between accuracy and efficiency, we have selected the level of B3LYP/def2-SVP (F5B4) for the electron density calculations.

To further verify the physical accuracy of the selected B3LYP/def2-SVP level, we compared the electrostatic potential surfaces, which is an important representation of the spatial distribution of the electron density. Supplementary Figure 6 to Supplementary Figure 12 shows the electrostatic potential surfaces of the 97 molecules involved in the yield and enantioselectivity datasets calculated by B3LYP/def2-SVP and B3LYP/def2-TZVPP (isovalue=0.0004); under the same scale, the changes between the two levels of theory are quite limited. These comparisons further demonstrate that the selected B3LYP/def2-SVP approach can provide physically accurate electron density.

In addition, we used the electron densities calculated by the B3LYP/def2-SVP and B3LYP/def2-TZVPP levels to train the SEMG-MIGNN models (Sterics- and Electronics-embedded Molecular Graph and Molecular Interaction Graph Neural Network) and compared the prediction performances. Supplementary Figure 13 shows the model performances trained by different electron density inputs. In both yield and enantioselectivity prediction tasks, further increasing the physical accuracy from def2-SVP level (Supplementary Figure 13a) to def2-TZVPP level (Supplementary Figure 13b) only led to limited improvement of regression performances (R2: 0.969 vs. 0.971 in yield task; 0.915 vs. 0.918 in enantioselectivity task). These additional evaluations supported that the selected B3LYP/def2-SVP level of theory can provide solid accuracy for the electron density and support the desired machine learning modelling.

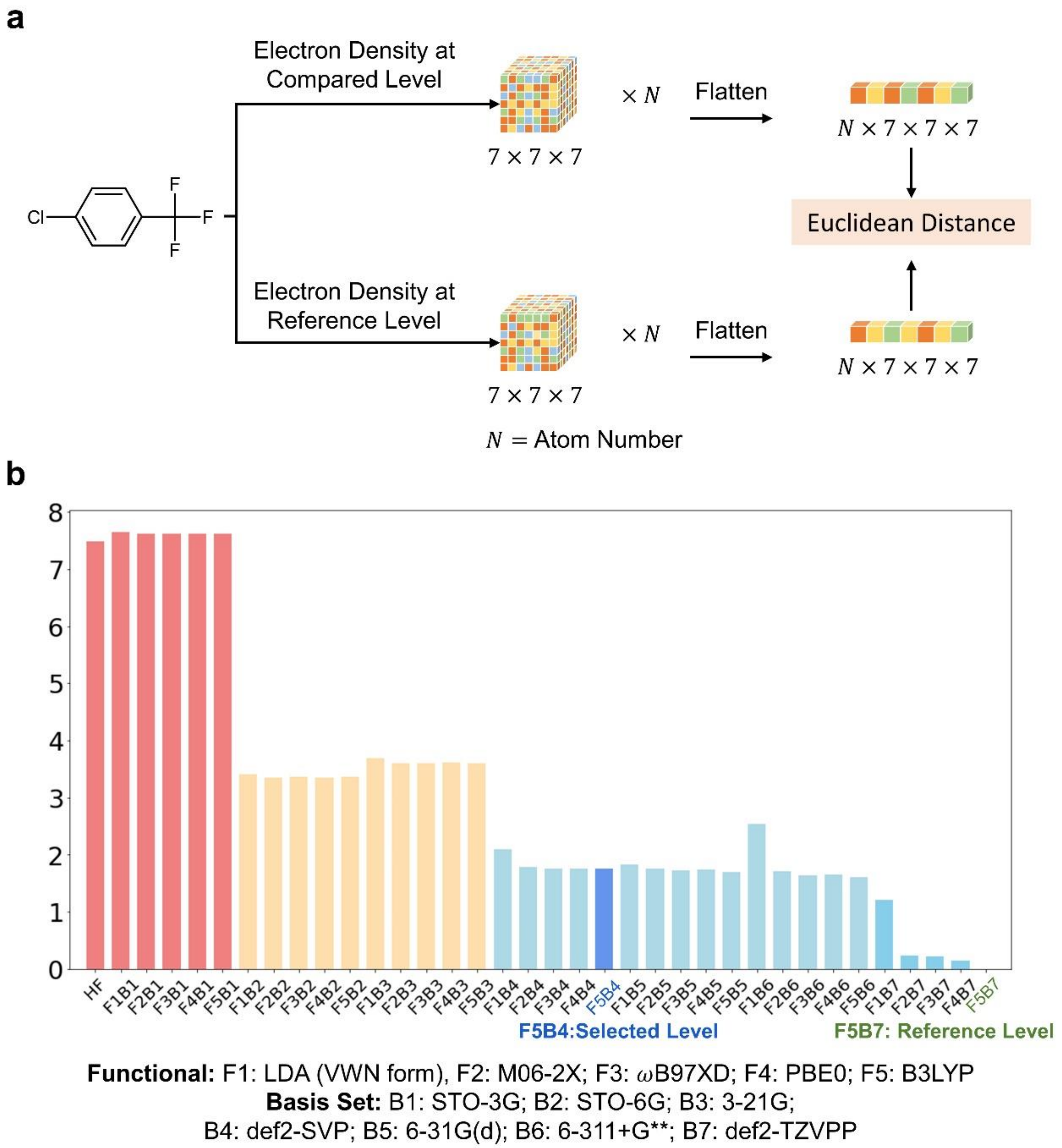

Supplementary Figure 5. Quantitative evaluation of the computed electron density at various theoretical levels. a Evaluation procedure of the Euclidean distance between the vectors of the computed electron densities. **b** Euclidean distances of the vectors generated by thirty-five theoretical levels.

Supplementary Table 5. Labelling of the 97 molecules for Supplementary Figure 6.

Entry	Label	SMILES
1	Additive-1	COC1=NOC(C(OCC)=O)=C1
2	Additive-2	C1(N(CC2=CC=CC=C2)CC3=CC=CC=C3)=NOC=C1
3	Additive-3	C12=C(C=CC=C2)ON=C1
4	Additive-4	CC1=C(C(OCC)=O)C=NO1
5	Additive-5	O=C(OC)C1=NOC(C2=CC=CS2)=C1
6	Additive-6	C1(C2=CC=CC=C2)=CC=NO1
7	Additive-7	O=C(OC)C1=NOC(C2=CC=CO2)=C1
8	Additive-8	CC1=CC(C(OCC)=O)=NO1
9	Additive-9	CC1=NOC(C(OCC)=O)=C1
10	Additive-10	CCOC(C1=NOC=C1)=O
11	Additive-11	C1(C2=CC=CC=C2)=CON=C1
12	Additive-12	CCOC(C1=CON=C1)=O
13	Additive-13	C12=CON=C1C=CC=C2
14	Additive-14	CC1=CC=NO1
15	Additive-15	C1(N(CC2=CC=CC=C2)CC3=CC=CC=C3)=CC=NO1
16	Additive-16	CC1=NOC=C1
17	Additive-17	C1(C2=CC=CC=C2)=NOC=C1
18	Additive-18	O=C(OC)C1=CC=NO1
19	Additive-19	FC(C=CC=C1F)=C1C2=CC=NO2
20	Additive-20	CC1=NOC(C2=CC=CC=C2)=C1
21	Additive-21	CC1=CC(N2C=CC=C2)=NO1
22	Additive-22	CC1=CC(C)=NO1
23	Base-1	CN1CCCN2C1=NCCC2
24	Base-2	CN(C)P(N(C)C)(N(C)C)=NP(N(C)C)(N(C)C)=NCC
25	Base-3	CC(C)(C)/N=C(N(C)C)/N(C)C
26	Aryl Halides-1	BrC1=CN=CC=C1
27	Aryl Halides-2	ClC1=CC=C(C(F)(F)F)C=C1
28	Aryl Halides-3	ClC1=NC=CC=C1
29	Aryl Halides-4	IC1=CC=C(OC)C=C1
30	Aryl Halides-5	IC1=NC=CC=C1
31	Aryl Halides-6	IC1=CC=C(C(F)(F)F)C=C1
32	Aryl Halides-7	ClC1=CN=CC=C1
33	Aryl Halides-8	IC1=CN=CC=C1

34	Aryl Halides-9	BrC1=CC=C(C(F)(F)F)C=C1
35	Aryl Halides-10	BrC1=CC=C(CC)C=C1
36	Aryl Halides-11	ClC1=CC=C(CC)C=C1
37	Aryl Halides-12	BrC1=NC=CC=C1
38	Aryl Halides-13	IC1=CC=C(CC)C=C1
39	Aryl Halides-14	BrC1=CC=C(OC)C=C1
40	Aryl Halides-15	ClC1=CC=C(OC)C=C1
41	Ligand-1	CC(C)C(C=C(C(C)C)C=C1C(C)C)=C1C2=C(P(C(C)(C)C)C(C)(C)C)C(OC)=CC=C2OC
42	Ligand-2	CC(C)C(C=C(C(C)C)C=C1C(C)C)=C1C2=C(P(C(C)(C)C)C(C)(C)C)C=CC=C2
43	Ligand-3	CC(C)C(C=C(C(C)C)C=C1C(C)C)=C1C2=C(P(C3CCCCC3)C4CCCCC4)C=CC=C2
44	Ligand-4	CC(C)C(C=C(C(C)C)C=C1C(C)C)=C1C2=C(P([C@@]3(C[C@@H]4C5)C[C@H](C4)C[C@H]5C3)[C@]6(C7)C[C@@H](C[C@@H]7C8)C[C@@H]8C6)C(OC)=CC=C2OC
45	CPA-1	O=P1(O)OC2=C(Br)C=C3C(C=CC=C3)=C2C4=C(O1)C(Br)=CC5=CC=CC=C54
46	CPA-2	O=P1(O)OC2=C(Br)C=C3C(CCCC3)=C2C4=C(O1)C(Br)=CC5=C4CCCC5
47	CPA-3	O=P1(O)OC2=C(C3=C(C(C)C)C=C(C(C)C)C=C3C(C)C)C=C4C(C=CC=C4)=C2C5=C(O1)[C@@]([C@@]6=C(C(C)C)C=C(C(C)C)C=C6C(C)C)=CC7=C5C=CC=C7
48	CPA-4	O=P1(O)OC2=C(C3=C(C(C)C)C=C(C4=CC=C(C(C)(C)C)C=C4)C=C3C(C)C)C=C5C(C=CC=C5)=[C@]2[C@]6=C(O1)C(C7=C(C(C)C)C=C(C8=CC=C(C(C)(C)C)C=C8)C=C7C(C)C)=CC9=C6C=CC=C9
49	CPA-5	O=P1(O)OC2=C(C3=C(C)C=C(C)C=C3C)C=C4C(C=CC=C4)=C2C5=C(O1)[C@@]([C@@]6=C(C)C=C(C)C=C6C)=CC7=C5C=CC=C7
50	CPA-6	O=P1(O)OC2=C(C3=C(C4=CC(C=CC=C5)=C5C=C4)C=CC=C3)C=C6C(CCCC6)=C2C7=C(O1)C(C8=CC=CC=C8C9=CC=C(C=CC=C%10)C%10=C9)=CC%11=C7CCCC%11
51	CPA-7	O=P1(O)OC2=C(C3=C(C4CCCCC4)C=C(C5CCCCC5)C=C3C6CCCCC6)C=C7C(C=CC=C7)=C2C8=C(O1)[C@@]([C@@]9=C(C%10CCCCC%10)C=C(C%11CCCCC%11)C=C9C%12CCCCC%12)=CC%13=C8C=CC=C%13
52	CPA-8	O=P1(O)OC2=C(C3=C(C=CC4=CC=CC(C=C5)=C46)C6=C5C=C3)C=C7C(C=CC=C7)=C2C8=C(O1)C(C9=CC=C(C=C%10)C%11=C9C=CC%12=CC=CC%10=C%11%12)=CC%13=C8C=CC=C%13
53	CPA-9	O=P1(O)OC2=C(C3=C(C=CC=C4)C4=C(C5=CC(C=CC=C6)=C6C=C5)C7=C3C=CC=C7)C=C8C(C=CC=C8)=[C@]2[C@]9=C(O1)C(C%10=C(C=CC=C%11)C%11=C(C%12=CC=C(C=CC=C%13)C%13=C%12)C%14=C%10C=CC=C%14)=CC%15=C9C=CC=C%15

54	CPA-10	O=P1(O)OC2=C(C3=C(C=CC=C4)C4=CC5=C3C=CC=C5)C=C6C(C=CC=C6)=C2C7=C(O1)[C@@]([C@@]8=C(C=CC=C9)C9=CC%10=C8C=CC=C%10)=CC%11=C7C=CC=C%11
55	CPA-11	O=P1(O)OC2=C(C3=C(F)C=C(OC)C=C3F)C=C4C(C=CC=C4)=[C@]2[C@]5=C(O1)C(C6=C(F)C=C(OC)C=C6F)=CC7=C5C=CC=C7
56	CPA-12	O=P1(O)OC2=C(C3=C(OC(F)(F)F)C=CC=C3)C=C4C(C=CC=C4)=C2C5=C(O1)C(C6=CC=CC=C6OC(F)(F)F)=CC7=C5C=CC=C7
57	CPA-13	O=P1(O)OC2=C(C3=C(OC)C=CC=C3OC)C=C4C(C=CC=C4)=C2C5=C(O1)[C@@]([C@@]6=C(OC)C=CC=C6OC)=CC7=C5C=CC=C7
58	CPA-14	O=P1(O)OC2=C(C3=C(OCC)C=CC(C)=C3)C=C4C(C=CC=C4)=C2C5=C(O1)C(C6=CC(C)=CC=C6OCC)=CC7=C5C=CC=C7
59	CPA-15	O=P1(O)OC2=C(C3=CC(C(C)(C)C)=CC(C(C)(C)C)=C3)C=C4C(C=CC=C4)=C2C5=C(O1)C(C6=CC(C(C)(C)C)=CC(C(C)(C)C)=C6)=CC7=C5C=CC=C7
60	CPA-16	O=P1(O)OC2=C(C3=CC(C(C)(C)C)=CC(C(C)(C)C)=C3)C=C4C(CCCC4)=C2C5=C(O1)C(C6=CC(C(C)(C)C)=CC(C(C)(C)C)=C6)=CC7=C5CCCC7
61	CPA-17	O=P1(O)OC2=C(C3=CC(C(F)(F)F)=CC(C(F)(F)F)=C3)C=C4C(C=CC=C4)=C2C5=C(O1)C(C6=CC(C(F)(F)F)=CC(C(F)(F)F)=C6)=CC7=C5C=CC=C7
62	CPA-18	O=P1(O)OC2=C(C3=CC(C(F)(F)F)=CC(C(F)(F)F)=C3)C=C4C(CCCC4)=C2C5=C(O1)C(C6=CC(C(F)(F)F)=CC(C(F)(F)F)=C6)=CC7=C5CCCC7
63	CPA-19	O=P1(O)OC2=C(C3=CC(C)=C(OC(C)C)C(C)=C3)C=C4C(C=CC=C4)=C2C5=C(O1)C(C6=CC(C)=C(OC(C)C)C(C)=C6)=CC7=C5C=CC=C7
64	CPA-20	O=P1(O)OC2=C(C3=CC(C4=C(C)C=C(C)C=C4C)=CC(C5=C(C)C=C(C)C=C5C)=C3)C=C6C(CCCC6)=C2C7=C(O1)C(C8=CC(C9=C(C)C=C(C)C=C9C)=CC(C%10=C(C)C=C(C)C=C%10C)=C8)=CC%11=C7CCCC%11
65	CPA-21	O=P1(O)OC2=C(C3=CC(C4=CC(C(F)(F)F)=CC(C(F)(F)F)=C4)=CC(C5=CC(C(F)(F)F)=CC(C(F)(F)F)=C5)=C3)C=C6C(C=CC=C6)=C2C7=C(O1)C(C8=CC(C9=CC(C(F)(F)F)=CC(C(F)(F)F)=C9)=CC(C%10=CC(C(F)(F)F)=CC(C(F)(F)F)=C%10)=C8)=CC%11=C7C=CC=C%11
66	CPA-22	O=P1(O)OC2=C(C3=CC(C4=CC(C=CC=C5)=C5C=C4)=CC=C3)C=C6C(C=CC=C6)=C2C7=C(O1)C(C8=CC=CC(C9=CC=C(C=CC=C%10)C%10=C9)=C8)=CC%11=C7C=CC=C%11
67	CPA-23	O=P1(O)OC2=C(C3=CC(C4=CC=C(OC)C=C4)=CC(C5=CC=C(OC)C=C5)=C3)C=C6C(C=CC=C6)=C2C7=C(O1)C(C8=CC(C9=CC=C(OC)C=C9)=CC(C%10=CC=C(OC)C=C%10)=C8)=CC%11=C7C=CC=C%11
68	CPA-24	O=P1(O)OC2=C(C3=CC(COC)=CC=C3)C=C4C(C=CC=C4)=C2C5=C(O1)C(C6=CC=

		CC(COC)=C6)=CC7=C5C=CC=C7
69	CPA-25	O=P1(O)OC2=C(C3=CC(COC)=CC=C3)C=C4C(CC CC4)=C2C5=C(O1)C(C6=CC=CC(COC)=C6)=CC7=C5CCCC7
70	CPA-26	O=P1(O)OC2=C(C3=CC=C(C(C)(C)C)C=C3)C=C4C (C=CC=C4)=C2C5=C(O1)C(C6=CC=C(C(C) (C)C)C=C6)=CC7=C5C=CC=C7
71	CPA-27	O=P1(O)OC2=C(C3=CC=C(C)C=C3)C=C4C(C=CC =C4)=C2C5=C(O1)C(C6=CC=C(C)C=C6)=CC7=C5C=CC=C7
72	CPA-28	O=P1(O)OC2=C(C3=CC=C(C4=CC(C(F)(F)F)=CC (C(F)(F)F)=C4)C=C3)C=C5C(C=CC=C5)=C2C6=C(O1) C(C7=CC=C(C8=CC(C(F)(F)F)=CC(C(F)(F)F)=C8)C=C7)=CC9=C6C=CC=C9
73	CPA-29	O=P1(O)OC2=C(C3=CC=C(C4=CC=C(C=CC=C5) C5=C4)C=C3)C=C6C(C=CC=C6)=C2C7=C(O1)C(C8 =CC=C(C9=CC(C=CC=C%10)=C%10C=C9)C=C8)=CC%11=C7C=CC=C%11
74	CPA-30	O=P1(O)OC2=C(C3=CC=C(C4CCCCC4)C=C3)C= C5C(CCCC5)=C2C6=C(O1)C(C7=CC=C (C8CCCCC8)C=C7)=CC9=C6CCCC9
75	CPA-31	O=P1(O)OC2=C(C3=CC=C(OC)C=C3)C=C4C(C=C C=C4)=C2C5=C(O1)C(C6=CC=C(OC)C=C6)=CC7=C5C=CC=C7
76	CPA-32	O=P1(O)OC2=C(C3=CC=C(S(F)(F)(F)(F)F)C=C3)C= C4C(C=CC=C4)=C2C5=C(O1)C(C6=CC=C(S(F) (F)(F)(F)F)C=C6)=CC7=C5C=CC=C7
77	CPA-33	O=P1(O)OC2=C(C3=CC=CC=C3)C=C4C(C=CC= C4)=C2C5=C(O1)C(C6=CC=CC=C6)=CC7=C5C=CC=C7
78	CPA-34	O=P1(O)OC2=C(CC)C=C3C(CCCC3)=C2 C4=C(O1)C(CC)=CC5=C4CCCC5
79	CPA-35	O=P1(O)OC2=C(CC3=C(C=CC=C4)C4=CC5=C3 C=CC=C5)C=C6C(C=CC=C6)=C2C7=C(O1)C(CC8 =C(C=CC=C9)C9=CC%10=C8C=CC=C%10)=CC%11=C7C=CC=C%11
80	CPA-36	O=P1(O)OC2=C(CC3=CC(C(F)(F)F)=CC(C(F)(F)F)= C3)C=C4C(CCCC4)=C2C5=C(O1)C(CC6=CC(C (F)(F)F)=CC(C(F)(F)F)=C6)=CC7=C5CCCC7
81	CPA-37	O=P1(O)OC2=C(CC3=CC=C(C(F)(F)F)C=C3C(F)(F)F)C=C4C(C=CC=C4)=C2C5=C(O1)C(CC6=C(C (F)(F)F)C=C(C(F)(F)F)C=C6)=CC7=C5C=CC=C7
82	CPA-38	O=P1(O)OC2=C(CC3=CC=C(OC)C=C3)C=C4C (CCCC4)=C2C5=C(O1)C(CC6=CC=C(OC)C=C6)=CC7=C5CCCC7
83	CPA-39	O=P1(O)OC2=C([Si](C3=CC=C(C(C)(C)C)C=C3) (C4=CC=C(C(C)(C)C)C=C4)C5=CC=C(C(C)(C)C) C=C5)C=C6C(CCCC6)=C2C7=C(O1)C([Si](C8= CC=C(C(C)(C)C)C=C8)(C9=CC=C(C(C)(C)C)C=C9) C%10=CC=C(C(C)(C)C)C=C%10)=CC%11=C7CCCC%11
84	CPA-40	O=P1(O)OC2=C([Si](C3=CC=CC=C3)(C)C4=CC= CC=C4)C=C5C(C=CC=C5)=C2C6=C(O1)C([Si](C7 =CC=CC=C7)(C8=CC=CC=C8)C)=CC9=C6C=CC=C9
85	CPA-41	O=P1(O)OC2=C([Si](C3=CC=CC=C3)(C4=CC=C C=C4)C5=CC=CC=C5)C=C6C(C=CC=C6)=C2C7 =C(O1)C([Si](C8=CC=CC=C8)(C9=CC=CC=C9)

		C%10=CC=CC=C%10)=CC%11=C7C=CC=C%11
86	CPA-42	O=P1(O)OC2=C([Si](C3=CC=CC=C3)(C4=CC=C C=C4)C5=CC=CC=C5)C=C6C(CCCC6)=C2C7=C (O1)C([Si](C8=CC=CC=C8)(C9=CC=CC=C9)C %10=CC=CC=C%10)=CC%11=C7CCCC%11
87	CPA-43	O=P1(O)OC2=[C@]([C@]3=C(Cl)C=C(Cl)C=C3Cl)C =C4C(CCCC4)=[C@]2[C@]5=C(O1)C(C6= C(Cl)C=C(Cl)C=C6Cl)=CC7=C5CCCC7
88	Imine-1	O=C(C1=CC=CC=C1)/N=C/C2=CC=C(C(F)(F)F)C=C2
89	Imine-2	O=C(C1=CC=CC=C1)/N=C/C2=CC=C(Cl)C=C2Cl
90	Imine-3	O=C(C1=CC=CC=C1)/N=C/C2=CC=C(OC)C=C2
91	Imine-4	O=C(C1=CC=CC=C1)/N=C/C2=CC=CC3=C2C=CC=C3
92	Imine-5	O=C(C1=CC=CC=C1)/N=C/C2=CC=CC=C2
93	Thiol-1	CCS
94	Thiol-2	SC1=CC=C(OC)C=C1
95	Thiol-3	SC1=CC=CC=C1
96	Thiol-4	SC1=CC=CC=C1C
97	Thiol-5	SC1CCCCC1

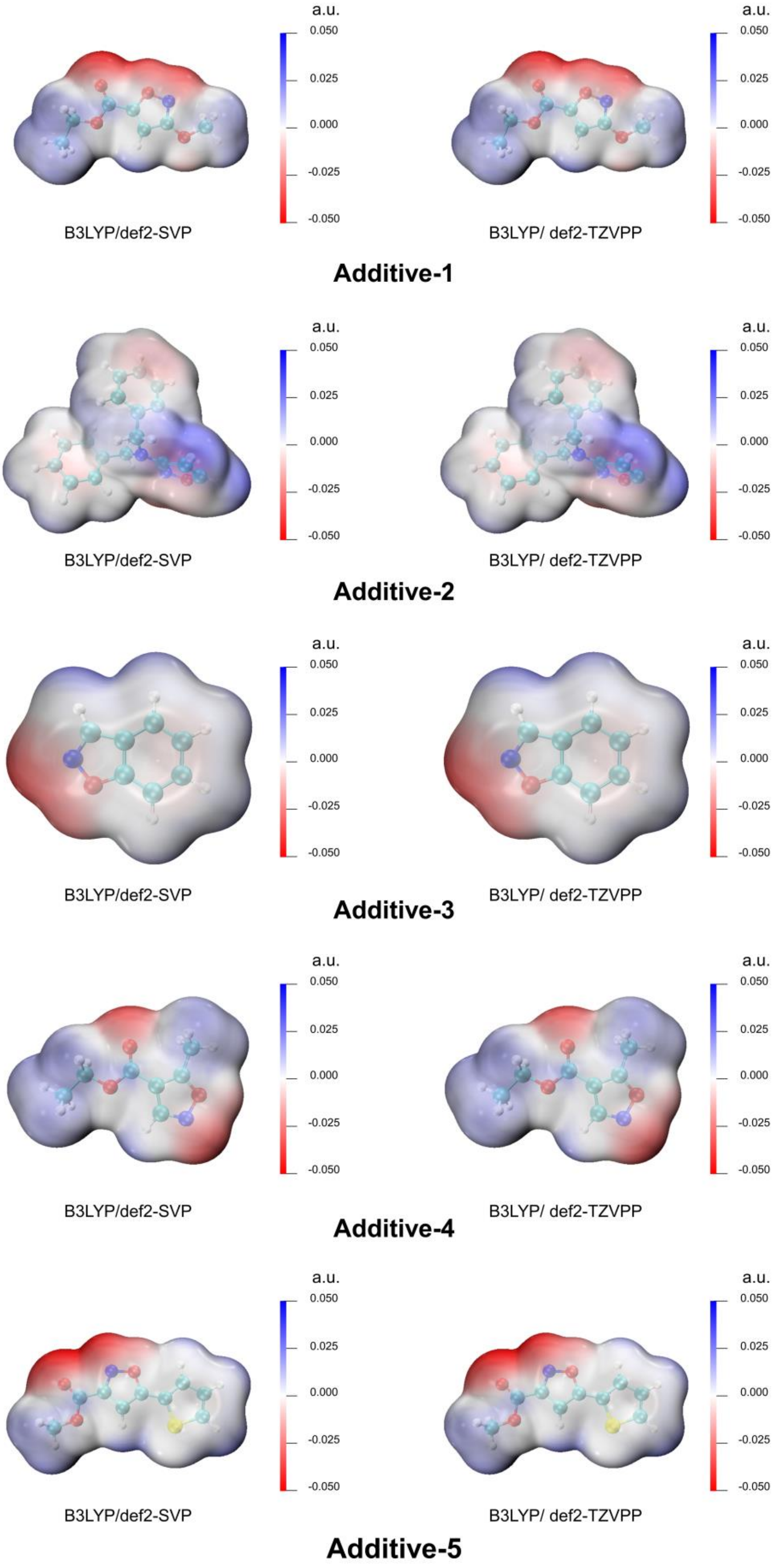

Supplementary Figure 6A. Electrostatic potential surfaces of the additives involved in the yield dataset calculated at the B3LYP/def2-SVP level and the B3LYP/ def2-TZVPP level. "a.u." means atomic units.

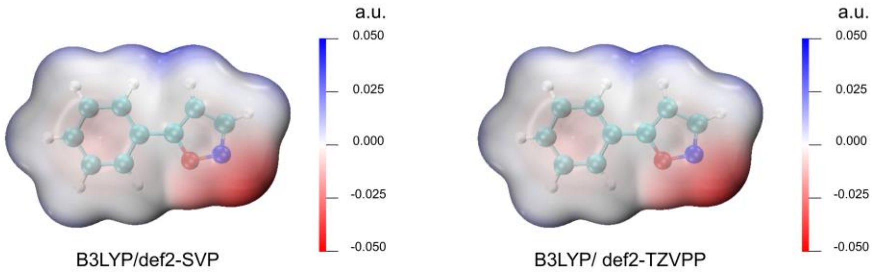

Additive-6

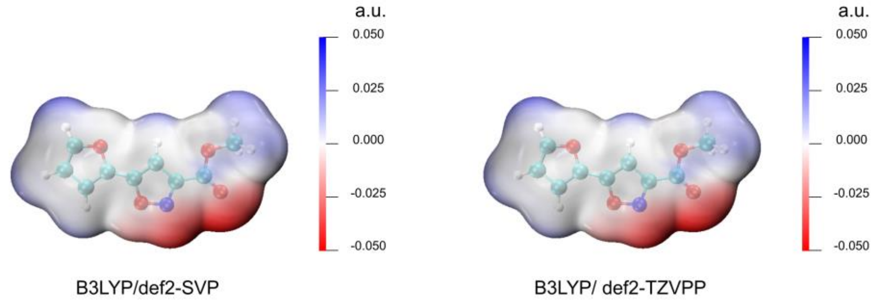

Additive-7

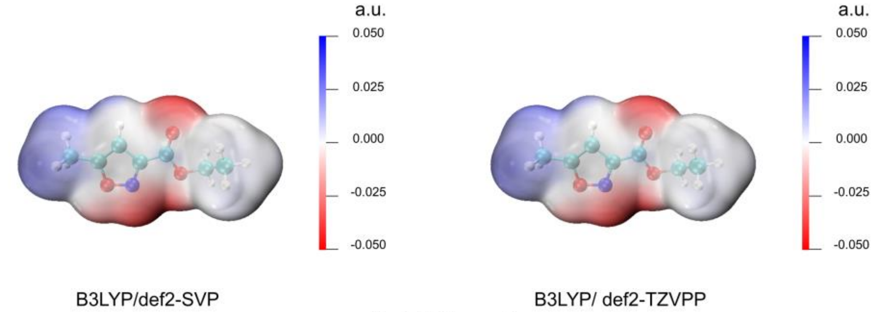

Additive-8

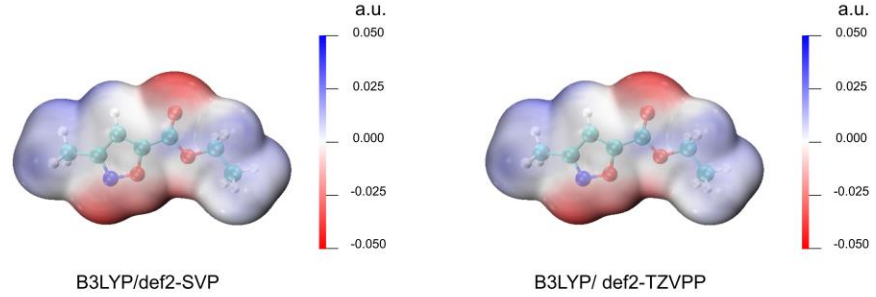

Additive-9

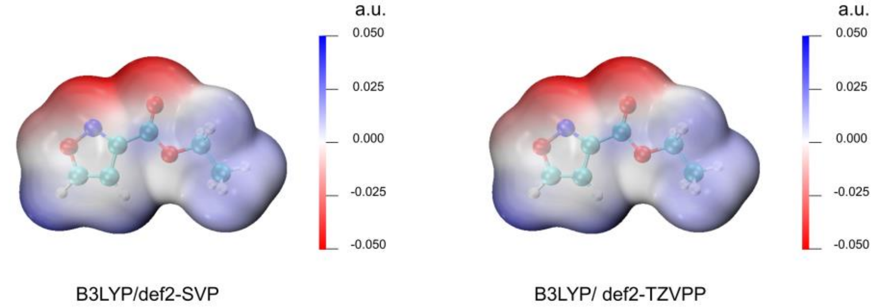

Additive-10

Supplementary Figure 6B. Electrostatic potential surfaces of the additives involved in the yield dataset calculated at the B3LYP/def2-SVP level and the B3LYP/ def2-TZVPP level. "a.u." means atomic units.

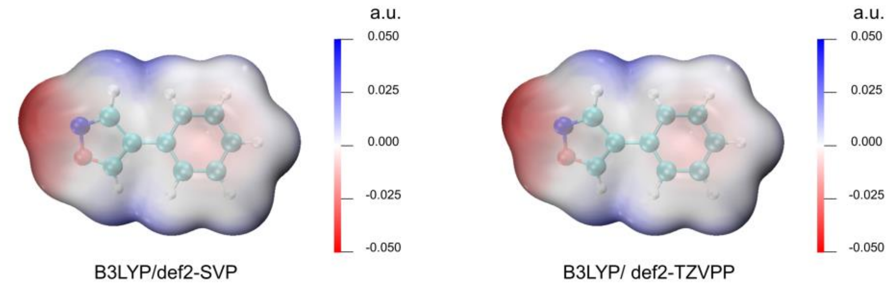

Additive-11

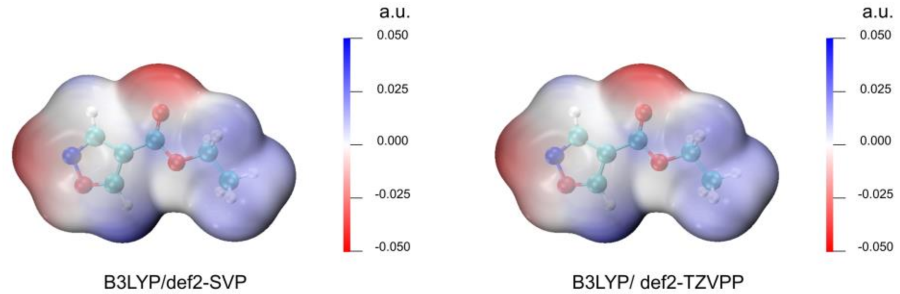

Additive-12

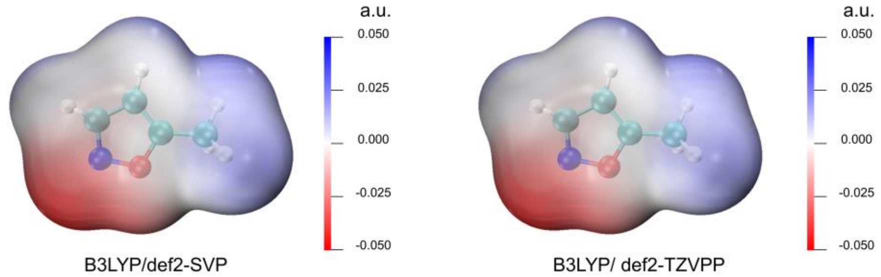

Additive-13

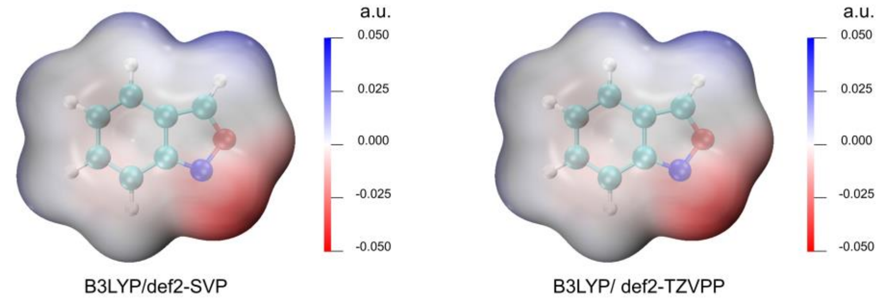

Additive-14

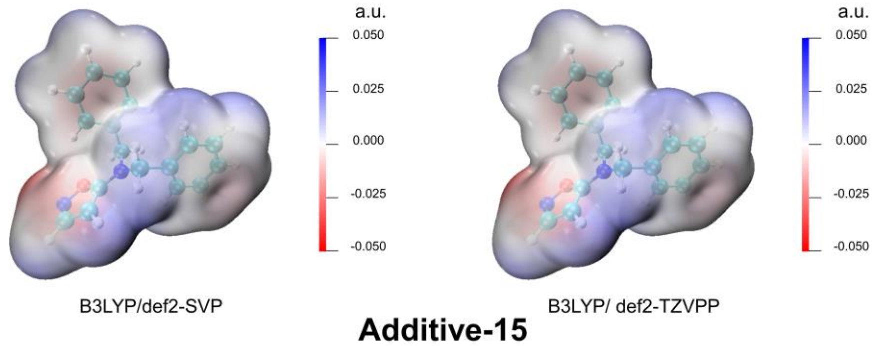

Additive-15

Supplementary Figure 6C. Electrostatic potential surfaces of the additives involved in the yield dataset calculated at the B3LYP/def2-SVP level and the B3LYP/ def2-TZVPP level. "a.u." means atomic units.

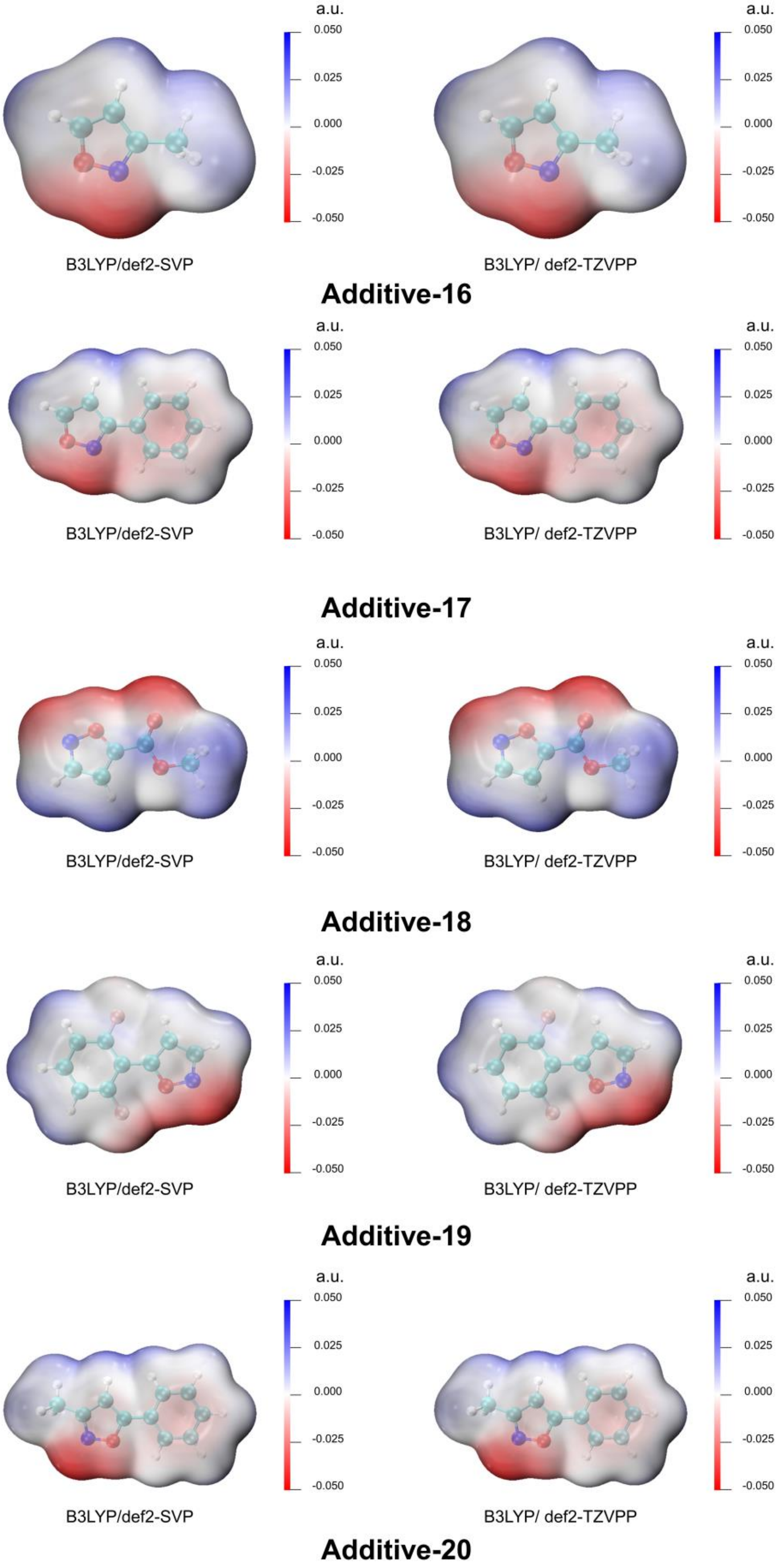

Supplementary Figure 6D. Electrostatic potential surfaces of the additives involved in the yield dataset calculated at the B3LYP/def2-SVP level and the B3LYP/ def2-TZVPP level. "a.u." means atomic units.

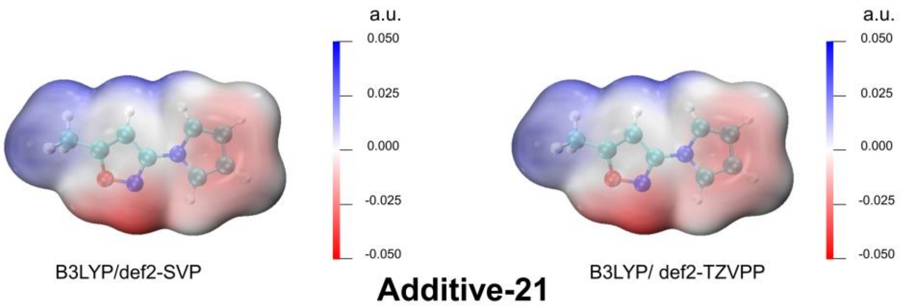

Additive-21

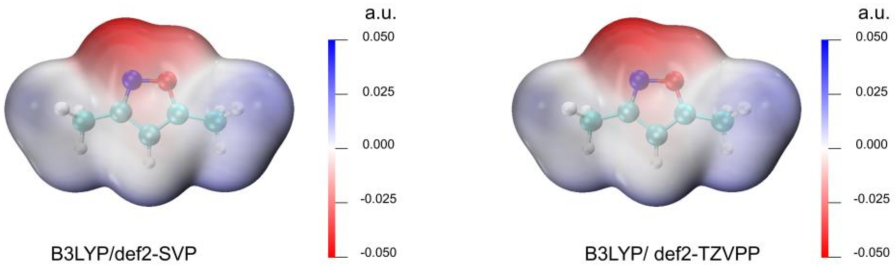

Additive-22

Supplementary Figure 6E. Electrostatic potential surfaces of the additives involved in the yield dataset calculated at the B3LYP/def2-SVP level and the B3LYP/ def2-TZVPP level. "a.u." means atomic units.

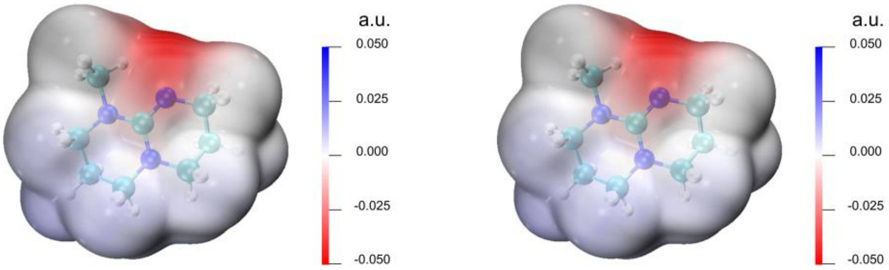

B3LYP/def2-SVP B3LYP/ def2-TZVPP

Base-1

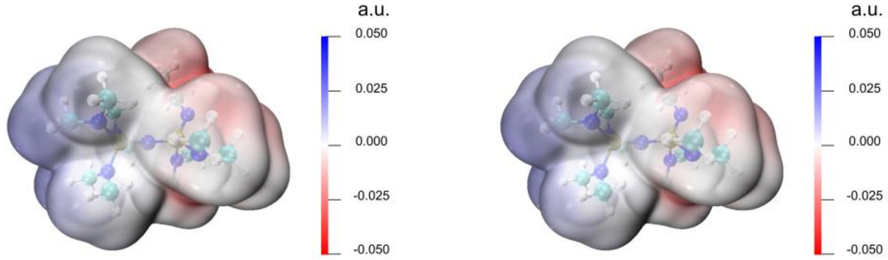

B3LYP/def2-SVP B3LYP/ def2-TZVPP

Base-2

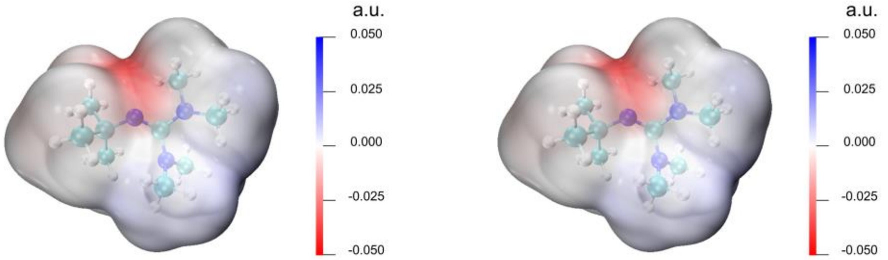

B3LYP/def2-SVP B3LYP/ def2-TZVPP

Base-3

Supplementary Figure 7. Electrostatic potential surfaces of the bases involved in the yield dataset calculated at the B3LYP/def2-SVP level and the B3LYP/ def2-TZVPP level. "a.u." means atomic units.

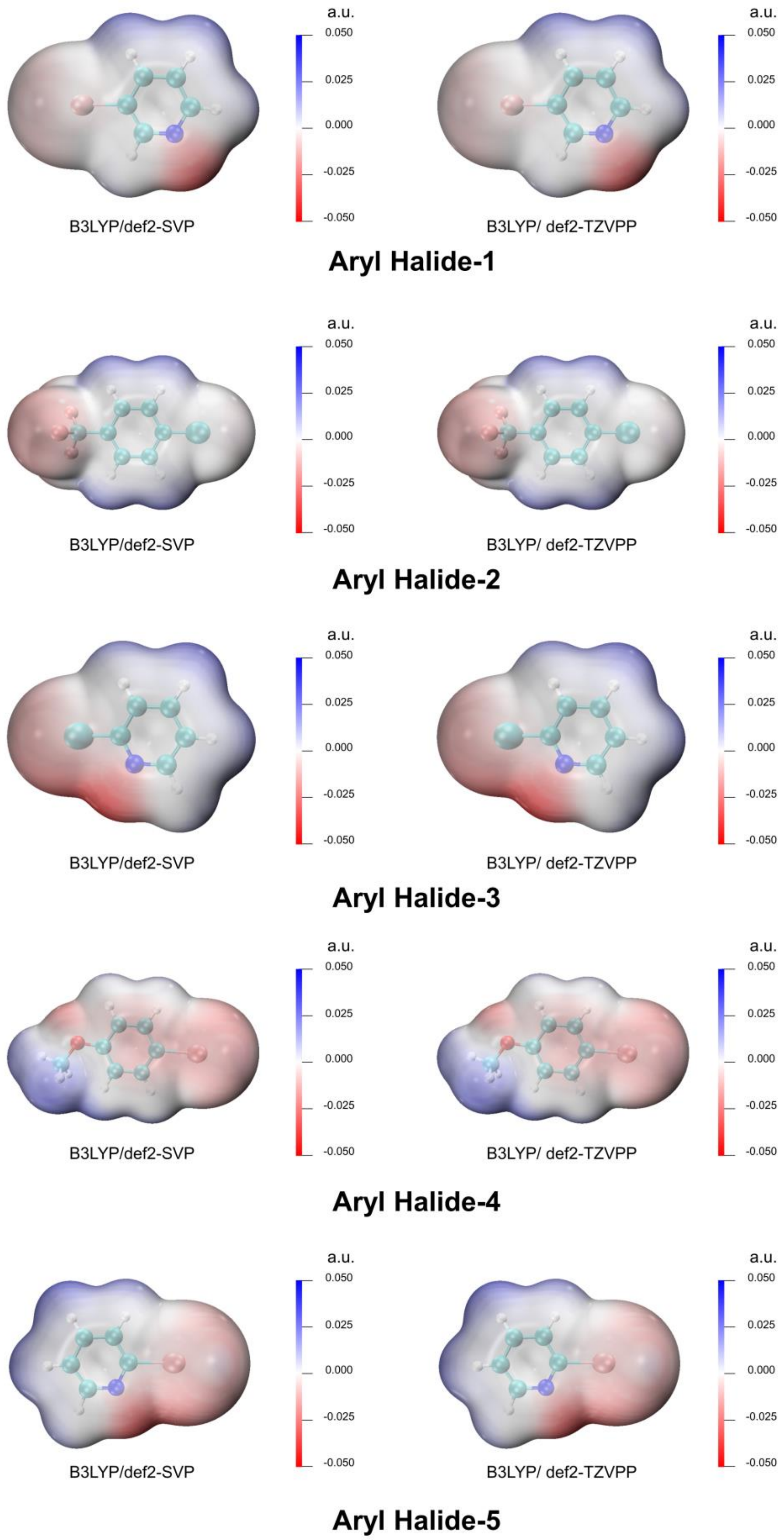

Supplementary Figure 8A. Electrostatic potential surfaces of the aryl halides involved in the yield dataset calculated at the B3LYP/def2-SVP level and the B3LYP/ def2-TZVPP level. "a.u." means atomic units.

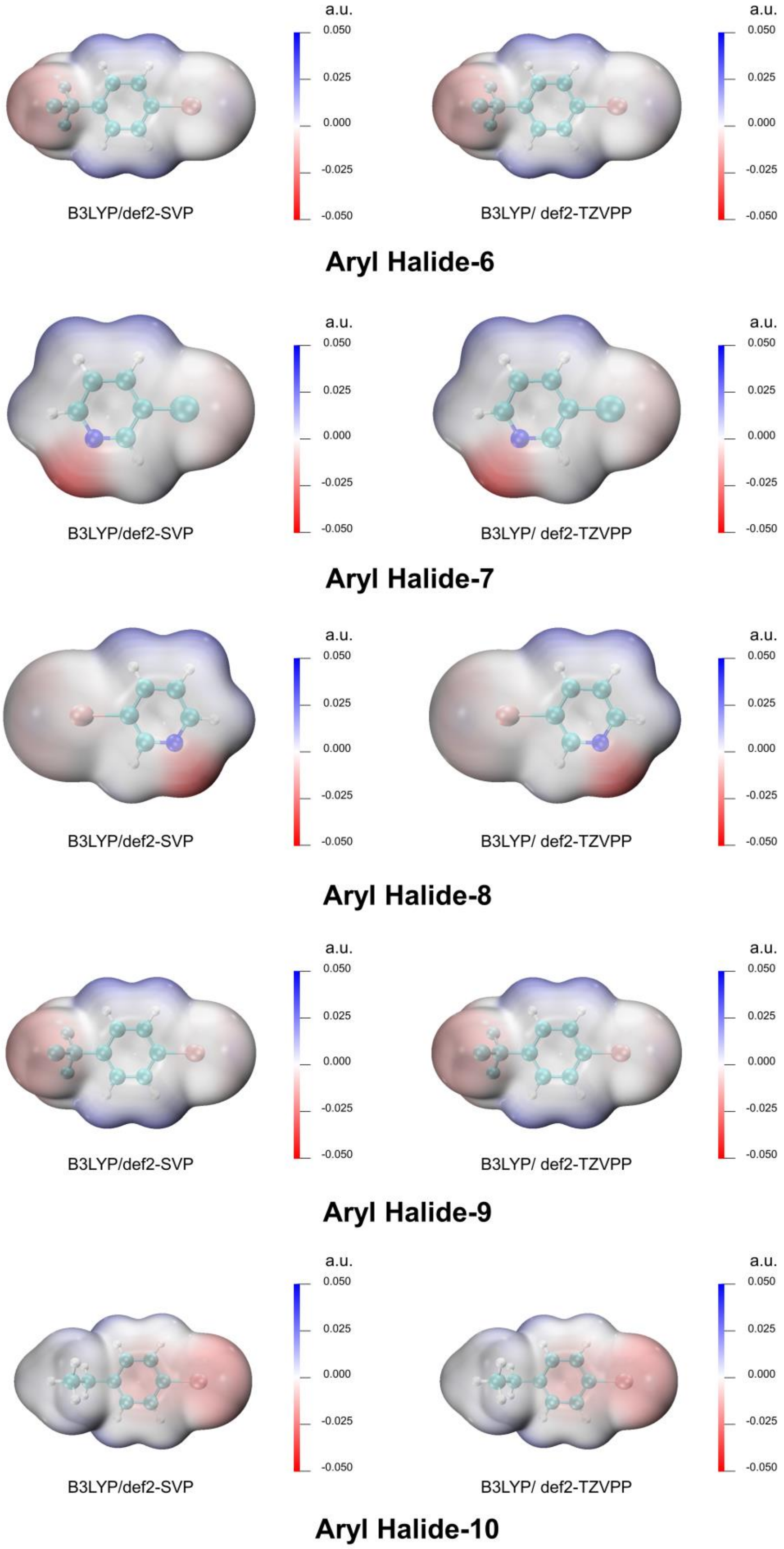

Supplementary Figure 8B. Electrostatic potential surfaces of the aryl halides involved in the yield dataset calculated at the B3LYP/def2-SVP level and the B3LYP/ def2-TZVPP level. "a.u." means atomic units.

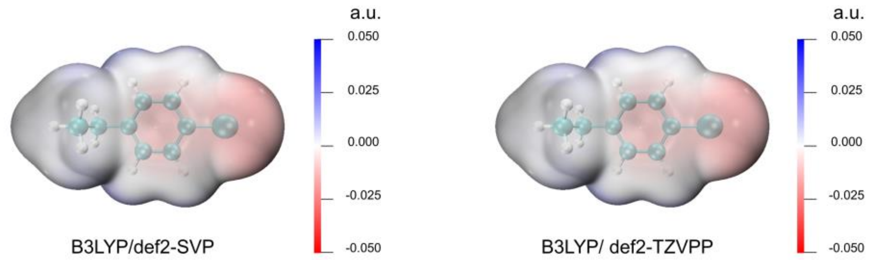

Aryl Halide-11

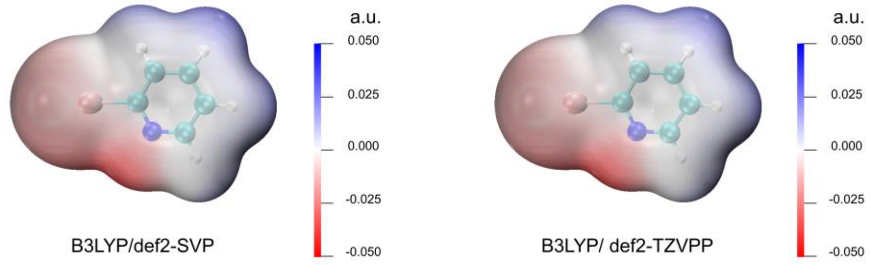

Aryl Halide-12

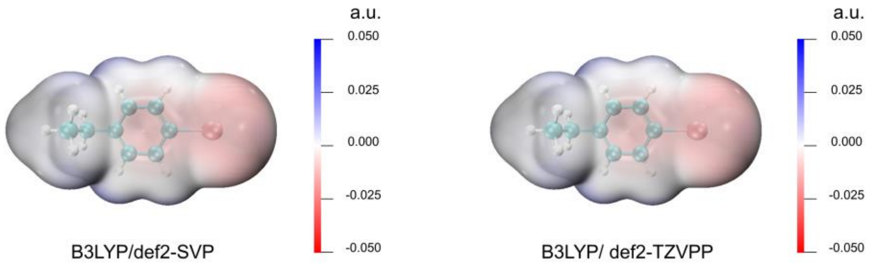

Aryl Halide-13

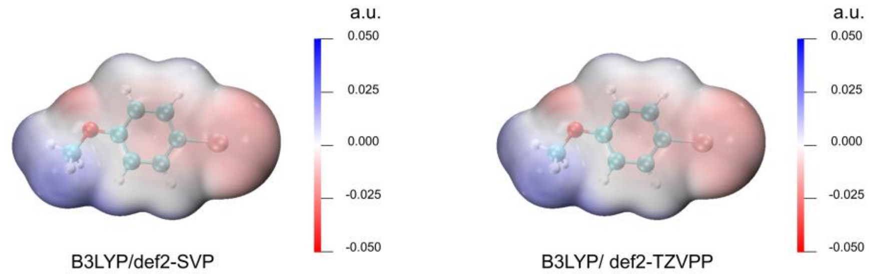

Aryl Halide-14

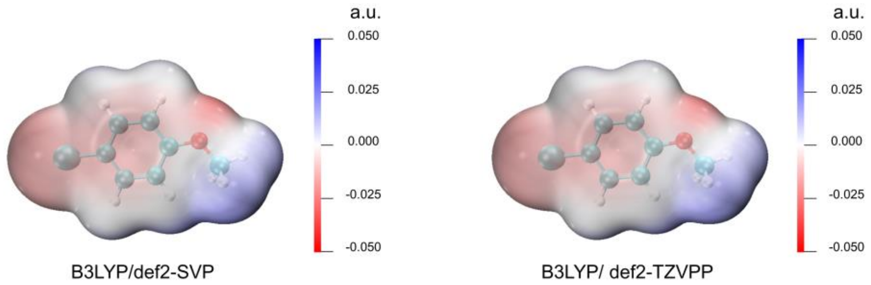

Aryl Halide-15

Supplementary Figure 8C. Electrostatic potential surfaces of the aryl halides involved in the yield dataset calculated at the B3LYP/def2-SVP level and the B3LYP/ def2-TZVPP level. "a.u." means atomic units.

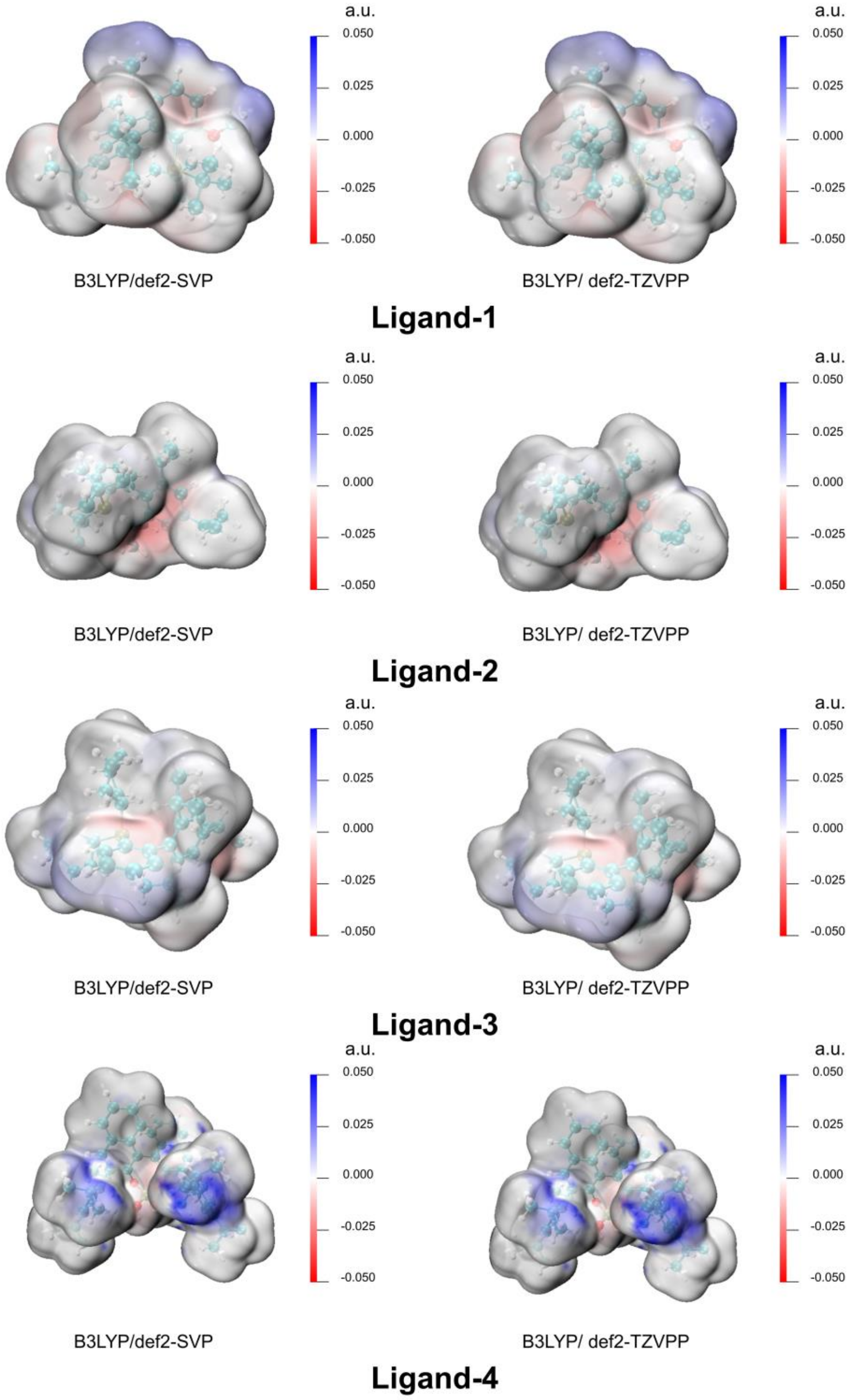

Supplementary Figure 9. Electrostatic potential surfaces of the ligands involved in the yield dataset calculated at the B3LYP/def2-SVP level and the B3LYP/ def2-TZVPP level. "a.u." means atomic units.

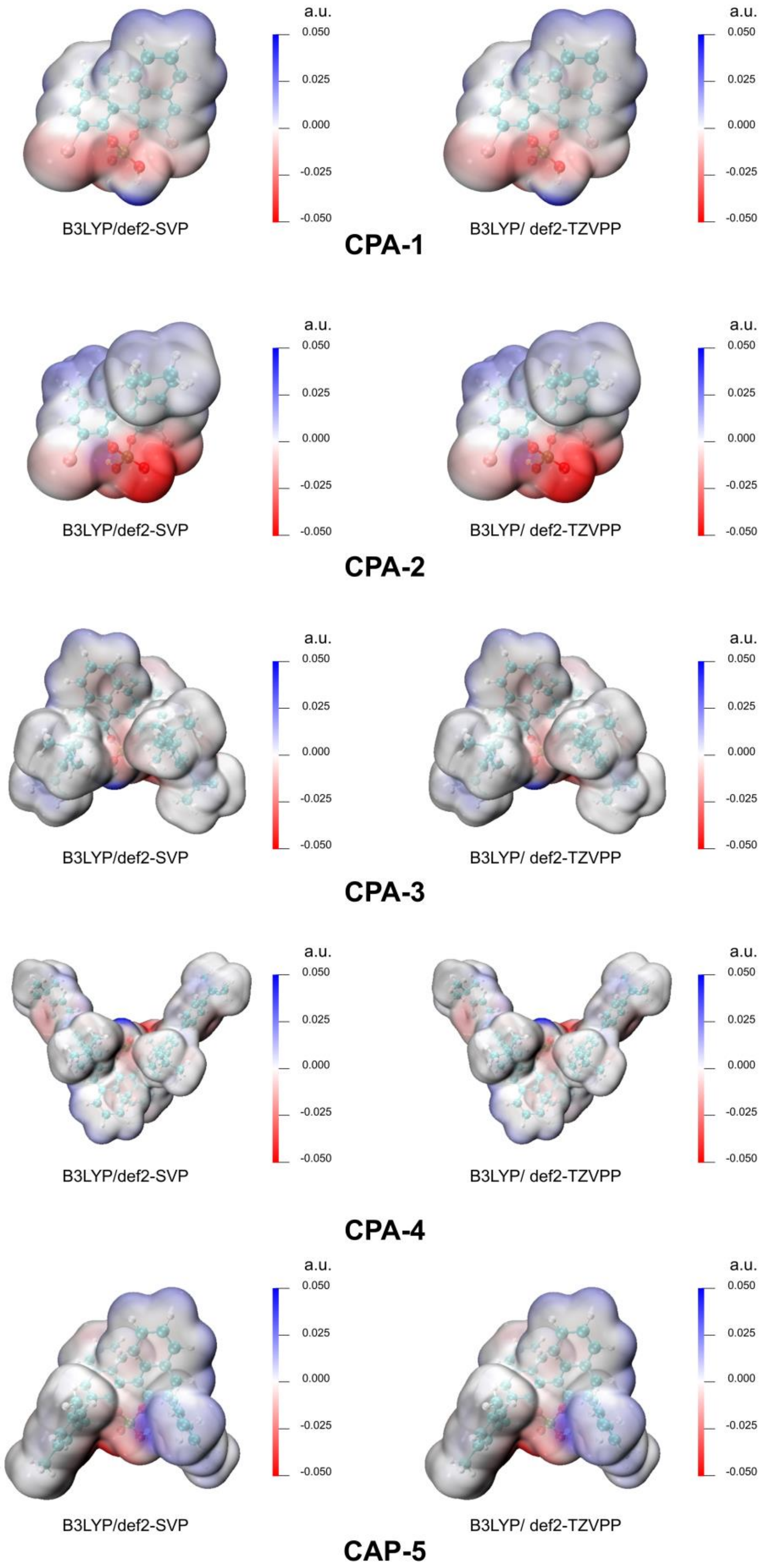

Supplementary Figure 10A. Electrostatic potential surfaces of the CPAs in the enantioselectivity dataset calculated at the B3LYP/def2-SVP level and the B3LYP/ def2-TZVPP level. "a.u." means atomic units.

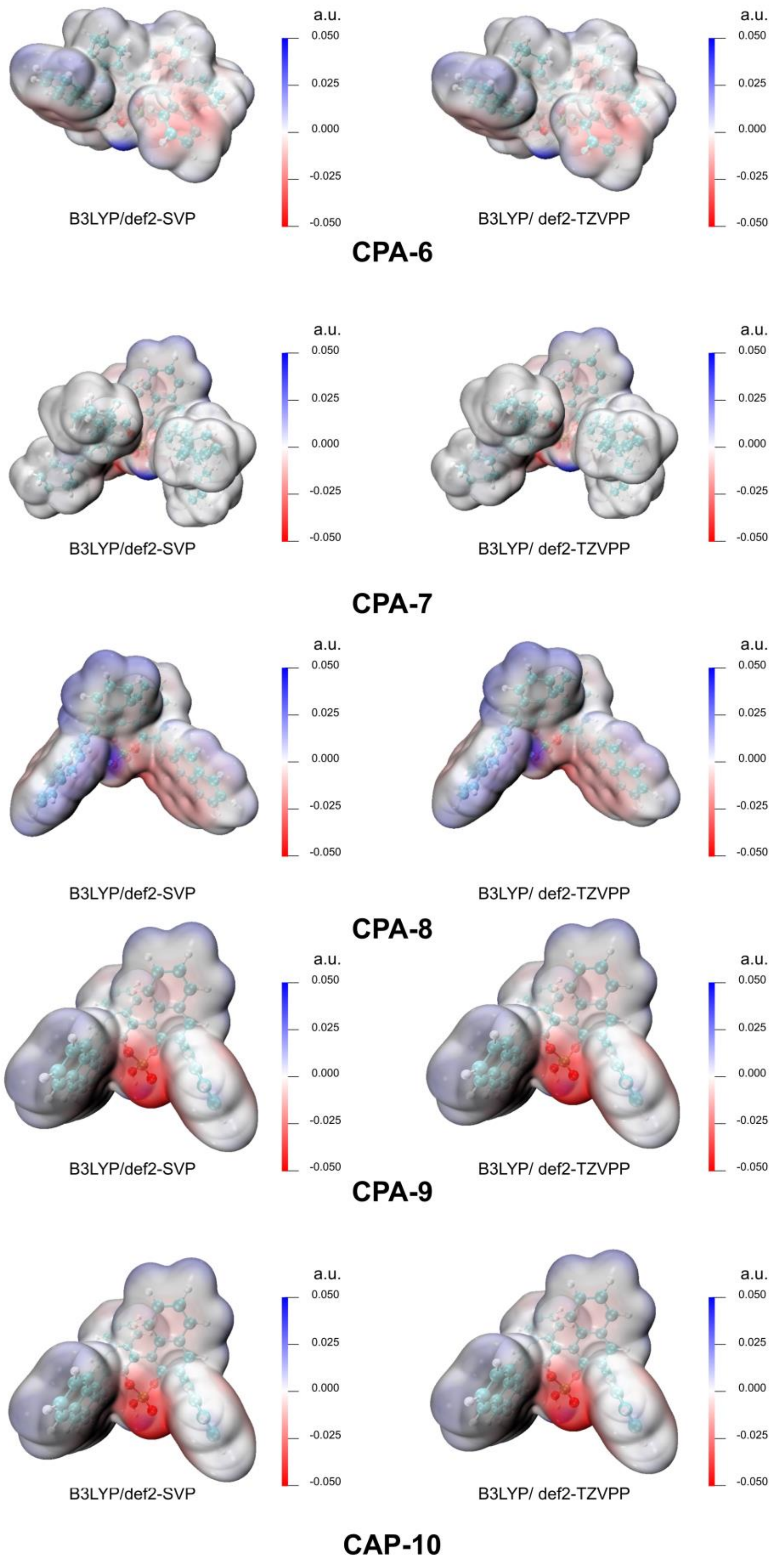

Supplementary Figure 10B. Electrostatic potential surfaces of the CPAs in the enantioselectivity dataset calculated at the B3LYP/def2-SVP level and the B3LYP/ def2-TZVPP level. "a.u." means atomic units.

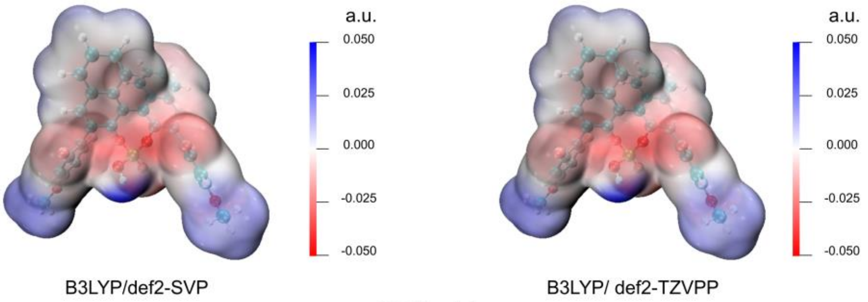

CPA-11

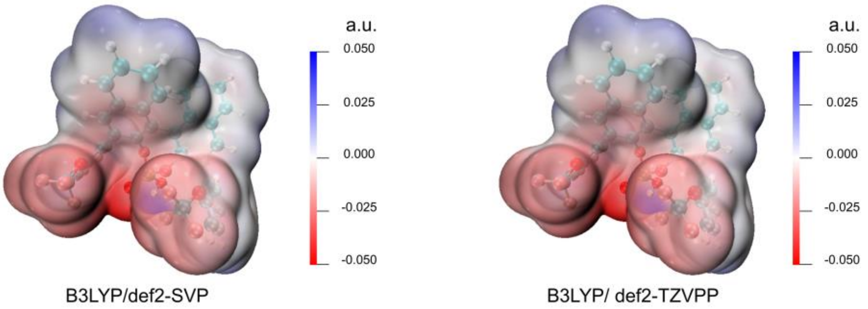

CPA-12

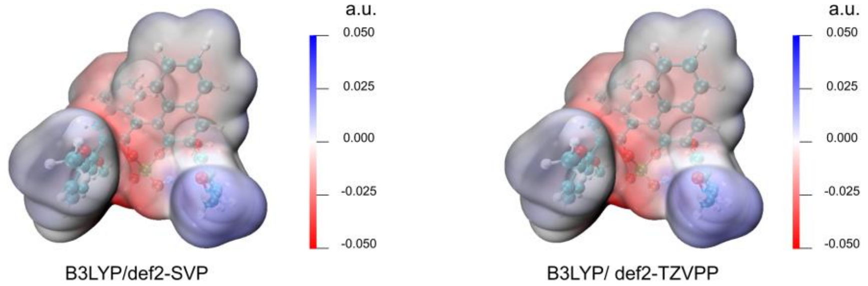

CPA-13

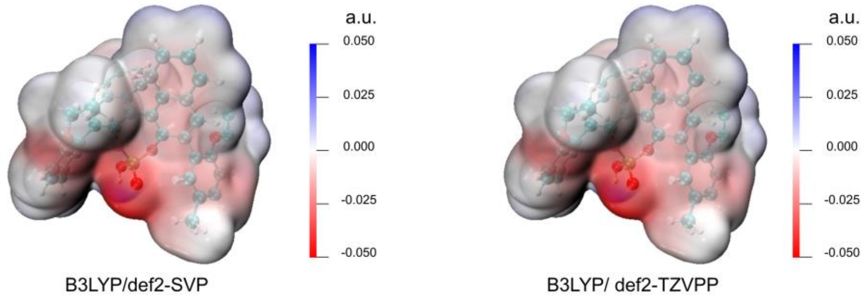

CPA-14

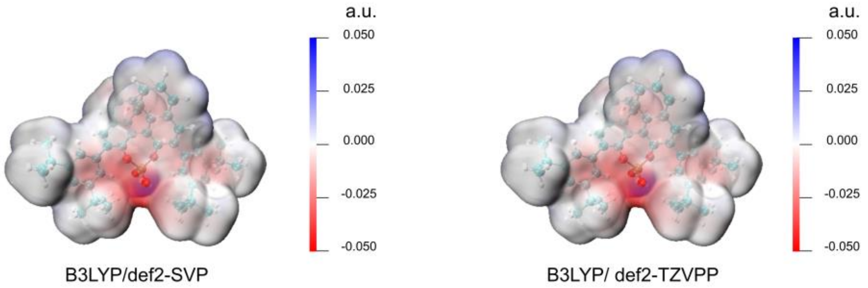

CAP-15

Supplementary Figure 10C. Electrostatic potential surfaces of the CPAs in the enantioselectivity dataset calculated at the B3LYP/def2-SVP level and the B3LYP/ def2-TZVPP level. "a.u." means atomic units.

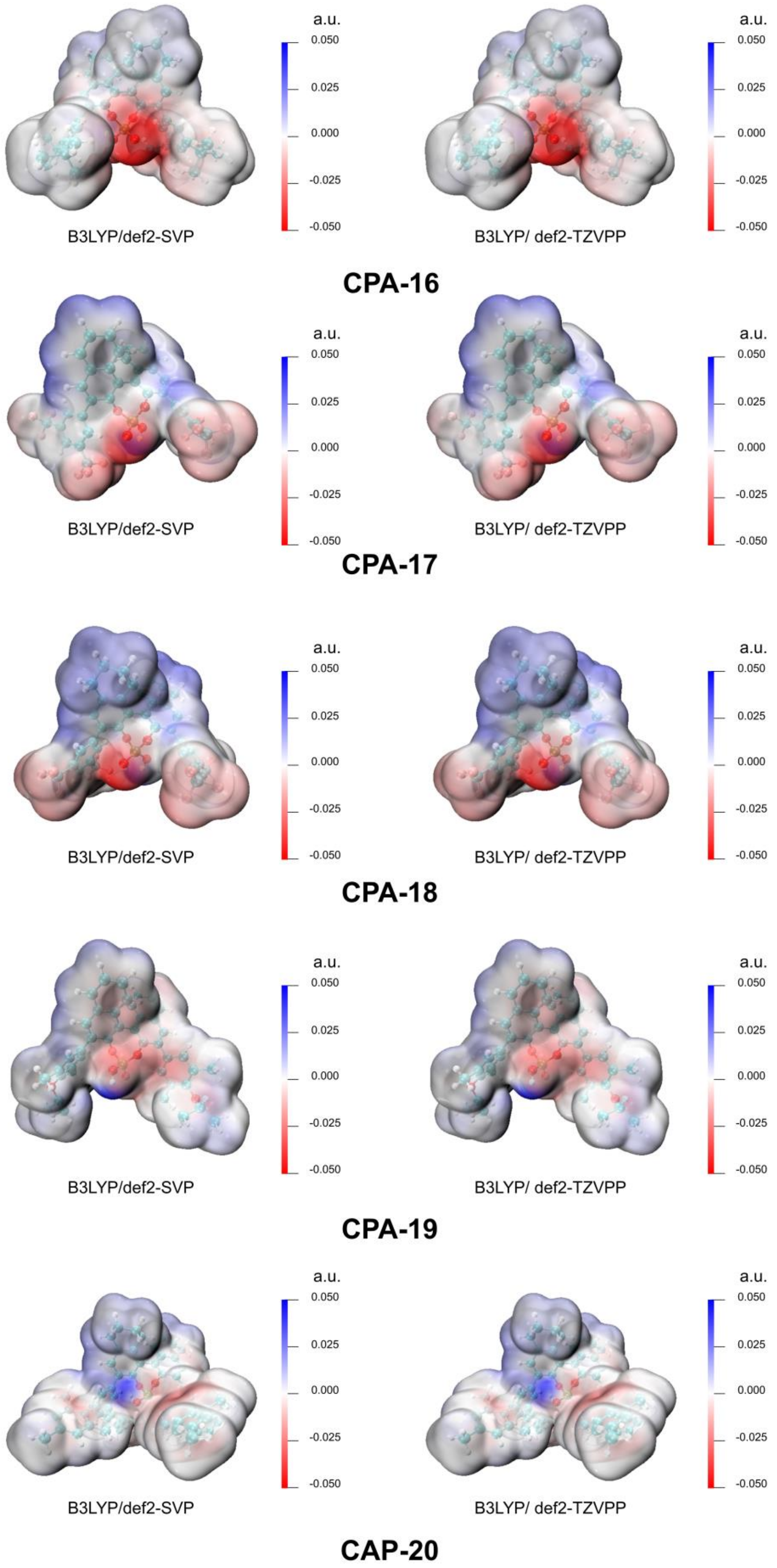

Supplementary Figure 10D. Electrostatic potential surfaces of the CPAs in the enantioselectivity dataset calculated at the B3LYP/def2-SVP level and the B3LYP/ def2-TZVPP level. "a.u." means atomic units.

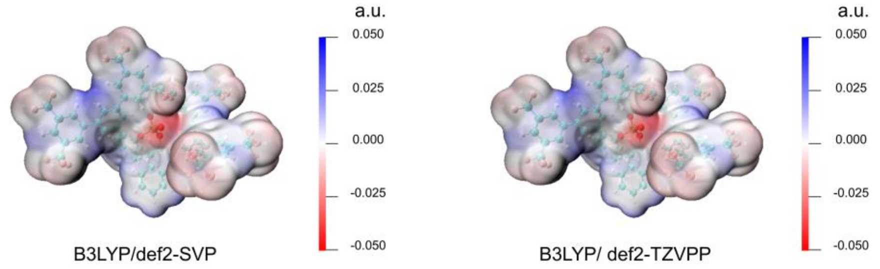

CPA-21

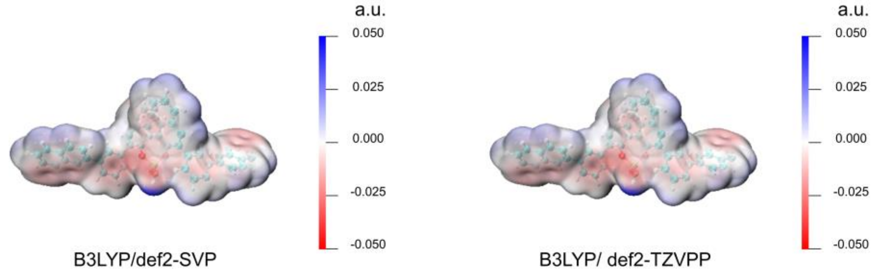

CPA-22

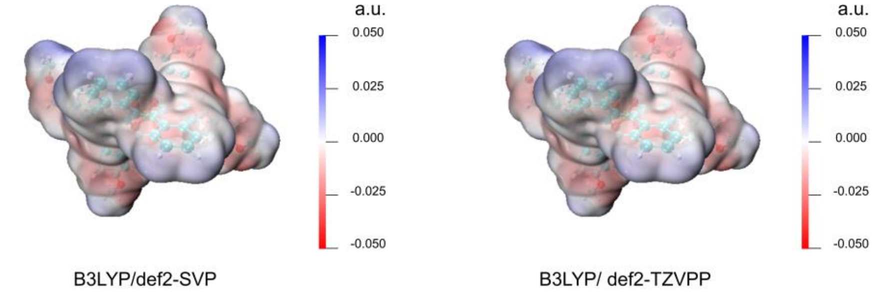

CPA-23

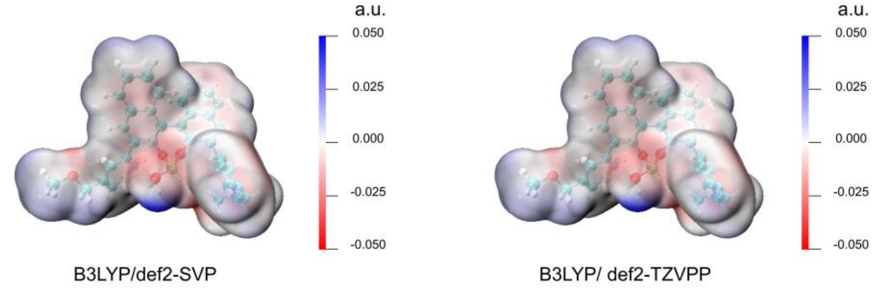

CPA-24

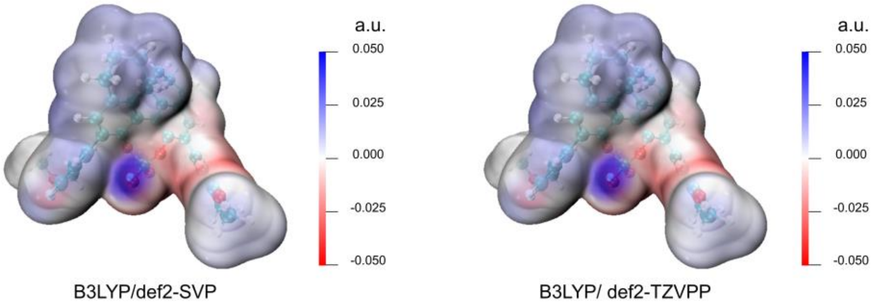

CAP-25

Supplementary Figure 10E. Electrostatic potential surfaces of the CPAs in the enantioselectivity dataset calculated at the B3LYP/def2-SVP level and the B3LYP/ def2-TZVPP level. "a.u." means atomic units.

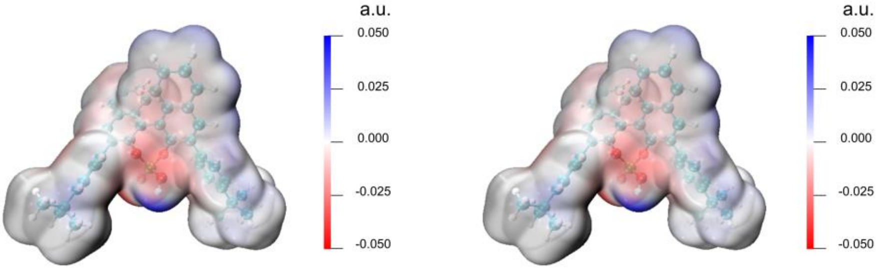

B3LYP/def2-SVP B3LYP/ def2-TZVPP

CPA-26

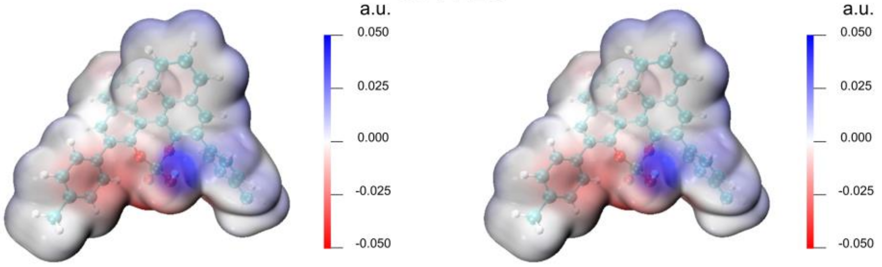

B3LYP/def2-SVP B3LYP/ def2-TZVPP

CPA-27

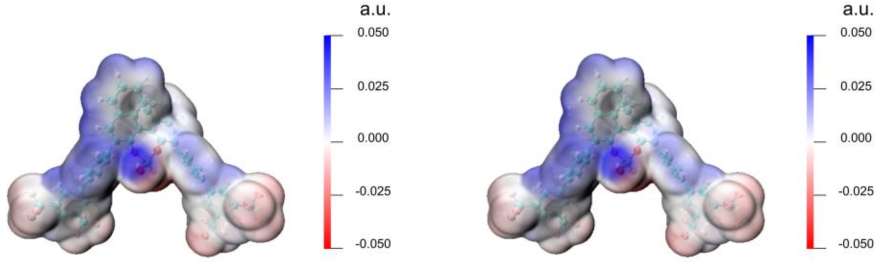

B3LYP/def2-SVP B3LYP/ def2-TZVPP

CPA-28

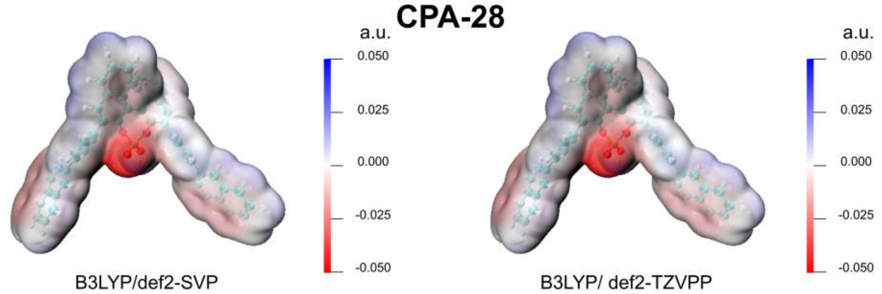

B3LYP/def2-SVP B3LYP/ def2-TZVPP

CPA-29

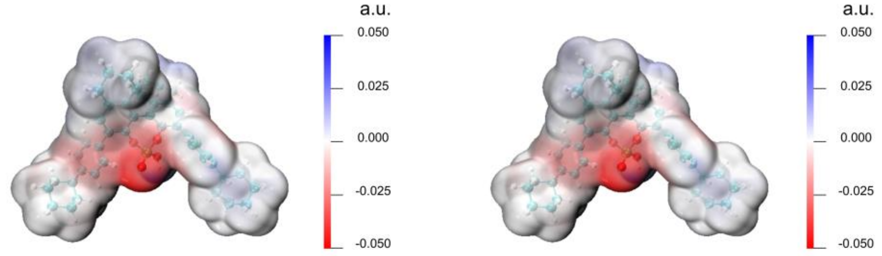

B3LYP/def2-SVP B3LYP/ def2-TZVPP

CAP-30

Supplementary Figure 10F. Electrostatic potential surfaces of the CPAs in the enantioselectivity dataset calculated at the B3LYP/def2-SVP level and the B3LYP/ def2-TZVPP level. "a.u." means atomic units.

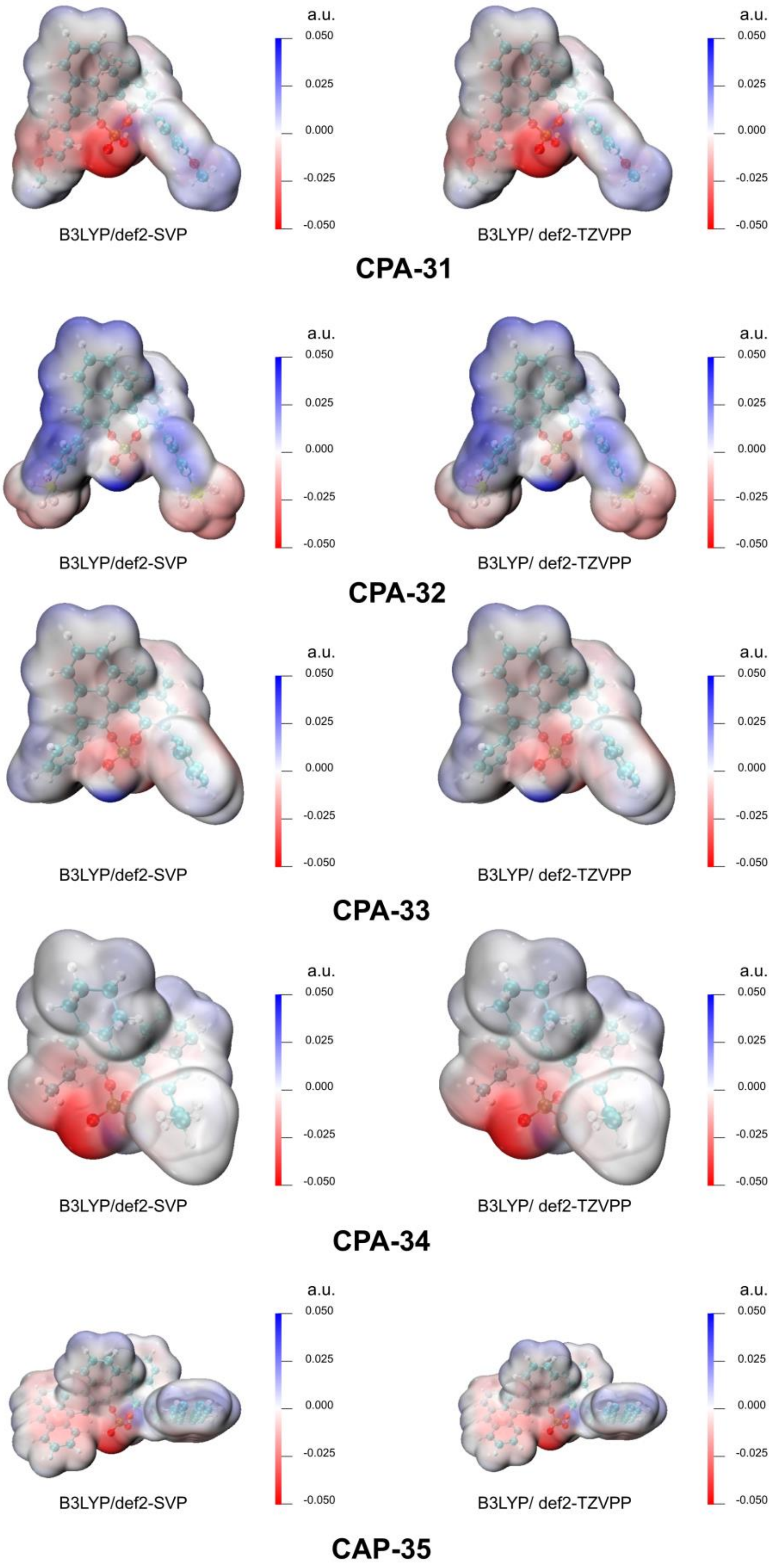

Supplementary Figure 10G. Electrostatic potential surfaces of the CPAs in the enantioselectivity dataset calculated at the B3LYP/def2-SVP level and the B3LYP/ def2-TZVPP level. "a.u." means atomic units.

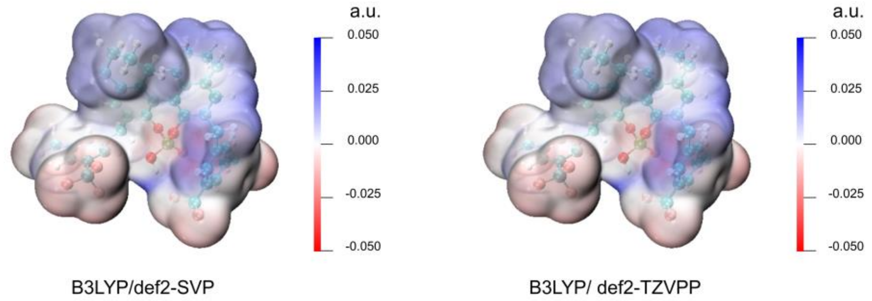

CPA-36

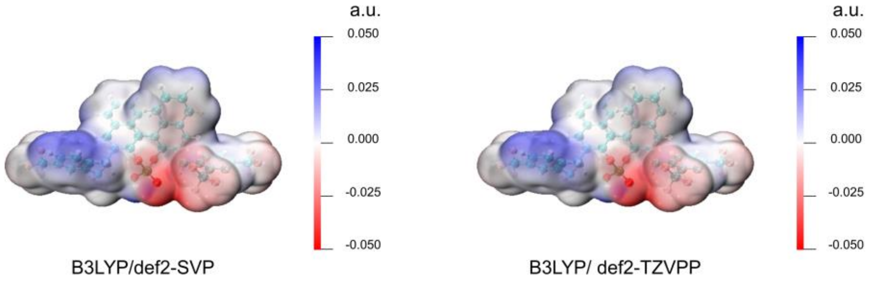

CPA-37

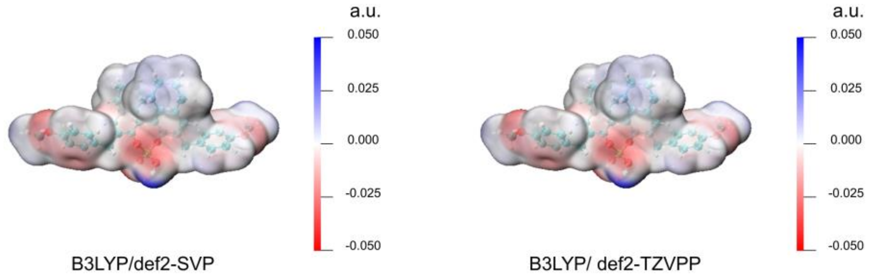

CPA-38

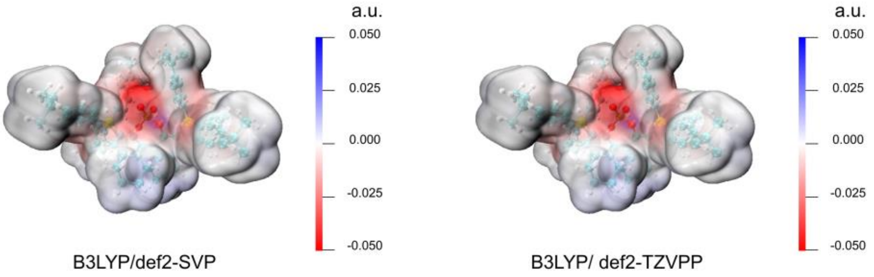

CPA-39

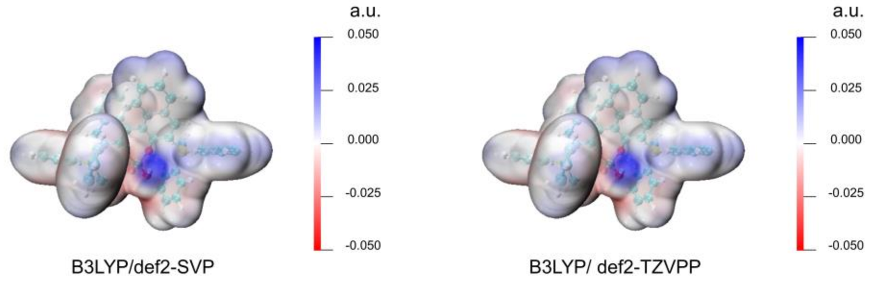

CAP-40

Supplementary Figure 10H. Electrostatic potential surfaces of the CPAs in the enantioselectivity dataset calculated at the B3LYP/def2-SVP level and the B3LYP/ def2-TZVPP level. "a.u." means atomic units.

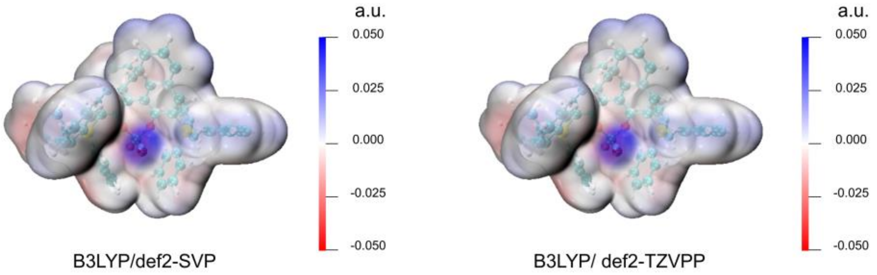

CPA-41

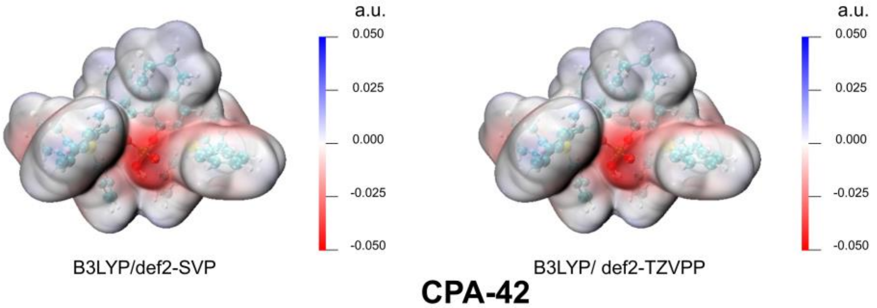

CPA-42

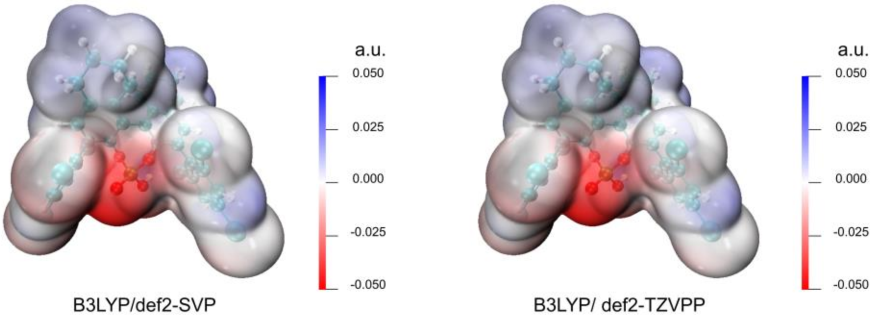

CPA-43

Supplementary Figure 10I. Electrostatic potential surfaces of the CPAs in the enantioselectivity dataset calculated at the B3LYP/def2-SVP level and the B3LYP/ def2-TZVPP level. "a.u." means atomic units.

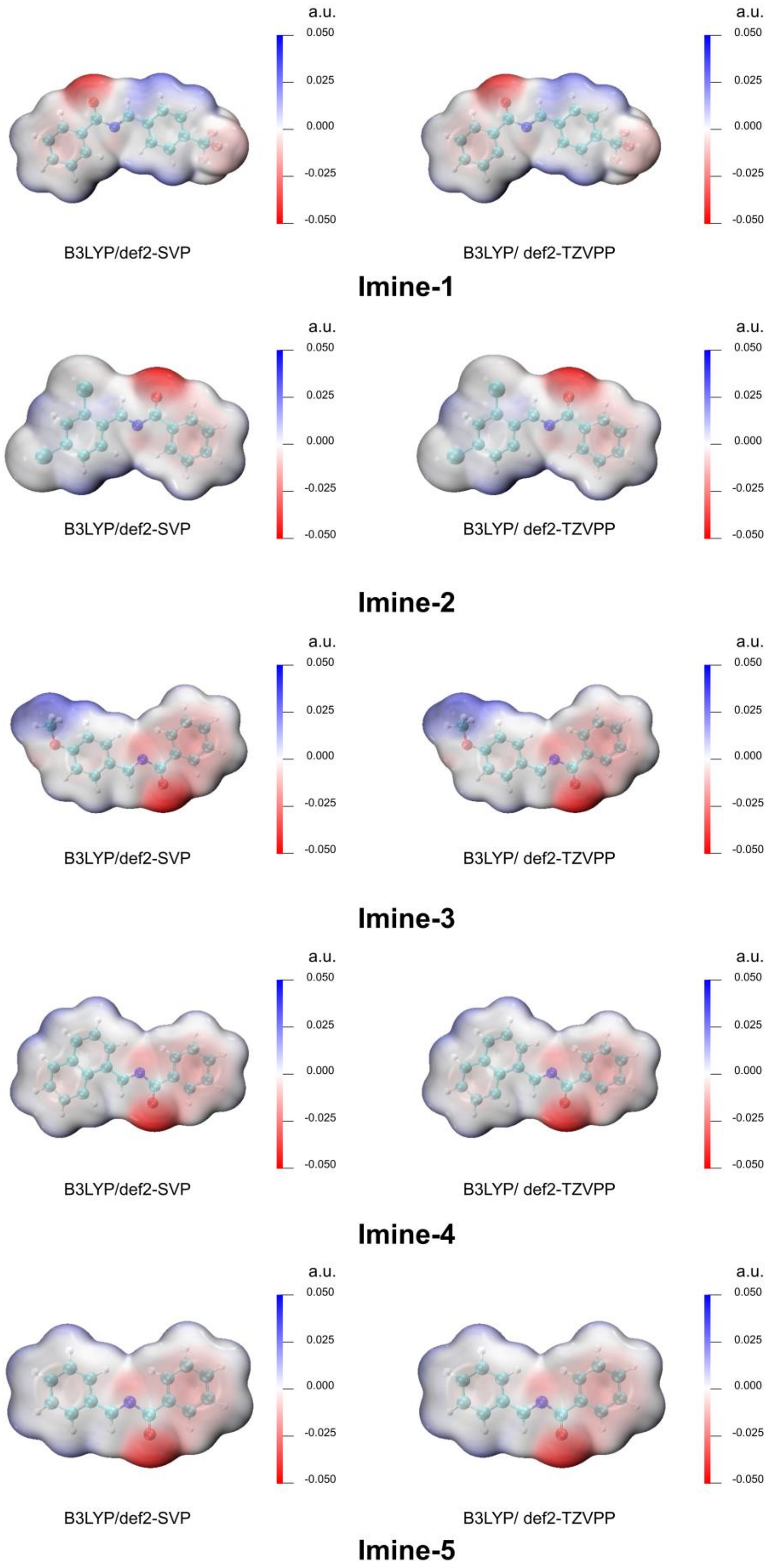

Supplementary Figure 11. Electrostatic potential surfaces of the imines in the enantioselectivity dataset calculated at the B3LYP/def2-SVP level and the B3LYP/ def2-TZVPP level. "a.u." means atomic units.

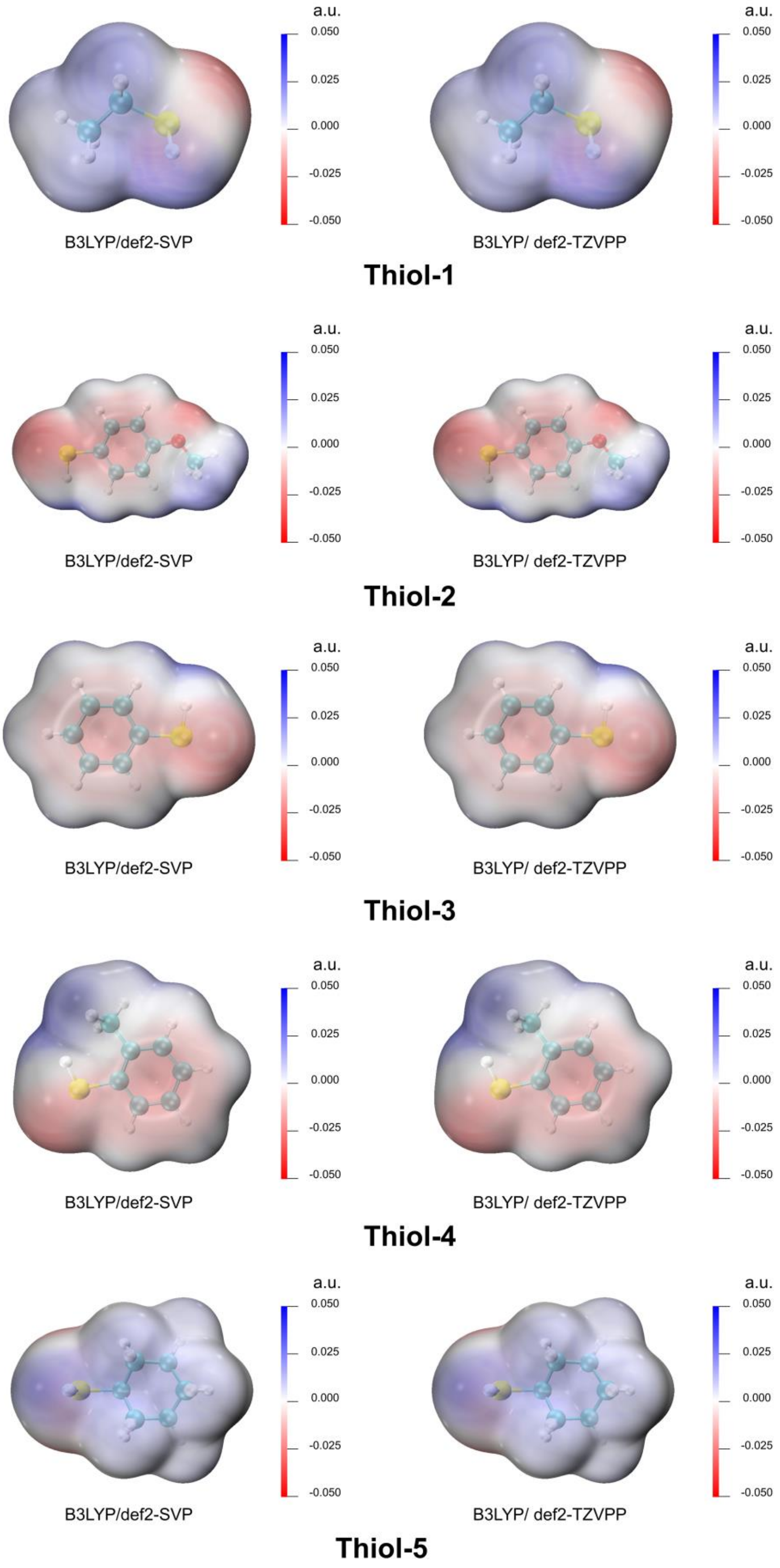

Supplementary Figure 12. Electrostatic potential surfaces of the thiols in the enantioselectivity dataset calculated at the B3LYP/def2-SVP level and the B3LYP/ def2-TZVPP level. "a.u." means atomic units.

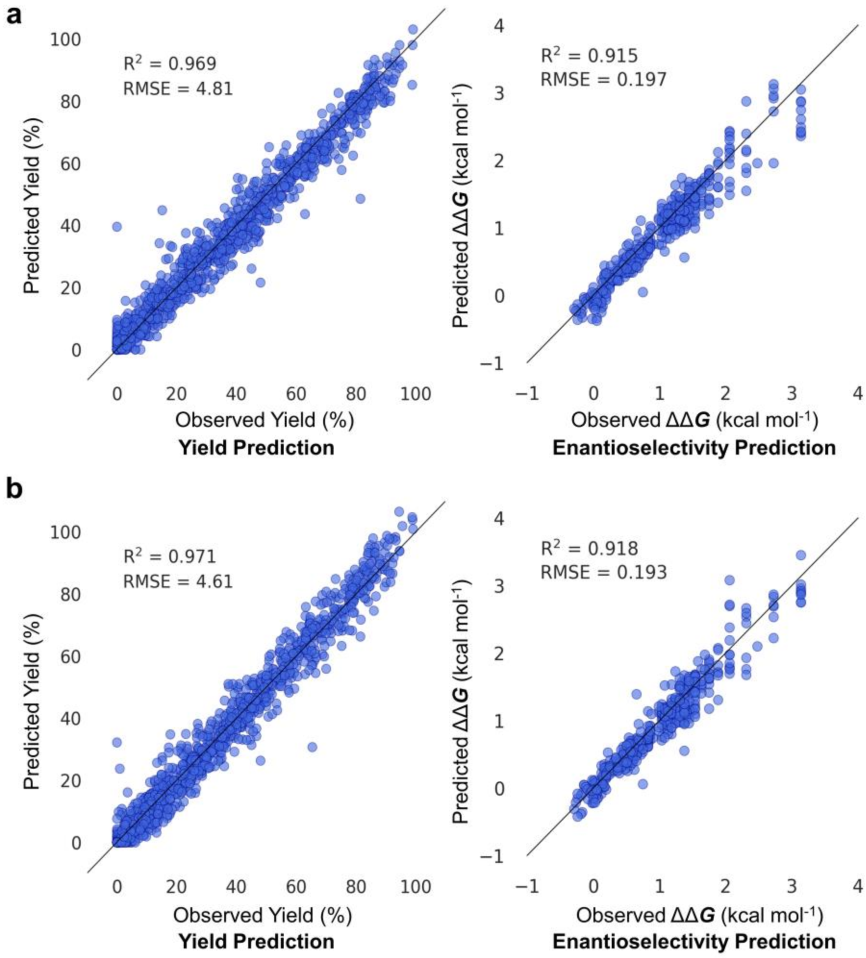

Supplementary Figure 13. Test set performances of the SEMG-MIGNN models (Sterics- and Electronics-embedded Molecular Graph and Molecular Interaction Graph Neural Network) trained by the electron density inputs calculated at the B3LYP/def2-SVP level (a) and the B3LYP/def2-TZVPP level (b). The yield dataset is randomly split to 70% (training) and 30% (test). The enantioselectivity task is randomly split to 600 (training) and 475 (test) transformations.

3. Results of machine learning predictions

3.1 Results of the yield regression performances of Baseline MG-GCN

We performed ten trials of yield prediction using the Baseline MG-GCN model. The detailed prediction results are provided in Supplementary Table 6. Entry 8 is selected as a representative example in Figure 4 of main text.

Supplementary Table 6. Results of yield prediction for Pd-catalyzed C–N cross coupling reactions using Baseline MG-GCN model. The yield dataset is randomly split to 70% (training) and 30% (test). RMSE means Root Mean Square Error.

Trial	RMSE (%)	R^2
1	18.11	0.560
2	18.67	0.532
3	18.36	0.547
4	18.61	0.535
5	18.32	0.549
6	18.47	0.542
7	18.32	0.549
8	18.40	0.545
9	18.43	0.544
10	18.20	0.555
Average	18.39	0.546

3.2 Results of the yield regression performances of SEMG-GCN

We performed ten trials of yield prediction using the SEMG-GCN model. SEMG means Sterics- and Electronics-embedded Molecular Graph. The detailed prediction results are provided in Supplementary Table 7. Entry 3 is selected as a representative example in Figure 4 of main text.

Supplementary Table 7. Results of yield prediction for Pd-catalyzed C–N cross coupling reactions using SEMG-GCN model. SEMG means Sterics- and Electronics-embedded Molecular Graph. The yield dataset is randomly split to 70% (training) and 30% (test). RMSE means Root Mean Square Error.

Trial	RMSE (%)	R^2
1	17.68	0.585
2	17.42	0.598
3	17.56	0.592
4	17.51	0.595
5	17.51	0.593
6	17.57	0.589
7	17.54	0.592
8	17.62	0.583
9	17.49	0.594
10	17.60	0.588
Average	17.55	0.591

3.3 Results of the yield regression performances of Baseline MG-MIGNN

We performed ten trials of yield prediction using the Baseline MG-MIGNN model. MIGNN means Molecular Interaction Graph Neural Network. The detailed prediction results are provided in Supplementary Table 8. Entry 8 is selected as a representative example in Figure 4 of main text.

Supplementary Table 8. Results of yield prediction for Pd-catalyzed C–N cross coupling reactions using Baseline MG-MIGNN model. The yield dataset is randomly split to 70% (training) and 30% (test). MIGNN means Molecular Interaction Graph Neural Network. RMSE means Root Mean Square Error.

Trial	RMSE (%)	R^2
1	7.63	0.923
2	8.87	0.898
3	6.90	0.935
4	7.21	0.930
5	8.77	0.897
6	7.39	0.927
7	7.33	0.928
8	7.69	0.921
9	6.97	0.935
10	8.20	0.910
Average	7.70	0.920

3.4 Results of the yield regression performances of SEMG-MIGNN (Sterics- and Electronics-embedded Molecular Graph and Molecular Interaction Graph Neural Network)

We performed ten trials of yield prediction using the SEMG-MIGNN model (Sterics- and Electronics-embedded Molecular Graph and Molecular Interaction Graph Neural Network). The detailed prediction results are provided in Supplementary Table 9. Entry 6 is selected as a representative example in Figure 4 of main text.

Supplementary Table 9. Results of yield prediction for Pd-catalyzed C–N cross coupling reactions using SEMG-MIGNN model (Sterics- and Electronics-embedded Molecular Graph and Molecular Interaction Graph Neural Network). The yield dataset is randomly split to 70% (training) and 30% (test). RMSE means Root Mean Square Error.

Trial	RMSE (%)	R^2
1	4.51	0.975
2	4.59	0.972
3	4.61	0.972
4	4.41	0.975
5	5.20	0.965
6	4.81	0.969
7	4.78	0.969
8	4.85	0.968
9	5.13	0.965
10	5.08	0.967
Average	4.80	0.970

3.5 Results of the enantioselectivity regression performances of Baseline MG-GCN

We performed ten trials of enantioselectivity prediction using the Baseline MG-GCN model. The detailed prediction results are provided in Supplementary Table 10. Entry 2 is selected as a representative example in Figure 5 of main text.

Supplementary Table 10. Results of enantioselectivity prediction for chiral phosphoric acid-catalyzed thiol addition to *N*-acylimines using Baseline MG-GCN model. The enantioselectivity dataset is randomly split to 600 (training) and 475 (test). RMSE means Root Mean Square Error.

Trial	RMSE (kcal mol^{-1})	R^2
1	0.356	0.744
2	0.332	0.778
3	0.329	0.781
4	0.320	0.792
5	0.325	0.787
6	0.338	0.770
7	0.351	0.751
8	0.329	0.781
9	0.334	0.774
10	0.307	0.810
Average	0.332	0.777

3.6 Results of the enantioselectivity regression performances of SEMG-GCN

We performed ten trials of enantioselectivity prediction using the SEMG-GCN model. SEMG means Sterics- and Electronics-embedded Molecular Graph. The detailed prediction results are provided in Supplementary Table 11. Entry 1 is selected as a representative example in Figure 5 of main text.

Supplementary Table 11. Results of enantioselectivity prediction for chiral phosphoric acid-catalyzed thiol addition to *N*-acylimines using SEMG-GCN model. SEMG means Sterics- and Electronics-embedded Molecular Graph. The enantioselectivity dataset is randomly split to 600 (training) and 475 (test). RMSE means Root Mean Square Error.

Trial	RMSE (kcal mol^{-1})	R^2
1	0.293	0.819
2	0.312	0.795
3	0.284	0.830
4	0.299	0.810
5	0.301	0.811
6	0.292	0.817
7	0.288	0.821
8	0.283	0.830
9	0.300	0.807
10	0.298	0.812
Average	0.295	0.815

3.7 Results of the enantioselectivity regression performances of Baseline MG-MIGNN

We performed ten trials of enantioselectivity prediction using the Baseline MG-MIGNN model. MIGNN means Molecular Interaction Graph Neural Network. The detailed prediction results are provided in Supplementary Table 12. Entry 6 is selected as a representative example in Figure 5 of main text.

Supplementary Table 12. Results of enantioselectivity prediction for chiral phosphoric acid-catalyzed thiol addition to *N*-acylimines using Baseline MG-MIGNN model. MIGNN means Molecular Interaction Graph Neural Network. The enantioselectivity dataset is randomly split to 600 (training) and 475 (test). RMSE means Root Mean Square Error.

Trial	RMSE (kcal mol^{-1})	R^2
1	0.251	0.870
2	0.236	0.884
3	0.256	0.869
4	0.234	0.890
5	0.258	0.861
6	0.240	0.880
7	0.260	0.862
8	0.235	0.887
9	0.233	0.885
10	0.250	0.870
Average	0.245	0.876

3.8 Results of the enantioselectivity regression performances of SEMG-MIGNN (Sterics- and Electronics-embedded Molecular Graph and Molecular Interaction Graph Neural Network)

We performed ten trials of enantioselectivity prediction using the SEMG-MIGNN model (Sterics- and Electronics-embedded Molecular Graph and Molecular Interaction Graph Neural Network). The detailed prediction results are provided in Supplementary Table 13. Entry 3 was selected as a representative example in Figure 5 of main text.

Supplementary Table 13. Results of enantioselectivity prediction for chiral phosphoric acid-catalyzed thiol addition to *N*-acylimines using SEMG-MIGNN model (Sterics- and Electronics-embedded Molecular Graph and Molecular Interaction Graph Neural Network). The enantioselectivity dataset is randomly split to 600 (training) and 475 (test). RMSE means Root Mean Square Error.

Trial	RMSE (kcal mol^{-1})	R^2
1	0.189	0.917
2	0.188	0.920
3	0.197	0.915
4	0.197	0.913
5	0.190	0.918
6	0.199	0.911
7	0.197	0.916
8	0.202	0.910
9	0.200	0.914
10	0.199	0.913
Average	0.196	0.915

3.9 Results of other tested descriptors

We tested four widely applied molecular descriptors (One-Hot, RDKit descriptors, Morgan Fingerprint, and Atom-centered Symmetry Functions) using thirteen regression algorithms (AdaBoost, Bagging Regression, Decision Trees, Extra-Trees, Gradient Boosting, k-Nearest Neighbors Regression, Kernel Ridge Regression, Linear Support Vector Regression, Random Forest Regression, Ridge, Support Vector Regression, XGBoost, and Neural Network) to predict the yields of C–N cross coupling reaction and the enantioselectivity of asymmetric *N*,*S*-acetal formation. Supplementary Figure 14 shows the results of yield predictions, and Supplementary Figure 15 shows the results of enantioselectivity predictions.

Using the best algorithm for each type of descriptor, hyperparameter optimization was applied. The details of the hyperparameter optimization are provided in Supplementary Table 4, and the optimization results are summarized in Supplementary Table 14 and Supplementary Table 15. The optimized hyperparameters were subsequently used in the related machine learnings.

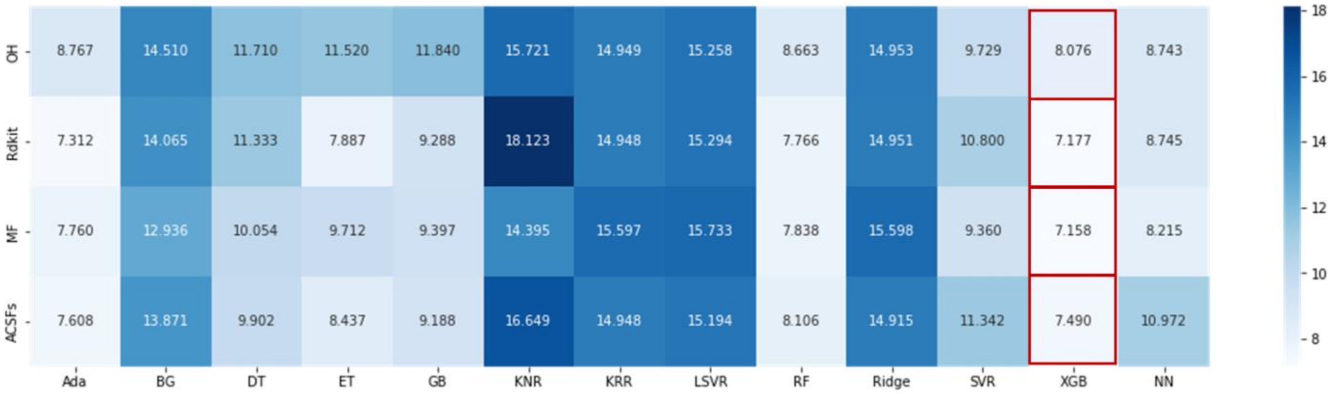

Supplementary Figure 14. RMSEs (Root Mean Square Errors, in %) of yield predictions using widely applied molecular descriptors. The yield dataset is randomly split to 70% (training) and 30% (test). The red frame represents the best model corresponding to the four baseline descriptors.

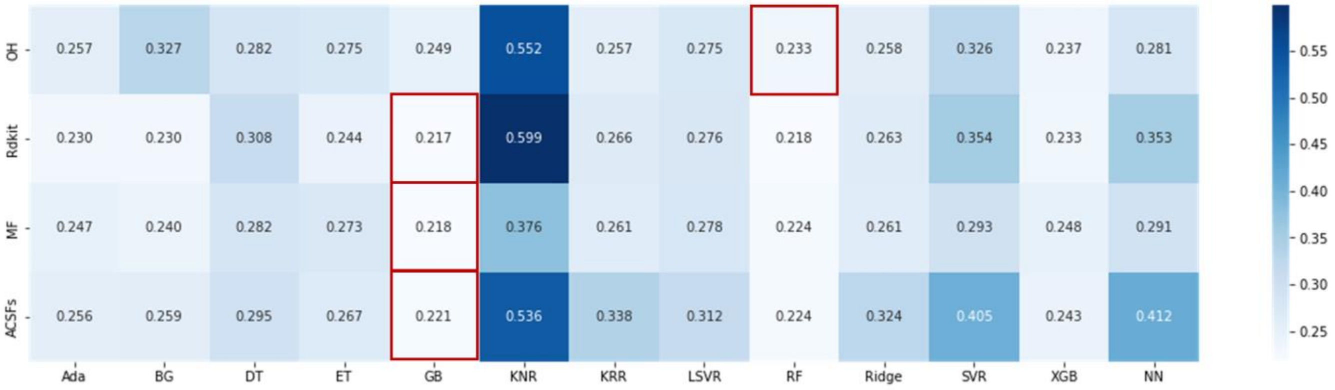

Supplementary Figure 15. RMSEs (Root Mean Square Errors, in kcal mol^{-1}) of enantioselectivity predictions using widely applied molecular descriptors. The enantioselectivity dataset is randomly split to 600 (training) and 475 (test). The red frame represents the best model corresponding to the four baseline descriptors.

Supplementary Table 14. Modelling performances in yield tasks after the hyperparameter optimization. The yield dataset is randomly split to 70% (training) and 30% (test). SEMG means Sterics- and Electronics-embedded Molecular Graph. MIGNN means Molecular Interaction Graph Neural Network. RMSE means Root Mean Square Error.

Target	Descriptor Name	Model Name	R^2 of Optimal Parameters	RMSE (in %) of Optimal Parameters
Yield	OH	XGBoost	0.912	8.048
Yield	RDKit	XGBoost	0.934	6.960
Yield	MF	XGBoost	0.938	6.761
Yield	ACSFs	XGBoost	0.929	7.238
Yield	Baseline MG	GCN	0.546	18.39
Yield	SEMG	GCN	0.591	17.55
Yield	Baseline MG	MIGNN	0.920	7.700
Yield	SEMG	MIGNN	0.970	4.800

Supplementary Table 15. Modelling performances in enantioselectivity tasks after the hyperparameter optimization. The enantioselectivity task is randomly split to 600 (training) and 475 (test) transformations. SEMG means Sterics- and Electronics-embedded Molecular Graph. MIGNN means Molecular Interaction Graph Neural Network. RMSE means Root Mean Square Error.

Target	Descriptor Name	Model Name	R^2 of Optimal Parameters	RMSE (in kcal mol^{-1}) of Optimal Parameters
Enantioselectivity	OH	RandomForest	0.885	0.233
Enantioselectivity	RDKit	Gradient Boosting	0.900	0.217
Enantioselectivity	MF	Gradient Boosting	0.901	0.217
Enantioselectivity	ACSFs	Gradient Boosting	0.900	0.217
Enantioselectivity	Baseline MG	GCN	0.777	0.332
Enantioselectivity	SEMG	GCN	0.815	0.295
Enantioselectivity	Baseline MG	MIGNN	0.876	0.245
Enantioselectivity	SEMG	MIGNN	0.915	0.196

3.10 Evaluation of structural sensitivity of the SEMG-MIGNN model (Sterics- and Electronics-embedded Molecular Graph and Molecular Interaction Graph Neural Network)

We tested the impact of the initial structure on the modelling performance. Ten different random seeds were applied for the generation of the molecular structures using the EmbedMolecule module of RDKit. Subsequently, we performed the geometry optimizations and electronic structure calculations through the same process. The changes in prediction performances are summarized in Supplementary Table 16 (yield task) and Supplementary Table 17 (enantioselectivity task), which showed marginal influence from the selection of random seed. These additional results demonstrate that the model is not sensitive to the initial random seed for structural generation. The corresponding random seeds are also provided on our GitHub repository (https://github.com/Shuwen-Li/SEMG-MIGNN) for readers to reproduce.

Supplementary Table 16. Modelling performances in yield prediction task (70% training and 30% test) using different random seeds for the generation of initial structure. RMSE means Root Mean Square Error.

Seed	RMSE (%)	R^2
1	4.88	0.968
2	4.59	0.972
3	4.79	0.969
4	4.41	0.975
5	5.19	0.964
6	4.79	0.969
7	4.50	0.974
8	4.85	0.968
9	5.11	0.965
10	4.71	0.970

Supplementary Table 17. Modelling performances in enantioselectivity prediction task (600 training and 475 test) using different random seeds for the generation of initial structure. RMSE means Root Mean Square Error.

Seed	RMSE (kcal mol^{-1})	R^2
1	0.199	0.912
2	0.206	0.907
3	0.196	0.915
4	0.199	0.913
5	0.190	0.918
6	0.203	0.909
7	0.186	0.922
8	0.199	0.913
9	0.205	0.906
10	0.195	0.916

3.11 Learning curves of SEMG-MIGNN (Sterics- and Electronics-embedded Molecular Graph and Molecular Interaction Graph Neural Network)

We have explored the learning curve of the SEMG-MIGNN model (Sterics- and Electronics-embedded Molecular Graph and Molecular Interaction Graph Neural Network) with different ratio of training data (Supplementary Figure 16). We repeated the prediction ten times, and the shadow in the figure represents the range of error in these ten predictions. In both yield and enantioselectivity tasks, the SEMG-MIGNN model (Sterics- and Electronics-embedded Molecular Graph and Molecular Interaction Graph Neural Network) can achieve an acceptable performance with 20% of the training data, and its predictive ability approached convergence with 70% or more the training data.

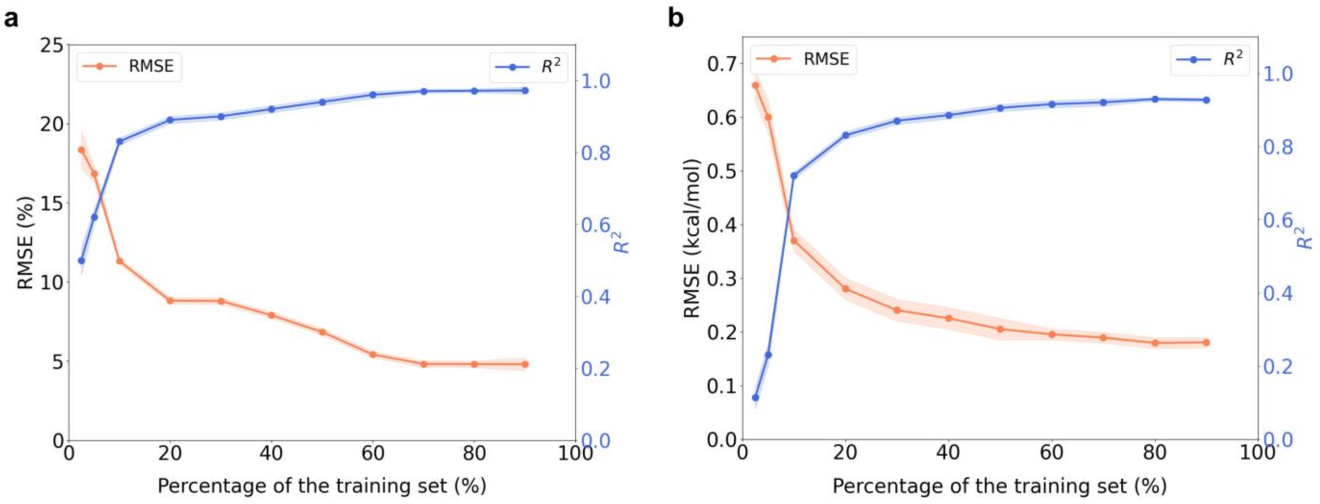

Supplementary Figure 16. Learning curves of the SEMG-MIGNN model (Sterics- and Electronics-embedded Molecular Graph and Molecular Interaction Graph Neural Network) in yield and enantioselectivity tasks. We repeated the prediction ten times, and the shadow in the figure represents the range of error in these ten predictions. a Learning curves of the SEMG-MIGNN model (Sterics- and Electronics-embedded Molecular Graph and Molecular Interaction Graph Neural Network) in yield task. **b** Learning curves of the SEMG-MIGNN model (Sterics- and Electronics-embedded Molecular Graph and Molecular Interaction Graph Neural Network) in enantioselectivity task.

3.12 Details of the machine learning modelling of the external experimental tests

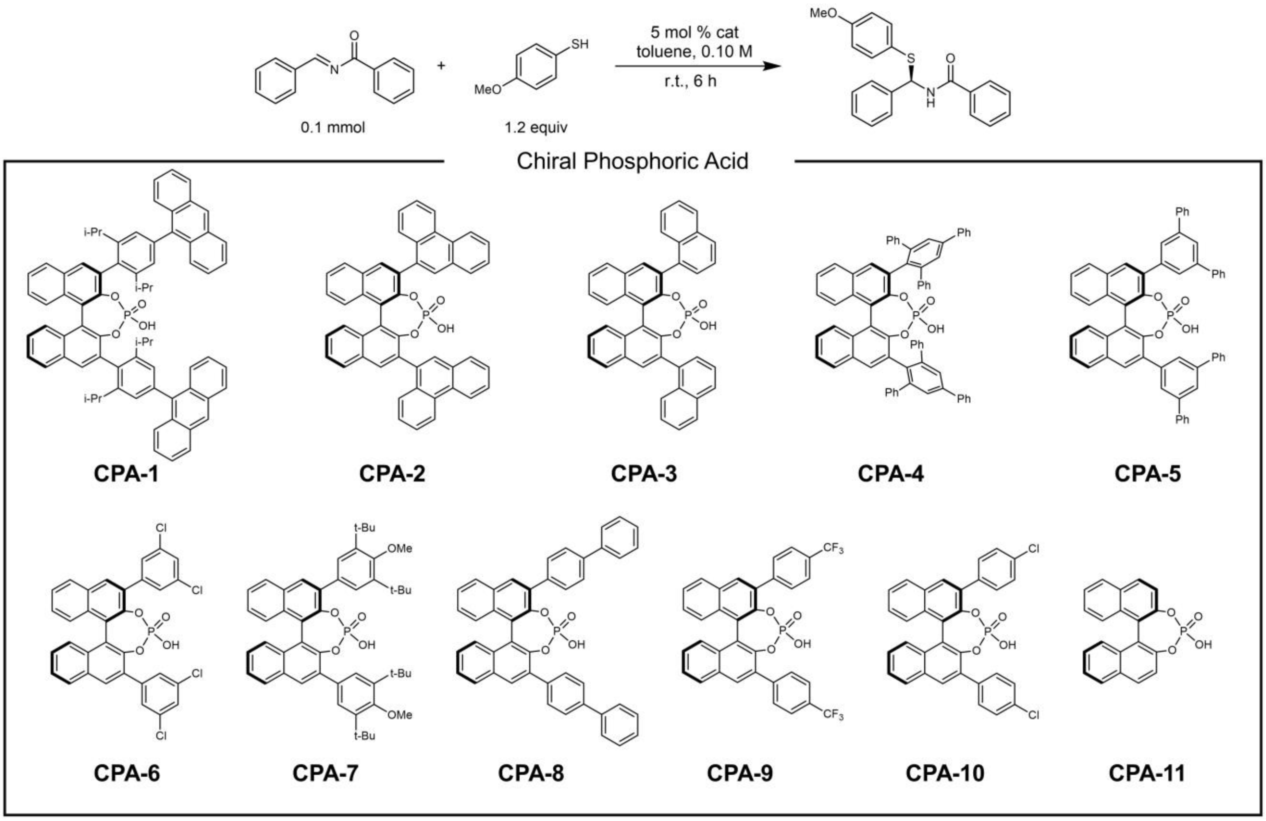

Supplementary Figure 17. Details of the 11 experimentally tested CPAs for external verifications of the SEMG-MIGNN (Sterics- and Electronics-embedded Molecular Graph and Molecular Interaction Graph Neural Network) predictions.

To evaluate the structural differences between the 43 CPAs in Denmark's dataset and the 11 CPAs (Supplementary Figure 17) in our experimental tests, we used the correlation coefficient of Morgan molecular fingerprints. The correlation coefficients of Tanimoto similarity is shown in Supplementary Figure 18a. These results indicated that the CPAs in our experimental evaluations have noticeable differences in terms of the topological structure. The median value of the correlation coefficient is 0.56, whose structures are shown in Supplementary Figure 18b. Supplementary Table 18 summarized the experimental and predicted enantioselectivities for the various tested models.

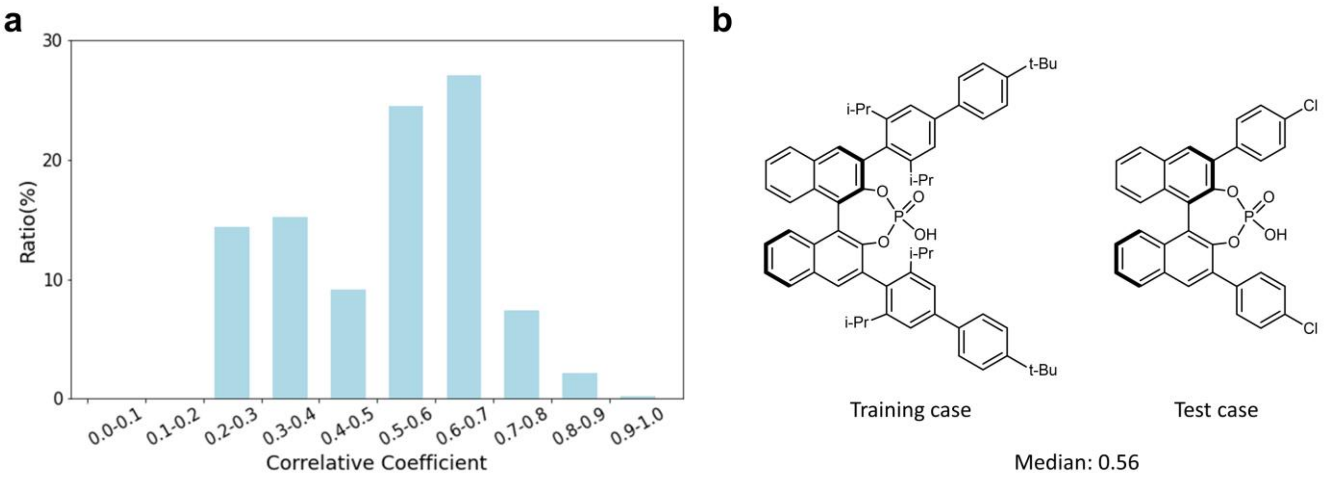

Supplementary Figure 18. Fingerprint similarity of CPAs between the training set and the external test set. a Distribution of the correlation coefficient of Tanimoto similarity using Morgan molecular fingerprints. **b** The pair of CPAs that has the median value of Fingerprint similarity.

Supplementary Table 18. Experimental and predicted enantioselectivities (in kcal mol^{-1}) for the experimentally tested chiral phosphoric acid catalysts. SEMG-MIGNN means Sterics- and Electronics-embedded Molecular Graph and Molecular Interaction Graph Neural Network.

CPA	Experimental Value	ACSFs/GB	DRFP[21]	MFF[22]	SEMG-MIGNN
1	1.15	1.812	1.826	1.882	1.046
2	0.98	0.858	1.177	1.037	0.765
3	0.92	0.857	1.500	1.004	0.710
4	0.84	1.725	1.438	1.281	0.724
5	0.77	1.543	1.360	0.883	0.798
6	0.73	0.932	1.154	0.895	0.750
7	0.68	0.799	1.155	0.883	0.763
8	0.67	0.943	1.342	1.308	0.826
9	0.65	0.851	1.358	0.836	0.487
10	0.52	0.827	1.365	0.857	0.557
11	0.08	0.316	1.154	0.280	0.289
R^2		-1.811	-5.207	-0.845	0.745
MAE (kcal mol^{-1})		0.349	0.622	0.287	0.117
RMAE (kcal mol^{-1})		0.443	0.658	0.359	0.127

3.13 Results of enantioselectivity regression performances on asymmetric hydrogenation of olefins

We further compared the performance of SEMG-MIGNN (Sterics- and Electronics-embedded Molecular Graph and Molecular Interaction Graph Neural Network) with MFF[22]-RF, DRFP[21]-XGB models on the asymmetric hydrogenation of olefins. The data of representative Rh/BINOL-phosphite-catalyzed hydrogenation reaction of tri-substituted olefins was used based on our previous database study[23]. The 10-fold cross validation performances are compared in Supplementary Figure 19. SEMG-MIGNN (Sterics- and Electronics-embedded Molecular Graph and Molecular Interaction Graph Neural Network) exhibited satisfying prediction performance in this transformation, with a R^2 of 0.777 and a RMSE (Root Mean Square Error) of 0.427 kcal mol^{-1}, which outperforms DRFP[21] and MFF[22] approaches.

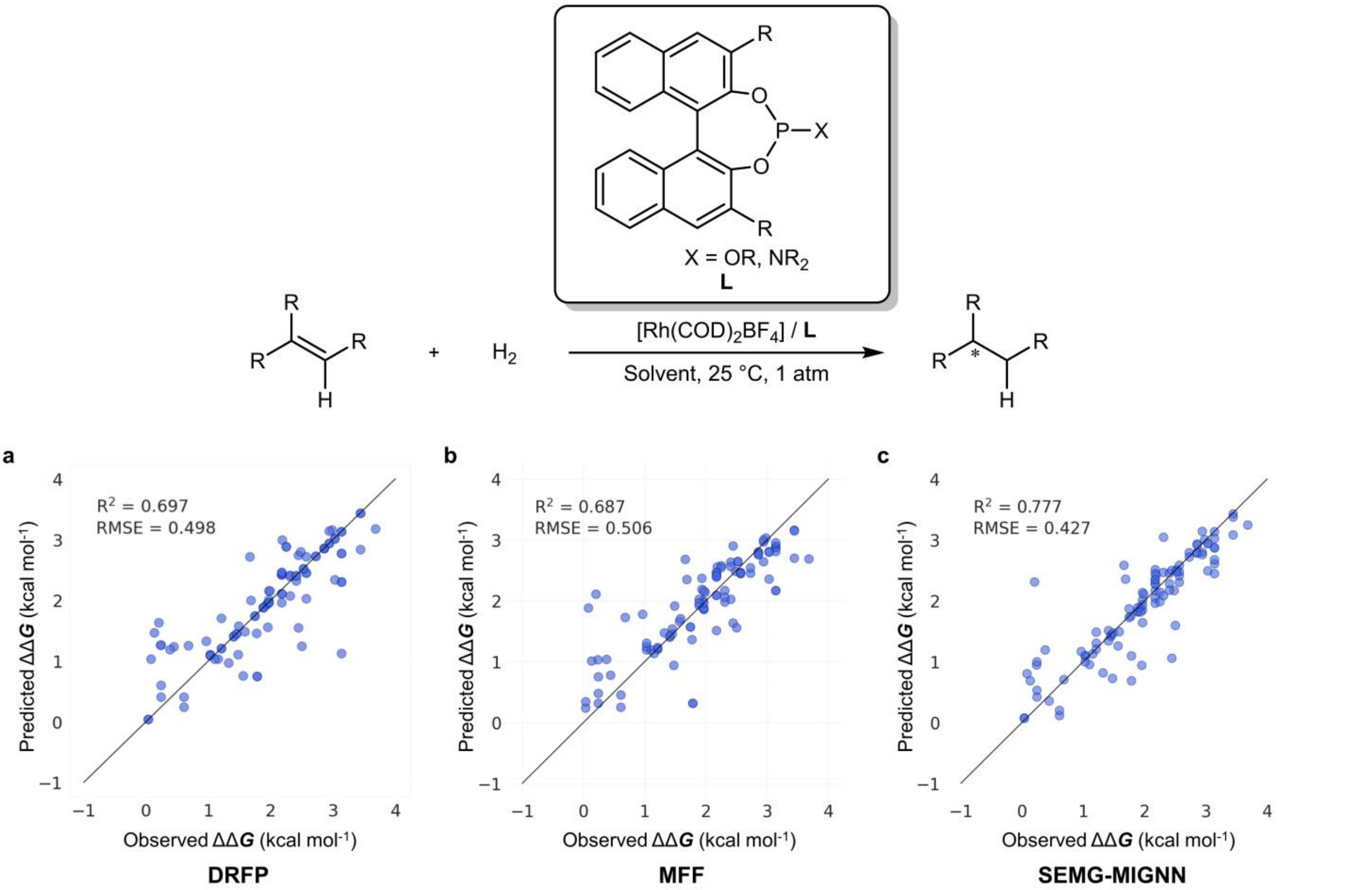

Supplementary Figure 19. Enantioselectivity prediction of Rh/BINOL-phosphite-catalyzed hydrogenation reaction of tri-substituted olefins using SEMG-MIGNN (Sterics- and Electronics-embedded Molecular Graph and Molecular Interaction Graph Neural Network), DRFP[21] (Differential Reaction Fingerprint) and MFF[22] (Multiple Fingerprint Feature) models. a Predicted performance of DRFP (Differential Reaction Fingerprint). **b** Predicted performance of MFF (Multiple Fingerprint Feature). **c** Predicted performance of SEMG-MIGNN (Sterics- and Electronics-embedded Molecular Graph and Molecular Interaction Graph Neural Network). The models are trained using 10-fold cross validation.

4. Comparison between SEMG-MIGNN (Sterics- and Electronics-embedded Molecular Graph and Molecular Interaction Graph Neural Network) and other SOTA models

4.1 Details of the tested SOTA models

For the Yield-BERT model[24], all the prediction tasks were trained using the default parameters of the original study[24].

For the DRFP[21] encoding, we used XGBoost algorithm as in the original study[21] and performed the hyperparameter optimization. The range of the hyperparameter optimization included: n_estimators= [100,200,300,400,500,600], max_depth= [None, 10, 20, 30]). The best parameters for yield tasks were n_estimators= 100 and max_depth= 10. The best parameters for enantioselectivity tasks were n_estimators= 100 and max_depth= None.

For the MFF[22] encoding, the best fingerprint for yield tasks was Morgan-Circular Fingerprint-radii 2 (3096 bits), and the best fingerprint for enantioselectivity tasks was RDKit linear Fingerprint-radii 6 (3096 bits). In addition, we used the Random Forest algorithm as in the original study[22] and performed hyperparameter optimization. The range of the hyperparameter optimization included: n_estimators: [100,200,300,400], max_depth: [None, 10, 20, 30]. The best parameters for yield tasks were n_estimators= 100 and max_depth= None. The best parameters for enantioselectivity tasks were n_estimators= 100 and max_depth= None.

4.2 Yield prediction in C–N cross coupling reaction

We compared the SEMG-MIGNN model (Sterics- and Electronics-embedded Molecular Graph and Molecular Interaction Graph Neural Network) with the Yield BERT, DRFP, MFF models in a series of yield prediction tasks. We tested 13 prediction tasks (Supplementary Table 19), including different ratios of random data splitting and extrapolative predictions for 4 additives. In the random data splitting, SEMG-MIGNN (Sterics- and Electronics-embedded Molecular Graph and Molecular Interaction Graph Neural Network) outperformed the other three tested models in all nine tasks. For the extrapolative predictions of the additives, SEMG-MIGNN (Sterics- and Electronics-embedded Molecular Graph and Molecular Interaction Graph Neural Network) achieved the best performance in tests 2 to 4.

Supplementary Table 19. Prediction performances of yield tasks using various SOTA models. SEMG-MIGNN means Sterics- and Electronics-embedded Molecular Graph and Molecular Interaction Graph Neural Network.

Data Splitting	R^2 of Yield-BERT[24]	R^2 of DRFP[21]	R^2 of MFF[22]	R^2 of SEMG-MIGNN
Random 90/10	0.962 $\pm$ 0.040	0.965 $\pm$ 0.010	0.943 $\pm$ 0.010	**0.970 $\pm$ 0.010**
Random 80/20	0.957 $\pm$ 0.010	0.953 $\pm$ 0.005	0.931 $\pm$ 0.010	**0.971 $\pm$ 0.005**
Random 70/30	0.952 $\pm$ 0.005	0.951 $\pm$ 0.005	0.930 $\pm$ 0.010	**0.969 $\pm$ 0.005**
Random 60/40	0.934 $\pm$ 0.010	0.932 $\pm$ 0.010	0.918 $\pm$ 0.010	**0.963 $\pm$ 0.010**
Random 50/50	0.922 $\pm$ 0.010	0.929 $\pm$ 0.010	0.904 $\pm$ 0.010	**0.941 $\pm$ 0.010**
Random 40/60	0.901 $\pm$ 0.010	0.899 $\pm$ 0.010	0.881 $\pm$ 0.020	**0.921 $\pm$ 0.010**
Random 30/70	0.883 $\pm$ 0.010	0.897 $\pm$ 0.010	0.863 $\pm$ 0.010	**0.903 $\pm$ 0.010**
Random 20/80	0.862 $\pm$ 0.010	0.878 $\pm$ 0.010	0.839 $\pm$ 0.010	**0.883 $\pm$ 0.010**
Random 10/90	0.791 $\pm$ 0.020	0.813 $\pm$ 0.010	0.773 $\pm$ 0.010	**0.834 $\pm$ 0.010**
Test 1[a]	0.843 $\pm$ 0.010	0.809 $\pm$ 0.010	**0.853 $\pm$ 0.010**	0.848 $\pm$ 0.010
Test 2[a]	0.841 $\pm$ 0.030	0.832 $\pm$ 0.003	0.713 $\pm$ 0.005	**0.867 $\pm$ 0.010**
Test 3[a]	0.753 $\pm$ 0.040	0.710 $\pm$ 0.001	0.641 $\pm$ 0.005	**0.776 $\pm$ 0.020**
Test 4[a]	0.492 $\pm$ 0.050	0.491 $\pm$ 0.004	0.178 $\pm$ 0.010	**0.677 $\pm$ 0.020**
Avg.1-4	0.732	0.711	0.596	**0.792**

Note: The best values are shown in bold. [a]Tests 1 to 4 are the extrapolative tests of additives, whose data splitting are determined in Doyle's original study[25] and applied in other modelling studies[21-22, 24].

In addition, we have re-divided the yield datasets from the perspective of scaffold splitting, and evaluated the SOTA models in a series of prediction tasks. Supplementary Figure 20 shows the details of scaffold splitting in the yield dataset. For aryl halides, the substituted arenes were selected in the training set, and the pyridines were included in the test set. For Buchwald ligands, we chose the two ligands with the additional methoxy substituent as the training set and the rest two ligands as the test set. For base, the guanidine-type organic bases are used for the training set, and phosphazene are included in the test set. For the oxazole additives, we selected the mono-substituted ones as the training set and the di-substituted ones as the test set. The above scaffold-based splittings have clear organic chemistry meanings and pose extrapolative challenges from the synthetic perspective.

Supplementary Figure 21 and Supplementary Table 20 summarizes the results of the extrapolation tasks for yield prediction using the SEMG-MIGNN (Sterics- and Electronics-embedded Molecular Graph and Molecular Interaction Graph Neural Network), Yield-BERT[24], DRFP[21], and MFF models. SEMG-MIGNN (Sterics- and Electronics-embedded Molecular Graph and Molecular Interaction Graph Neural Network) model demonstrated noticeable advantage. The arene-to-pyridine extrapolation task of aryl halides is the most difficult among the four extrapolation challenges; SEMG-MIGNN (Sterics- and Electronics-embedded Molecular Graph and Molecular Interaction Graph Neural Network) achieved a regression performance with R^2 of 0.576, which is significantly higher than the R^2 of the other three models (0.230, Yield-BERT[24]; 0.222, DRFP[21]; 0.449, MFF). In the extrapolation tasks for additive, ligand, and base, the tested SOTA models also did not achieve satisfying regression performances, with R^2 ranging from 0.3 to 0.5, making it difficult to provide synthetically useful predictions. However, our SEMG-MIGNN model (Sterics- and Electronics-embedded Molecular Graph and Molecular Interaction Graph Neural Network) achieved R^2 of 0.851 in the additive task, 0.816 in the ligand task, and 0.658 in the base task.

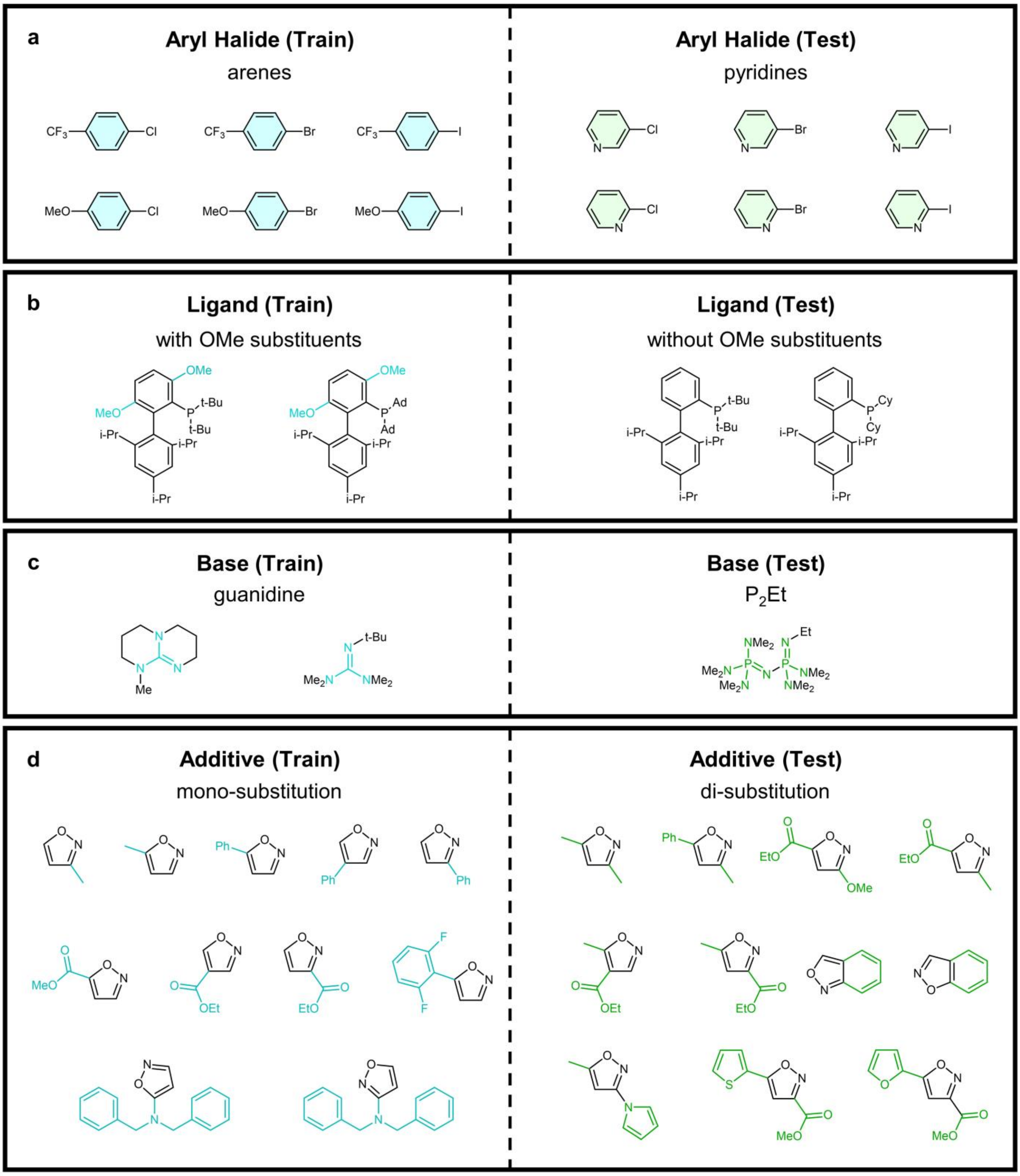

Supplementary Figure 20. Scaffold splitting of Doyle's yield dataset. The blue and green colours of molecules represent the differences between the training set (blue) and the test set (green). a For aryl halides, the substituted arenes were selected in the training set, and the pyridines were included in the test set. **b** For Buchwald ligands, we chose the two ligands with the additional methoxy substituent as the training set and the rest two ligands as the test set. **c** For base, the guanidine-type organic bases are used for the training set, and phosphazene are included in the test set. **d** For the oxazole additives, we selected the mono-substituted ones as the training set and the di-substituted ones as the test set.

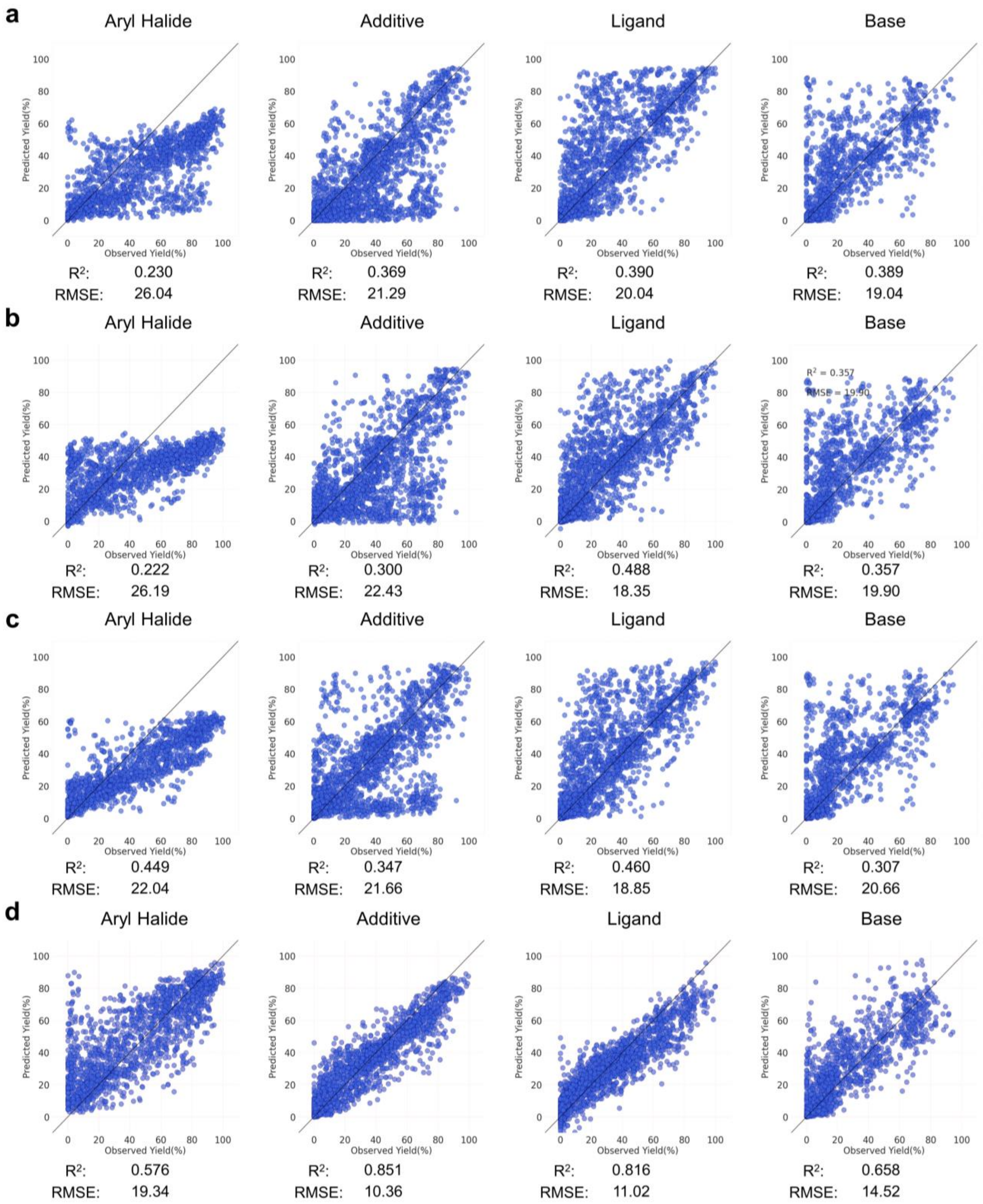

Supplementary Figure 21. Modelling results in the scaffold-based extrapolation tasks of yield prediction using SEMG-MIGNN (Sterics- and Electronics-embedded Molecular Graph and Molecular Interaction Graph Neural Network) and other SOTA models. a Regression performances of Yield-BERT model. **b** Regression performances of DRFP model (Differential Reaction Fingerprint). **c** Regression performances of MFF model (Multiple Fingerprint Feature). **d** Regression performances of SEMG-MIGNN model (Sterics- and Electronics-embedded Molecular Graph and Molecular Interaction Graph Neural Network). The data splittings are elaborated in Supplementary Figure 20.

Supplementary Table 20. Comparison of yield prediction between the SEMG-MIGNN model (Sterics- and Electronics-embedded Molecular Graph and Molecular Interaction Graph Neural Network) with other SOTA models in scaffold-based extrapolation tasks (in %). RMSE means Root Mean Square Error.

Data Splitting	Yield-BERT[24]		DRFP[21]		MFF[22]		SEMG-MIGNN	
	R^2	RMSE	R^2	RMSE	R^2	RMSE	R^2	RMSE
Aryl Halide	0.230	26.04	0.222	26.19	0.449	22.04	**0.576**	**19.34**
Additive	0.369	21.29	0.300	22.43	0.347	21.66	**0.851**	**10.36**
Ligand	0.390	20.04	0.488	18.35	0.460	18.85	**0.816**	**11.02**
Base	0.389	19.04	0.357	19.90	0.307	20.66	**0.658**	**14.52**

Note: The best values are shown in bold. The data splitting details of the scaffold-based extrapolation tasks are elaborated in Supplementary Information (Supplementary Figure 20).

4.3 Enantioselectivity prediction in asymmetric *N,S*-acetal formation

We also compared the SOTA models in 13 enantioselectivity prediction tasks. In addition to the 9 random data splitting tasks with different ratios of training data, we also divided the imines, thiols, and catalysts based on the molecular scaffold. Supplementary Figure 22 elaborates the details of these scaffold-based data splitting. The division of imines classified imine-5 with bicyclic naphthyl substituent as the test set, while only monocyclic aryl substituents were included in the training set. Thiols were classified to aliphatic thiols (test set) and aromatic thiols (training set). For the phosphoric acid catalysts, they were divided to the training set of BINAP CPAs and the test set of H_8-BINAP CPAs. In addition to these scaffold-based splitting, we also examined the transformation-based splitting; the 9 transformations involving imine-1 and thiol-1 were divided to the test set, while the remaining 16 transformations were used as the training set. The above data splitting posed a series of extrapolative challenges for the machine learning models and examined the prediction performances under application scenarios.

Supplementary Table 21 summarized the performances of DRFP, MFF, and SEMG-MIGNN models (Sterics- and Electronics-embedded Molecular Graph and Molecular Interaction Graph Neural Network). Yield-BERT was not considered because it was not developed for enantioselectivity prediction.[24] Our model presented noticeable improvements in most scenarios. In the random data splitting, only in the case of very limited training data (10% and 20% training data), SEMG-MIGNN (Sterics- and Electronics-embedded Molecular Graph and Molecular Interaction Graph Neural Network) is worse than DRFP and MFF. While in the other random data splitting scenarios, SEMG-MIGNN (Sterics- and Electronics-embedded Molecular Graph and Molecular Interaction Graph Neural Network) outcompeted the DRFP and MFF models. In the extrapolations of thiol and catalyst, DRFP and MFF showed poor or even incorrect predictions, while the predictions of SEMG-MIGNN (Sterics- and Electronics-embedded Molecular Graph and Molecular Interaction Graph Neural Network) are still competent without pitfall scenarios. For the transformation-out splitting, SEMG-MIGNN (Sterics- and Electronics-embedded Molecular Graph and Molecular Interaction Graph Neural Network) is also the only model with a R^2 over 0.9. These results demonstrated that the SEMG-MIGNN model

(Sterics- and Electronics-embedded Molecular Graph and Molecular Interaction Graph Neural Network) also has an outstanding performance in enantioselectivity prediction, especially in challenging extrapolation tasks.

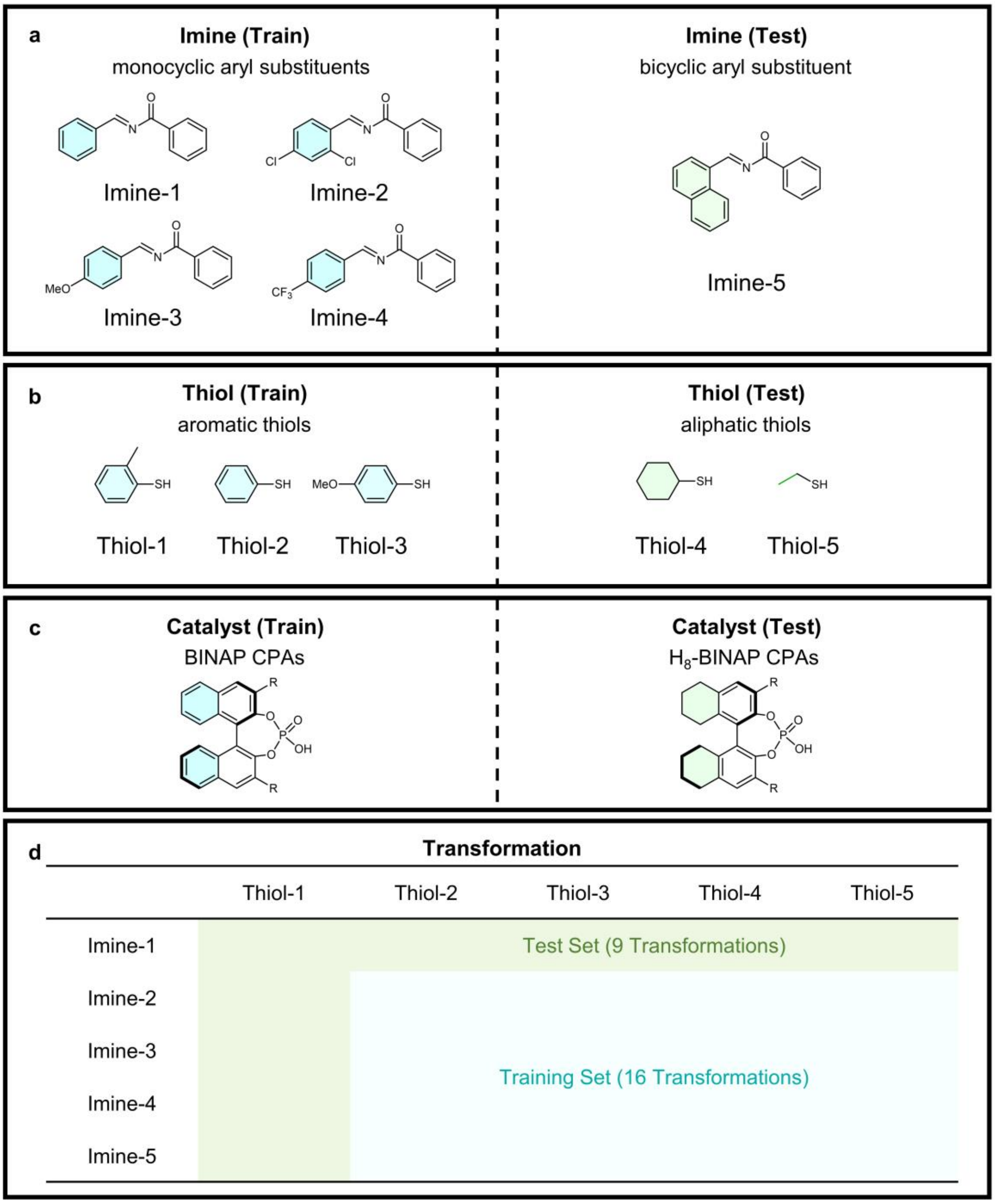

Supplementary Figure 22. Data splitting of Denmark's enantioselectivity dataset based on molecular scaffolds and transformations. The blue and green colours of molecules represent the differences between the training set (blue) and the test set (green). For the transformation, the blue shadings means training set and the green shadings means test set. a The division of imines classified imine-5 with bicyclic naphthyl substituent as the test set, and the monocyclic aryl substituents were included in the training set. **b** Thiols were classified to aliphatic thiols (test set) and aromatic thiols (training set). **c** For the phosphoric acid catalysts, they were divided to the training set of BINAP CPAs and the test set of H_8-BINAP CPAs. **d** For the transformation-based splitting; the 9 transformations involving imine-1 and thiol-1 were divided to the test set, while the remaining 16 transformations were used as the training set.

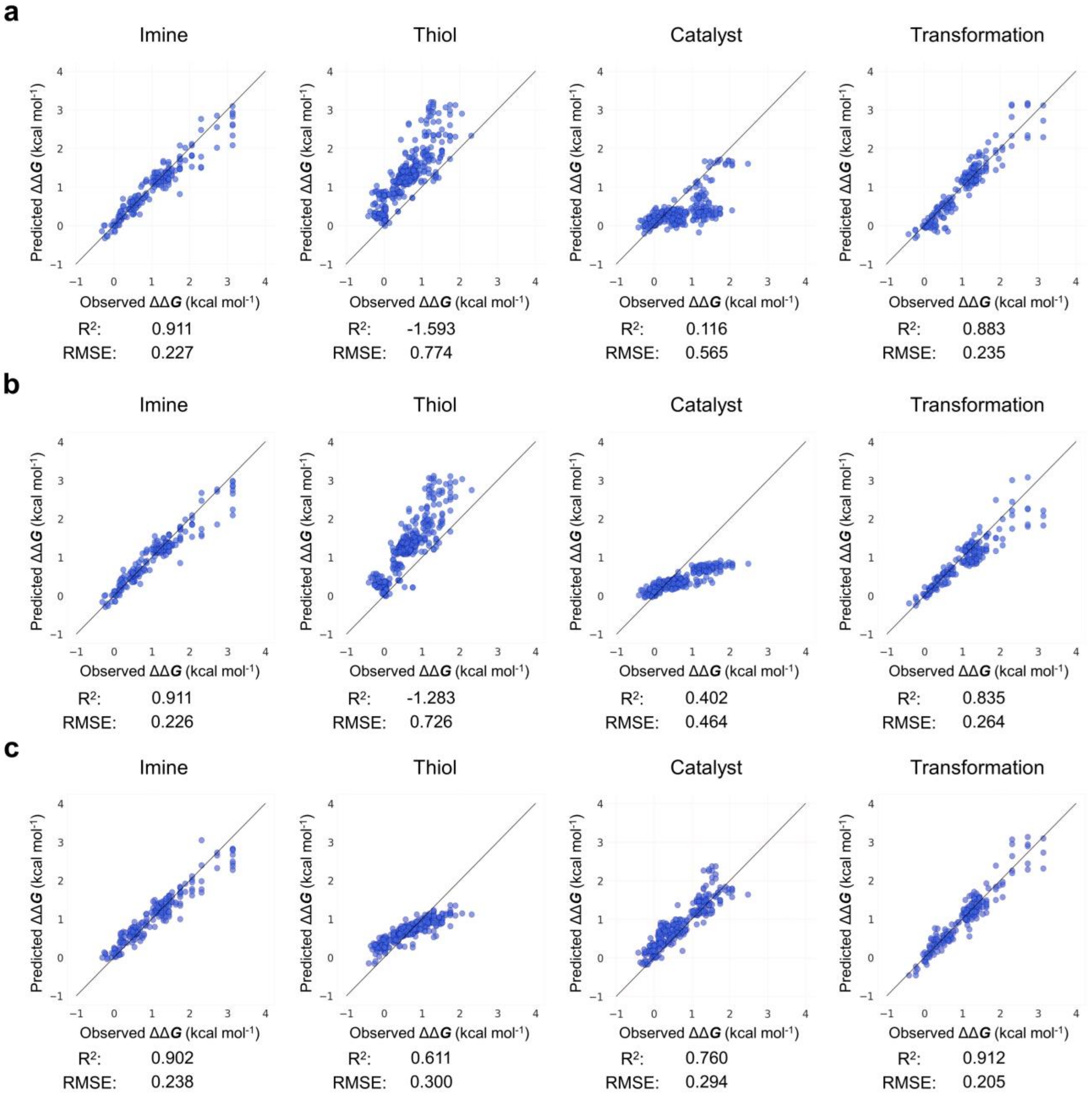

Supplementary Figure 23. Modelling results in the extrapolation tasks of enantioselectivity prediction using SEMG-MIGNN (Sterics- and Electronics-embedded Molecular Graph and Molecular Interaction Graph Neural Network) and other SOTA models. a Regression performances of DRFP model (Differential Reaction Fingerprint). **b** Regression performances of MFF model (Multiple Fingerprint Feature). **c** Regression performances of SEMG-MIGNN model (Sterics- and Electronics-embedded Molecular Graph and Molecular Interaction Graph Neural Network). RMSEs (Root Mean Square Error) are in kcal mol^{-1}. The data splittings are elaborated in Supplementary Figure 22.

Supplementary Table 21. Prediction performances of enantioselectivity tasks using various SOTA models. SEMG-MIGNN means Sterics- and Electronics-embedded Molecular Graph and Molecular Interaction Graph Neural Network. RMSE means Root Mean Square Error.

Data Splitting	DRFP[21]		MFF		SEMG-MIGNN	
	R^2	RMSE	R^2	RMSE	R^2	RMSE
Random 90/10	0.903 ± 0.010	0.190 ± 0.010	0.910 ± 0.010	0.183 ± 0.010	**0.927 ± 0.005**	**0.180 ± 0.010**
Random 80/20	0.890 ± 0.010	0.223 ± 0.010	0.908 ± 0.010	0.194 ± 0.020	**0.929 ± 0.005**	**0.179 ± 0.010**
Random 70/30	0.886 ± 0.010	0.201 ± 0.010	0.895 ± 0.010	0.212 ± 0.020	**0.920 ± 0.010**	**0.189 ± 0.010**
Random 60/40	0.873 ± 0.020	0.240 ± 0.020	0.890 ± 0.010	0.230 ± 0.020	**0.915 ± 0.010**	**0.195 ± 0.010**
Random 50/50	0.869 ± 0.020	0.248 ± 0.030	0.888 ± 0.020	0.227 ± 0.030	**0.905 ± 0.010**	**0.205 ± 0.020**
Random 40/60	0.864 ± 0.020	0.256 ± 0.030	0.885 ± 0.020	0.238 ± 0.030	**0.887 ± 0.010**	**0.221 ± 0.020**
Random 30/70	0.863 ± 0.020	0.259 ± 0.030	0.868 ± 0.020	0.243 ± 0.030	**0.872 ± 0.010**	**0.240 ± 0.020**
Random 20/80	0.833 ± 0.030	0.286 ± 0.040	**0.861 ± 0.030**	**0.258 ± 0.030**	0.834 ± 0.010	0.281 ± 0.020
Random 10/90	**0.823 ± 0.020**	**0.291 ± 0.020**	0.776 ± 0.030	0.426 ± 0.030	0.721 ± 0.010	0.370 ± 0.020
Imine[a]	0.904 ± 0.005	0.235 ± 0.005	**0.911 ± 0.005**	**0.226 ± 0.005**	0.902 ± 0.005	0.238 ± 0.005
Thiol[a]	-1.585 ± 0.020	0.773 ± 0.020	-1.283 ± 0.020	0.726 ± 0.020	**0.611 ± 0.010**	**0.300 ± 0.010**
Catalyst[a]	0.127 ± 0.020	0.561 ± 0.020	0.402 ± 0.020	0.464 ± 0.020	**0.760 ± 0.010**	**0.294 ± 0.010**
Transformation[a]	0.885 ± 0.005	0.233 ± 0.005	0.835 ± 0.005	0.264 ± 0.005	**0.912 ± 0.005**	**0.205 ± 0.005**

Note: The best values are shown in bold. [a]These data splitting tasks refer to the extrapolative predictions based on the scaffold splitting of the reaction components. Details are elaborated in Supplementary Figure 22. RMSEs (Root Mean Square Errors) are in kcal mol^{-1}.

5. Results of experiment

5.1 Experimental results of 11 new acids

The results of enantioselectivity and yield of the 11 new chiral phosphoric acid-catalyzed thiol addition to *N*-acylimines are shown in Supplementary Figure 24.

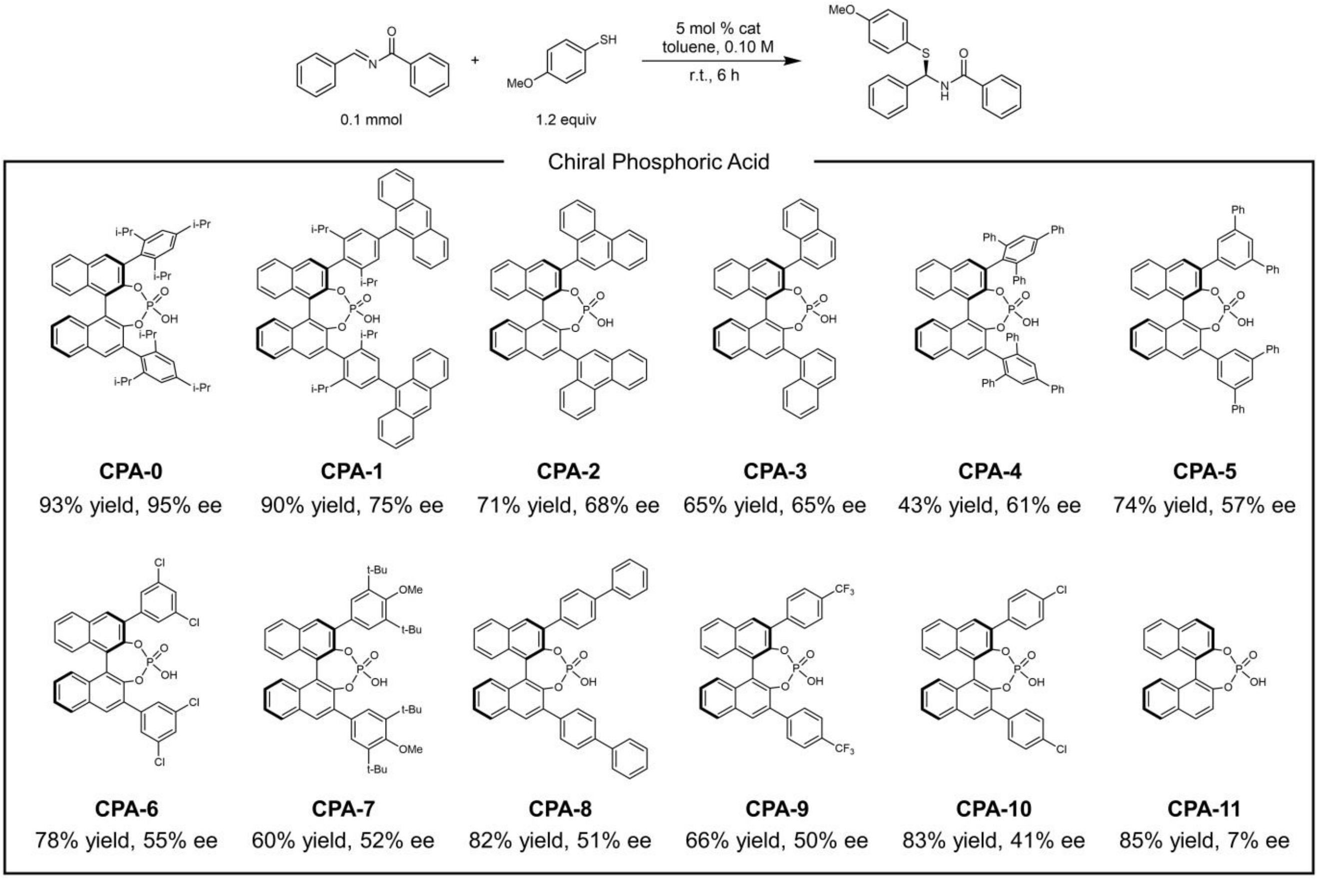

Supplementary Figure 24. Experimental results of 11 new acids, including yield and ee.

5.2 HPLC Spectra

CPA-0:

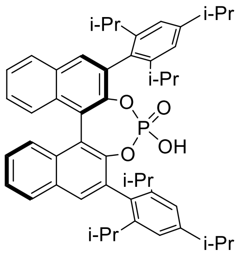

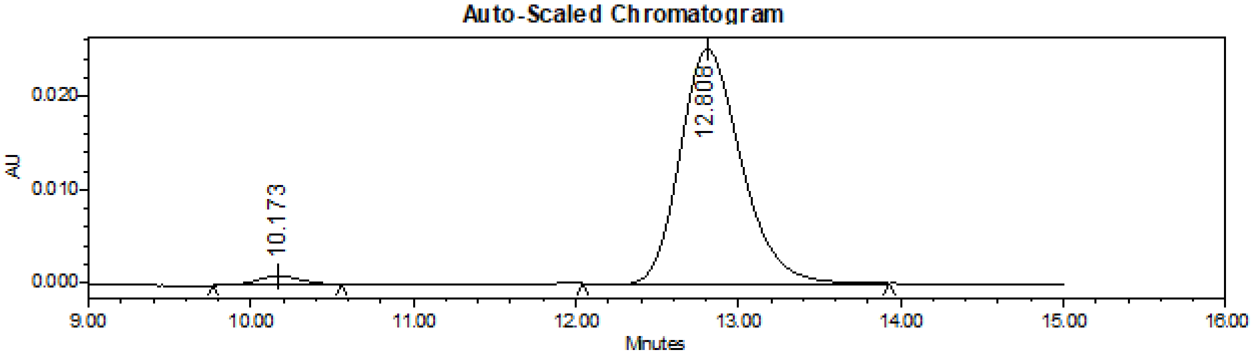

Peak Results

	RT	Area	% Area
1	10.173	18332	2.71
2	12.808	657376	97.29

CPA-1:

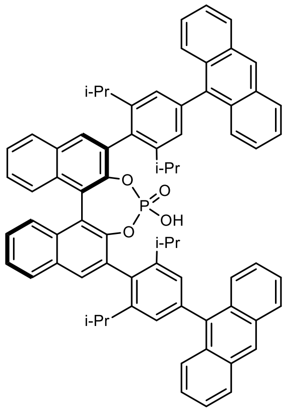

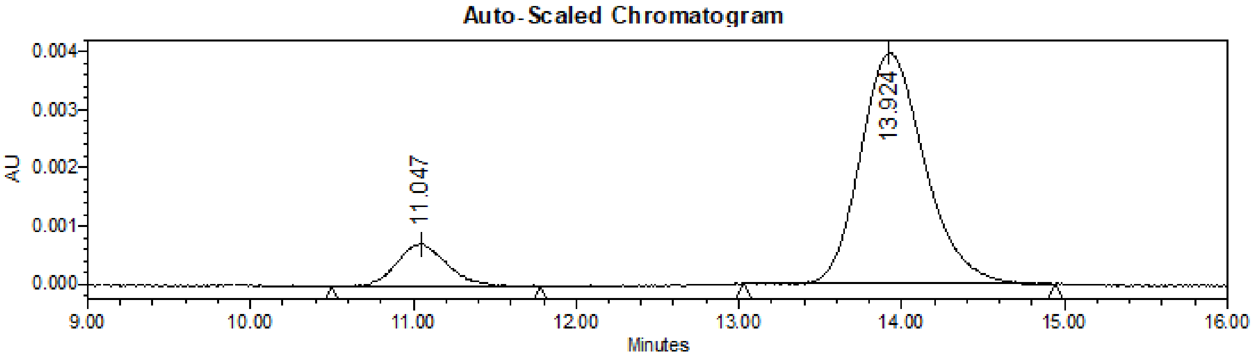

Peak Results

	RT	Area	% Area
1	11.047	15777	12.64
2	13.924	109075	87.36

CPA-2:

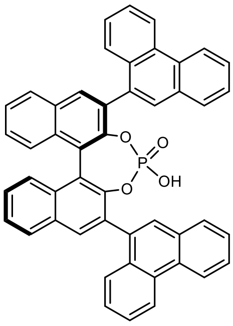

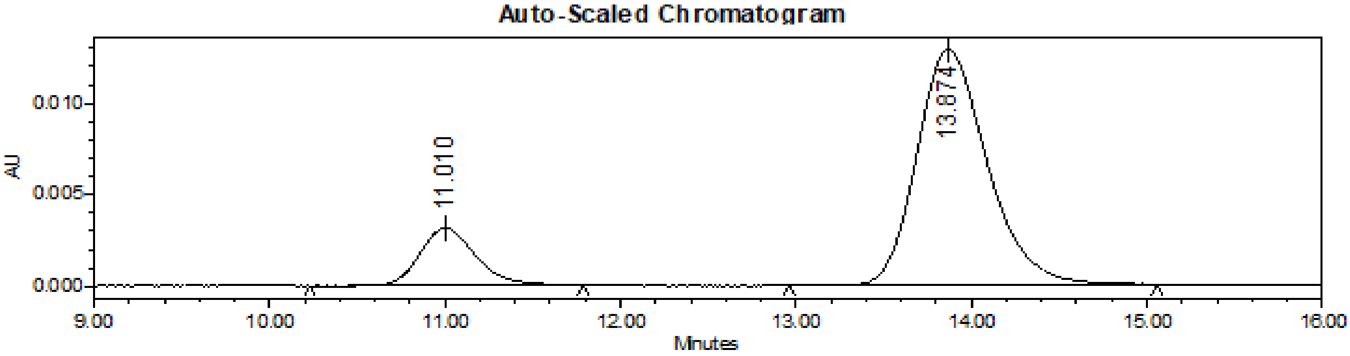

Peak Results

	RT	Area	% Area
1	11.010	68945	16.17
2	13.874	357331	83.83

CPA-3:

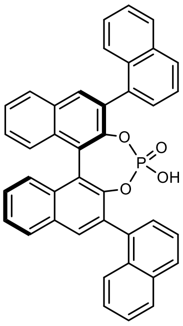

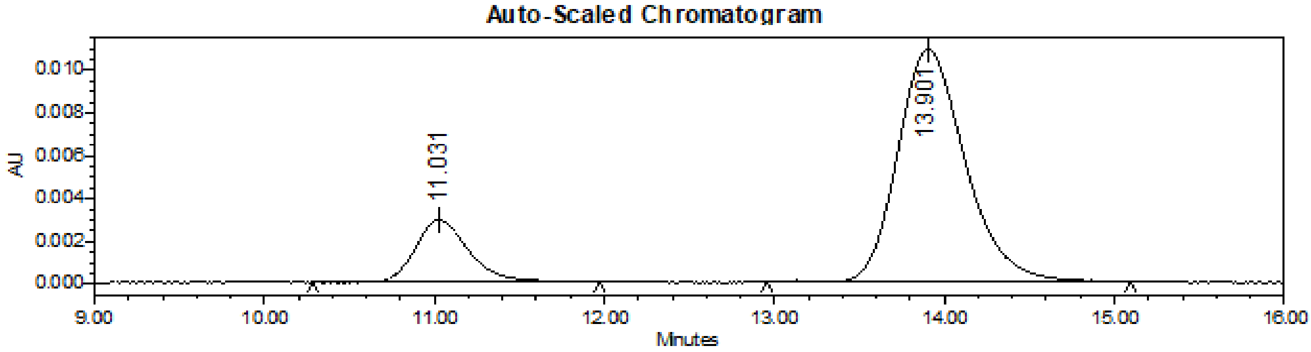

Peak Results

	RT	Area	% Area
1	11.031	62985	17.34
2	13.901	300329	82.66

CPA-4:

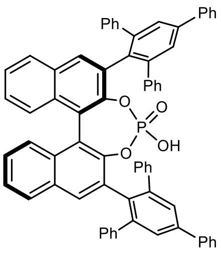

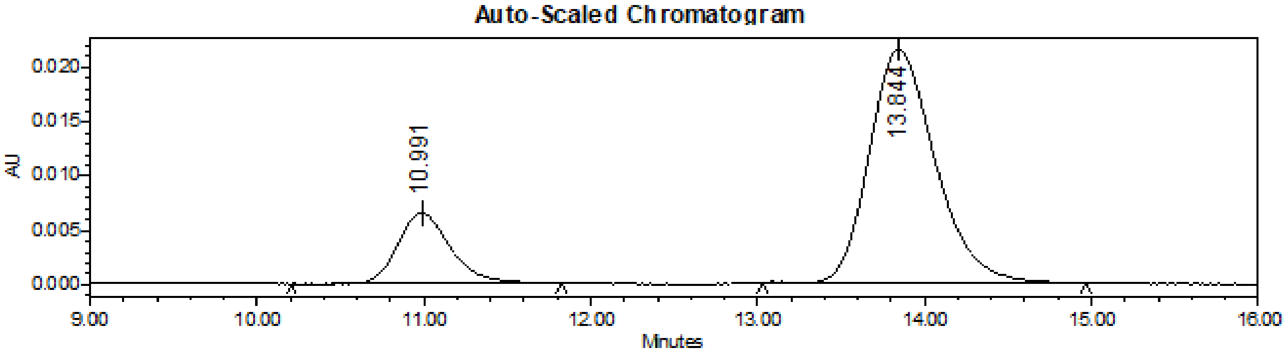

Peak Results

	RT	Area	% Area
1	10.991	142948	19.51
2	13.844	589629	80.49

CPA-5:

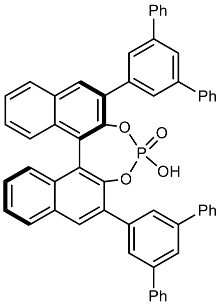

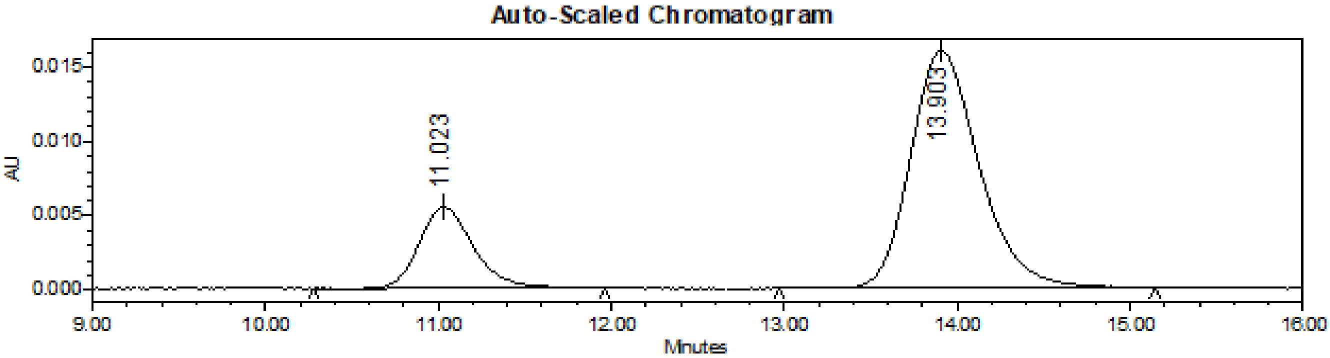

Peak Results

	RT	Area	% Area
1	11.023	120731	21.47
2	13.903	441535	78.53

CPA-6：

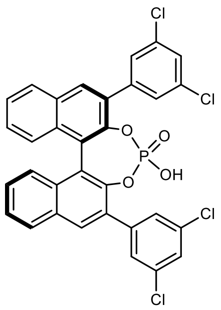

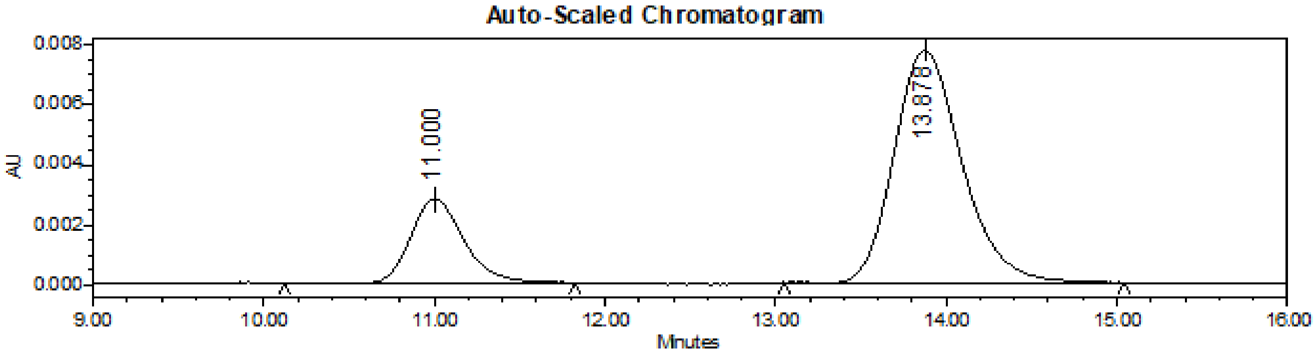

Peak Results

	RT	Area	% Area
1	11.000	61448	22.34
2	13.878	213595	77.66

CPA-7：

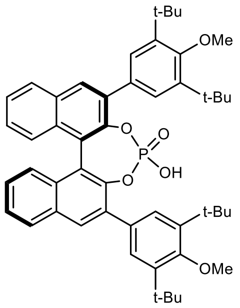

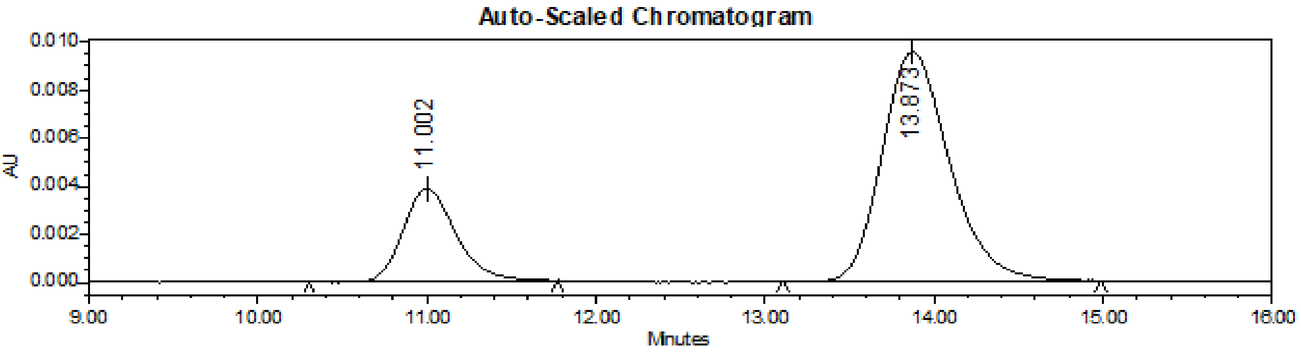

Peak Results

	RT	Area	% Area
1	11.002	83755	24.13
2	13.873	263287	75.87

CPA-8:

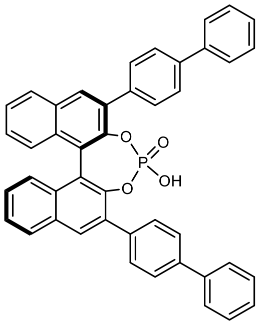

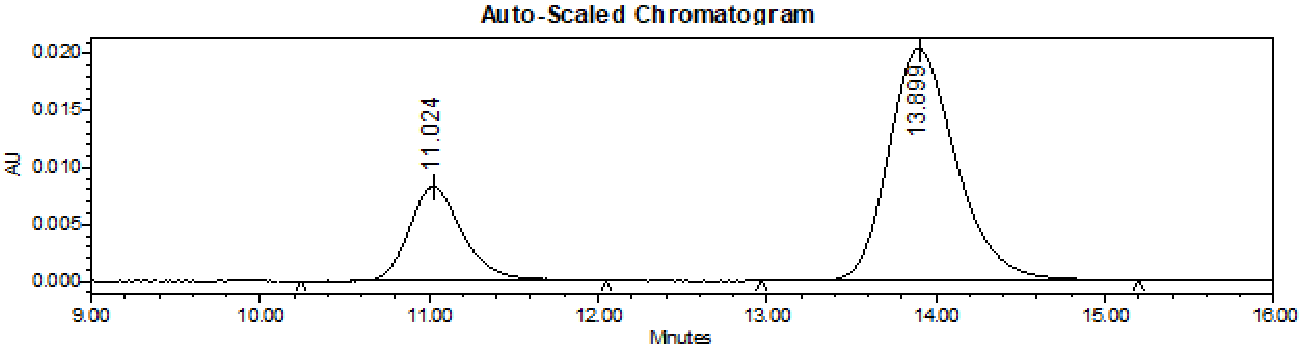

Peak Results

	RT	Area	% Area
1	11.024	181587	24.31
2	13.899	565325	75.69

CPA-9:

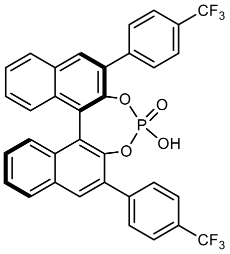

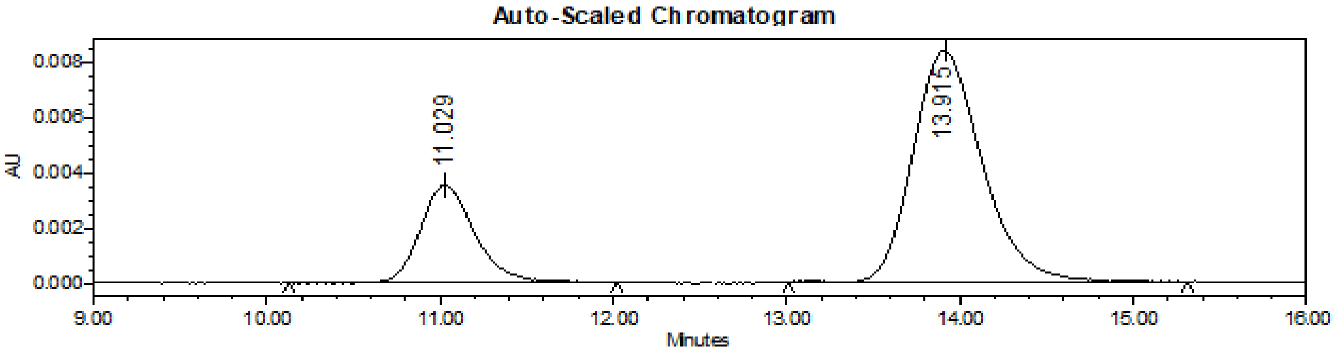

Peak Results

	RT	Area	% Area
1	11.029	76953	25.09
2	13.915	229815	74.91

CPA-10:

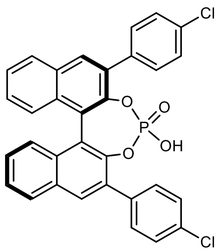

Auto-Scaled Chromatogram

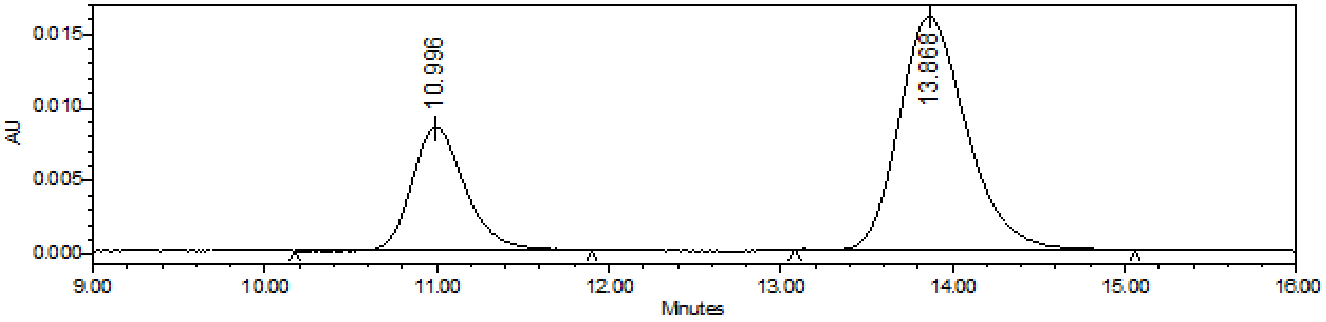

Peak Results

	RT	Area	% Area
1	10.996	180534	29.46
2	13.868	432353	70.54

CPA-11:

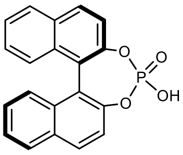

Auto-Scaled Chromatogram

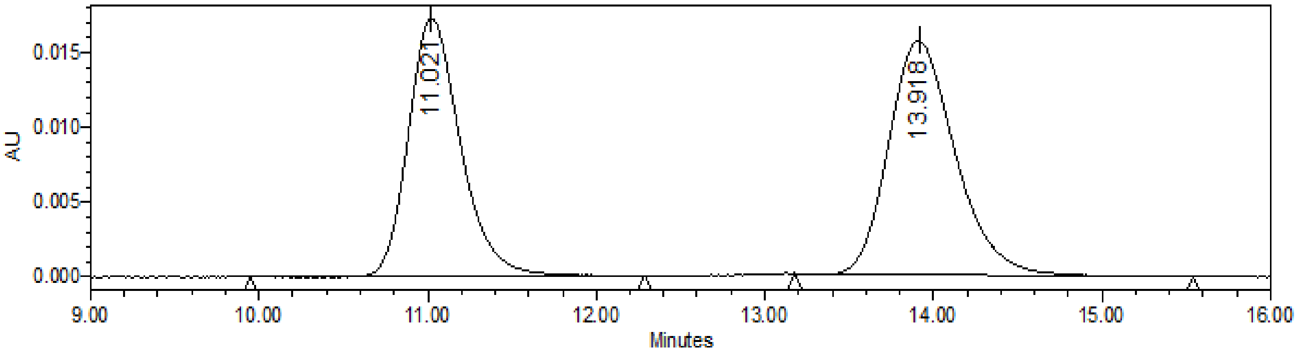

Peak Results

	RT	Area	% Area
1	11.021	372670	46.39
2	13.918	430695	53.61

6. Data and code availability

All the involved codes and data in this study were freely available at https://github.com/Shuwen-Li/SEMG-MIGNN.

Supplementary references

1. Rdkit: Open-source chemoinformatics and machine learning. http://www.rdkit.org.
2. Rogers D. & Hahn M. Extended-connectivity fingerprints. *J. Chem. Inf. Model.* **50**, 742–754 (2010).
3. Himanen L., *et al.* Dscribe: Library of descriptors for machine learning in materials science. *Comput. Phys. Commun.* **247**, 106949 (2020).
4. Pedregosa F., *et al.* Scikit-learn: Machine learning in python. Preprint at https://www.semanticscholar.org/paper/Scikit-learn%3A-Machine-Learning-in-Python-Pedregosa-Varoquaux/168f28ac3c8c7ea63bf7ed25f2288e8b67e2fe74 (2018).
5. Behler J. Atom-centered symmetry functions for constructing high-dimensional neural network potentials. *J. Chem. Phys.* **134**, 074106 (2011).
6. Freund Y. & Schapire R. E. A decision-theoretic generalization of on-line learning and an application to boosting. *J. Comput. Syst. Sci.* **55**, 119-139 (1997).
7. Skurichina M. & Duin R. P. W. Bagging for linear classifiers. *Mach. Learn.* **24**, 123-140 (1996).
8. Breiman L., Friedman J., Olshen R. & Stone C. Classification and regression trees. *Encyclopedia of Ecology* **57**, 582-588 (2015).
9. Geurts P., Ernst D. & Wehenkel L. Extremely randomized trees. *Mach. Learn.* **63**, 3-42 (2006).
10. Jerome H. F. Greedy function approximation: A gradient boosting machine. *Ann. Stat.* **29**, 1189–1232 (2001).
11. Fix E. & Hodges J. L. Discriminatory analysis. Nonparametric discrimination: Consistency properties. *Int. Stat. Rev.* **57**, 238–247 (1989).
12. Cawley G. C. & Talbot N. L. C. Reduced rank kernel ridge regression. *Neural Processing Lett.* **16**, 293‐302 (2002).
13. Cortes C. & Vapnik V. Support-vector networks. *Mach. Learn.* **20**, 273–297 (1995).
14. Biau G. Analysis of a random forests model. *J. Mach. Learn. Res.* **13**, 1063-1095 (2012).
15. Garcia C. B., Garcia J., Martin M. M. L. & Salmeron R. Collinearity: Revisiting the variance inflation factor in ridge regression. *J. Appl. Stat.* **42**, 648-661 (2015).
16. Zhang Y. & Chen L. A study on forecasting the default risk of bond based on xgboost algorithm and over-sampling method. *Theor. Econ. Lett.* **11**, 258-267 (2021).
17. Hinton G. E. Connectionist learning procedures. *Artif. Intell.* **40**, 185–234 (1989).
18. Xgb: Scalable and flexible gradient boosting. https://xgboost.ai/.
19. Deep graph library. https://www.dgl.ai.
20. Zahrt A. F., *et al.* Prediction of higher-selectivity catalysts by computer-driven workflow and machine learning. *Science* **363**, eaau5631 (2019).
21. Probst D., Schwaller P. & Reymond J. L. Reaction classification and yield prediction using the differential reaction fingerprint drfp. *Digit. Discov.* **1**, 91-97 (2022).
22. Sandfort F., Strieth-Kalthoff F., Kühnemund M., Beecks C. & Glorius F. A structure-based platform for predicting chemical reactivity. *Chem* **6**, 1379-1390 (2020).
23. Xu L. C., *et al.* Towards data-driven design of asymmetric hydrogenation of olefins: Database and hierarchical learning. *Angew. Chem. Int. Ed.* **60**, 22804-22811 (2021).
24. Schwaller P., Vaucher A. C., Laino T. & Reymond J.-L. Prediction of chemical reaction yields using deep learning. *Mach. Learn.* **2**, (2021).
25. Ahneman D. T., Estrada J. G., Lin S., Dreher S. D. & Doyle A. G. Predicting reaction performance in c–n cross-coupling using machine learning. *Science* **360**, 186-190 (2018).
